# The burden of nonalcoholic fatty liver disease (NAFLD) is rapidly growing in every region of the world from 1990 to 2019

**DOI:** 10.1097/HC9.0000000000000251

**Published:** 2023-10-02

**Authors:** James M. Paik, Linda Henry, Youssef Younossi, Janus Ong, Saleh Alqahtani, Zobair M. Younossi

**Affiliations:** 1 Beatty Liver and Obesity Research Program, Inova Health System, Falls Church, Virginia, USA; 2 Department of Medicine, Center for Liver Disease, Inova Fairfax Medical Campus, Falls Church, Virginia, USA; 3 Center for Outcomes Research in Liver Diseases, Washington DC, USA; 4 University of the Philippines, College of Medicine, Manila, Philippines; 5 Johns Hopkins Medical Center, Baltimore, Maryland, USA; 6 The Global NASH Council, Washington DC, USA

## Abstract

**Background:**

The latest meta-analyses suggest NAFLD is increasing globally. Its limitations may preclude accurate estimates. We evaluated the global NAFLD burden and its’ trends in prevalence and NAFLD liver–related mortality (LRM) by sex, age, region, and country over the past 3 decades using data from the Global Burden of Disease (GBD) 2019 study.

**Methods:**

Crude and age-standardized NAFLD prevalence and NAFLD-LRM rates were obtained for all-age individuals with NAFLD from 204 countries/territories between 1990 and 2019. Joinpoint trend analysis assessed time trends. Weighted average of the annual percent change (APC) over the period 1990–2019 and 2010–2019 were reported.

**Results:**

All-age (children and adults) crude global NAFLD prevalence increased:10.5% (561 million)–16.0% (1,236 million); an APC increase: + 1.47% (95% CI, 1.44%, 1.50%). Among adults (+20 y), crude NAFLD prevalence increased (1990: 17.6%, 2019:23.4%; APC: + 1.00%, 95% CI: 0.97%, 1.02%). In all-age groups, the crude NAFLD-LRM rate (per 100,000) increased (1990: 1.75%, 2019: 2.18%; APC: + 0.77% (95% CI, 0.70%, 0.84%). By Joinpoint analysis, from 2010 to 2019, worsening all-age trends in NAFLD prevalence and LRM were observed among 202 and 167 countries, respectively. In 2019, there were 1.24 billion NAFLD prevalent cases and 168,969 associated deaths; Asia regions accounted for 57.2% of all-age prevalent cases and 46.2% of all-age NAFLD-LRM. The highest all-age crude NAFLD prevalence rate was the Middle East and North Africa (LRM 26.5%); the highest all-age crude NAFLD-LRM rate was Central Latin America (5.90 per 100,000).

**Conclusions:**

NAFLD is increasing globally in all-age groups—over 80% of countries experienced an increase in NAFLD and NAFLD-LRM. These data have important policy implications for affected countries and for global health.

## Graphical abstract

**Figure:**
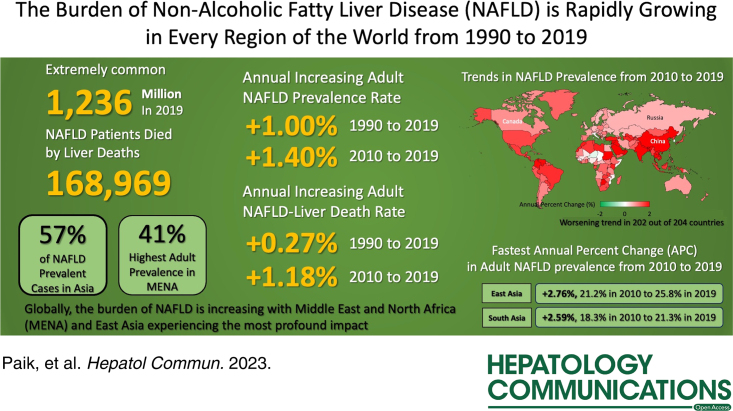

## INTRODUCTION

NAFLD is increasing almost in parallel to the increasing rates of obesity and type 2 diabetes (T2D) and is recognized as one of the most common causes of chronic liver diseases among adults, with a current estimated global prevalence of 30%.^1–13^ NAFLD is highly prevalent in children and adolescents, with an estimated global prevalence of 7.4%.^14^

The progressive form of NAFLD or NASH can lead to liver mortality and is associated with impaired health-related quality of life, decreased worker productivity, and increased health care resource utilization.^15–29^ Although lifestyle modification is the mainstay of current treatment for NAFLD and NASH, a number of new drug regimens are being developed.^16,21–36^ Over the past 3 decades, the prevalence of NAFLD has grown substantially.^1–15^ Nevertheless, awareness about NAFLD is very low, and NAFLD is not recognized by World Health Organization as an important noncommunicable disease.

There are different ways to assess the burden of NAFLD. Traditionally, meta-analysis is used to estimate the prevalence and mortality of NAFLD.^9,14^ Although powerful analytic tools, meta-analysis can frequently suffer from high heterogeneity and lack data from some countries, which may result in data being biased towards certain large regions of the world. On the other hand, the Global Burden of Disease (GBD) database, coordinated by the Institute for Health Metrics and Evaluation (IHME), provides a systematic scientific assessment of data on incidence, prevalence, and mortality for a mutually exclusive list of diseases and injuries at a national level. Using complex statistical models adjusted for levels of uncertainty and the noise of imperfect data, these GBD assessments are then provided as estimates of each outcome rather than as raw data points.^37–40^

Importantly, IHME also annually updates each GBD study to incorporate newly available data sets, enhance their method performance, and adopt changes in the scientific understanding of the disease. In this context, the results of GBD data analysis could become more complete and accurate over time. Consequently, GBD 2019 (published in 2020) is the most up-to-date version of GBD and supersedes all prior versions (GBD 1990, 2020, 2013, 2015, 2016, and 2017).^41–43^

Previous studies have investigated the burden of liver complications based on GBD 2017 and found that NAFLD has been the most rapidly growing contributor to liver complications over the last decade.^4,8,39,40^ However, GBD 2017 has been unable to provide the prevalence of NAFLD. Fortunately, with the updated GBD version, GBD 2019, IHME has provided prevalence data on NAFLD. An appropriate understanding of GBD, its analysis, as well as its strengths and limitations are critical to arrive at valid conclusions. To date, none of the previous studies based on GBD 2019 presented a detailed and updated GBD methodology for NAFLD prevalence. These details are critical to assure the conclusion of these analyses is valid.^44–47^ Therefore, the purpose of the current study is to evaluate patterns in NAFLD prevalence and NAFLD liver-related mortality (LRM) using GBD 2019 with an updated and detailed methodology description.

## METHOD

### Overview of GBD estimation framework

GBD estimation process started with identifying multiple sources for each disease, including census, household surveys, vital registration, verbal autopsy, cancer registry data, health service use, and other sources through a systematic review of published studies, searches of government and international organization websites, demography and health surveys, and contributions of data from GBD collaborator. Relevant metadata can be retrieved through the publicly available Data Input Sources Tool (https://ghdx.healthdata.org/gbd-2019/data-input-sources). Processed data are then modeled using statistical models [Cause of Death Ensemble model (CODEm), spatiotemporal Gaussian process regression (ST-GPR), meta and DisMod-MR] to estimate outcomes of interest for age-sex-location and year amid the noise of imperfect data and to ensure consistency between incidence, prevalence, remission, excess mortality, and cause-specific mortality for most causes.^48^ As a result, GBD 2019 updates and expands the results obtained from GBD 2017, which will now provide more accurate estimates of NAFLD prevalence. Thus, we present the GBD estimation methods for NAFLD prevalence and updates in GBD 2019. In addition, we will provide the quality of the data by country to assist policymakers, health care workers, and readers better appreciate the results of these analyses.

This study was approved and received Exempt Status by the Internal Review Board, Inova Health System.

### NAFLD prevalence

For the first time, GBD 2019 reported NAFLD prevalence estimates for age-sex-location and years by estimating NAFLD prevalence by aggregating the following 3 domains, including the prevalence of NAFLD without cirrhosis, liver cancer due to NAFLD, cirrhosis due to NAFLD, and NAFLD-LRM.^49^

### NAFLD without cirrhosis

To obtain data for NAFLD prevalence, GBD used a systematic literature review which included studies that had a sample size ≥ 100 and sufficient description of methods for assessing the quality of the study, did not exclude comorbidities in their sample, used ultrasound or other diagnostic imaging methods for establishing the diagnosis of NAFLD, and had a representative sample of the general population for the location. GBD excluded studies relying solely on administrative data from hospitals or claims data, with an exception in Asia studies where the participation rate for health checkups is high, and ultrasonography is available as a part of the medical checkup. Included 52 studies are available at GBD 2019 Data Input Sources Tool (https://ghdx.healthdata.org/gbd-2019/data-input-sources).

GBD modeled the prevalence of NAFLD using DisMod-MR2.1, an integrative meta-regression method to obtain age-sex-location-year specific estimates with predictive covariates (including mean body mass index, prevalence of obesity, and age-standardized scaled exposure variables for high fasting plasma glucose).

A complete list of predictive covariates used in the models can be found in Supplemental Table 1, http://links.lww.com/HC9/A501.^42,43^ In GBD 2019, obesity and high fasting plasma glucose were newly added. Basically, associations between predictive covariates and NAFLD prevalence for age-sex-location-year combinations were used to help predict NAFLD prevalence for age-sex-location-year combinations with little or no data. Furthermore, the following post hoc adjustment was applied. GBD found that most of the studies excluded patients with excessive alcohol consumption from their sample. As a result, the reported prevalence rates from these studies reflected the prevalence of non-excessive alcohol users, not the general population. GBD used the proportion of the general population that consumes < 70 g of alcohol per week for females and < 140 g for males to adjust the reported year-age-sex and location-specific prevalence rate to reflect the prevalence in the general population. However, similar post hoc adjustments for studies that excluded other chronic liver diseases from their samples were not performed (yet). Also, GBD reported that the effect of using different definitions of excessive alcohol consumption in the NAFLD definition was insignificant.

### Cirrhosis due to NAFLD

In 2019, GBD separately estimated the prevalence and mortality of cirrhosis due to NAFLD and the proportion of cirrhosis attributable to HBV, HCV, alcohol [alcohol-associated liver disease (ALD)], NAFLD or NASH, haemochromatosis, autoimmune hepatitis, Wilson’s disease, cryptogenic, idiopathic, or unknown. Also, GBD modeled cirrhosis (ICD 10 codes: K70-K77, I85, I98.2, and R18) prevalence based on hospital claims (n = 334) using DisMod-MR 2.1 with predictive covariates including Hepatitis B Seroprevalence (HBsAg), Hepatitis C Seroprevalence (anti-HCV), alcohol consumed per capita, prevalence obesity, and Health care Access and Quality index, and zero remission. Finally, GBD modeled cirrhosis and liver cancer mortality separately based on the cause of death database, a compilation of data assembled from a variety of primary source documents (vital registration, sample vital registration, and verbal autopsy), using the cause of death ensemble model are described in detail in another publication.^38^

To estimate etiology-specific cirrhosis prevalence and mortality, GBD developed etiological proportion models using DisMod-MR 2.1 based on studies that reported the proportion of cirrhosis due to 5 etiologies (HBV, HCV, ALD, NAFLD, and Other) from a systematic review. Estimated proportions were applied to split the initial cirrhosis prevalence and mortality estimates according to etiology-specific prevalence and mortality.^50,51^ GBD included studies that had the publication year of 1980 or later and sufficient description of methods for assessing the quality of the study, was a representative sample of patients with cirrhosis, and used standard diagnosis of HBV and HCV.

Compared with GBD 2017, GBD 2019 updated the method of modelling the proportion of cirrhosis due to NAFLD versus “other causes.” In GBD 2017, cryptogenic cases were extracted as “other causes” when a study did not explicitly identify NASH. Since epidemiological studies have indicated that cryptogenic cases may be unidentified cases of NASH in GBD 2019, GBD modeled the proportion due to NASH out of the combined group of cryptogenic plus NASH cases. This modeled proportion was then used to extract NASH cases when the studies reported only cryptogenic cases but not NASH cases.

### Liver cancer due to NAFLD

GBD estimated mortality due to liver cancer and the prevalence of liver cancer using the mortality-to-incidence ratios (MIRs) based on the cause of death database and modelled by using spatiotemporal Gaussian process regression.^52^ The estimated liver cancer mortality and mortality-to-incidence ratios were used to estimate the incidence of liver cancer. Prevalence was estimated using incidence and survival estimates.

To determine etiological proportions of liver cancers for age-sex-location-year combinations, the proportion data found through the systematic literature review were used as input for 5 separate DisMod-MR 2.1 models. Unlike cirrhosis modeling, cases, where etiology was listed as cryptogenic, idiopathic, or unknown, were considered “other causes.” The etiological proportions were multiplied by estimates of liver cancer and prevalence to etiology-specific estimates.

Cancer mortality estimates in GBD 2019 updated the GBD 2017 data in multiple ways. Most importantly, GBD 2019 used updates based on the clinical informatics data (inpatient admission, outpatient encounters, and insurance claims) for adjustment using a correction factor estimated from MarketScan, a database of claims data for commercial insurance in the United States. This adjustment changed all study-level covariates leading to large differences in the incidence and prevalence between GBD 2017 and GBD 2019.

In the GBD database, for administrative and data analysis purposes, the world is divided into 21 GBD regions according to epidemiological similarities and geographical proximity. Age-standardized rates were based on the world standard population developed for the GBD study. Of note, the age-standardized rate is only available for all ages (children and adults) in GBD. GBD estimates for a disease burden are reported with 95% uncertainty intervals, including the true value of a parameter with 95% probability. Uncertainty intervals account for not only variance in parameter estimation but also uncertainty from data collection, model selection, and other sources of uncertainty under the parameter estimation process.

### Data analysis

Results and findings of GBD 2019 can be explored interactively through the GBD Visualization Hub.^38^ Temporal trends in rates were assessed by Joinpoint trend analysis using the National Cancer Institute’s software using the SEMs, obtained by the width of 95% uncertainty intervals divided by 1.96*2.^52^ A maximum number of 5 joinpoints were allowed, and the modified Bayes information criterion was used for model selection.^53^ From the selected models, the annual percentage change (APC) for each trend segment and the entire period 1990–2019 were reported with a 95% CI. Since joinpoint analysis detected a precipitous rise in prevalence and death rate during the last recent time segment, we also provided an APC over the last decade, 2010–2019. The weighted average of the APC over the period 1990–2019 and 2010–2019 from the joinpoint models were reported. The increasing or decreasing trend was defined if the APC was significantly different from 0; otherwise, a stable or level trend was defined. Microsoft Excel was used for data visualization. Countries with ≥ 20 million population size were denoted by the asterisk symbol (*).

## RESULTS

The reported NAFLD prevalent cases, prevalence, NAFLD-LRM, and death rate are summarized for all ages (children and adults) except where otherwise noted.

### NAFLD burden in 2019

#### NAFLD cases and NAFLD-LRM

In 2019, worldwide, there were a total of 1.24 billion prevalent cases of NAFLD and 168,969 NAFLD-LRM for all ages. Both prevalent cases and deaths were more common in men (Table 1). Among adults (aged 20 or years older), the global NAFLD prevalent cases and LRM reported by GBD 2019 were 1.21 billion cases and 168,566 deaths (Table 2).

**TABLE 1:** Among all ages (children and adults), NAFLD prevalent cases, crude prevalence rate, NAFLD-related liver mortality, crude mortality rate, and annual percent change in rates during 1990–2019 and 2010–2019

	Prevalent cases (Prevalence %)			APC (95% CI)	
	1990	2010	2019	1990–2019	2010–2019
Global	561,366,824 (10.49)	958,781,491 (13.72)	1,235,699,718 (15.97)	1.47 (1.44, 1.50)	1.75 (1.69, 1.80)
Sex					
Males	309,499,362 (11.49)	522,832,002 (14.89)	679,286,300 (17.50)	1.45 (1.43, 1.47)	1.80 (1.76, 1.83)
Females	251,867,461 (9.48)	435,949,489 (12.54)	556,413,418 (14.43)	1.51 (1.44, 1.59)	1.70 (1.57, 1.83)
Region					
Australasia	1,624,919 (8.01)	2,856,678 (11.04)	3,460,100 (11.91)	1.37 (1.34, 1.40)	0.82 (0.80, 0.84)
High-income North America	23,246,580 (8.28)	36,801,272 (10.74)	44,316,610 (12.16)	1.34 (1.31, 1.36)	1.40 (1.35, 1.45)
High-income Asia Pacific	13,535,203 (7.80)	18,679,483 (10.10)	20,940,590 (11.18)	1.21 (1.07, 1.36)	1.00 (0.79, 1.20)
Southern Latin America	3,140,551 (6.34)	5,433,572 (8.88)	6,483,932 (9.71)	1.48 (1.45, 1.51)	0.96 (0.94, 0.99)
Western Europe	37,026,088 (9.63)	54,280,513 (12.85)	59,006,466 (13.52)	1.16 (1.13, 1.20)	0.53 (0.46, 0.59)
Central Europe	14,758,541 (12.00)	17,645,673 (15.06)	18,628,411 (16.31)	1.06 (1.06, 1.07)	0.89 (0.88, 0.90)
Eastern Europe	28,742,280 (12.69)	32,546,807 (15.37)	34,111,363 (16.25)	0.85 (0.84, 0.86)	0.62 (0.60, 0.64)
Central Asia	6,860,544 (9.90)	10,106,415 (12.25)	12,815,306 (13.70)	1.13 (1.11, 1.15)	1.27 (1.25, 1.29)
Southeast Asia	56,613,903 (12.13)	101,868,137 (16.47)	128,069,888 (19.01)	1.56 (1.56, 1.57)	1.61 (1.60, 1.63)
East Asia	138,889,890 (11.34)	232,240,656 (16.43)	303,125,668 (20.59)	2.12 (1.99, 2.24)	2.76 (2.52, 3.00)
Oceania	702,208 (10.85)	1,328,167 (12.41)	1,744,123 (13.14)	0.66 (0.65, 0.67)	0.65 (0.64, 0.66)
South Asia	95,443,036 (8.70)	169,135,884 (10.66)	241,842,616 (13.40)	1.51 (1.42, 1.61)	2.59 (2.41, 2.77)
Andean Latin America	3,227,341 (8.45)	6,378,816 (11.81)	8,442,452 (13.28)	1.57 (1.55, 1.58)	1.30 (1.29, 1.31)
Caribbean	4,341,266 (12.31)	7,017,608 (15.94)	8,176,331 (17.33)	1.19 (1.18, 1.21)	0.94 (0.92, 0.95)
Central Latin America	17,059,656 (10.39)	33,401,494 (14.58)	42,166,980 (16.87)	1.68 (1.68, 1.69)	1.63 (1.62, 1.64)
Tropical Latin America	15,654,055 (10.24)	29,996,957 (14.67)	37,986,673 (16.99)	1.76 (1.75, 1.77)	1.65 (1.62, 1.67)
North Africa and Middle East	60,106,799 (17.42)	123,303,691 (23.47)	161,460,206 (26.52)	1.47 (1.43, 1.50)	1.38 (1.36, 1.41)
Central Sub- Saharan Africa	4,246,738 (7.65)	8,090,240 (7.96)	11,344,041 (8.62)	0.42 (0.40, 0.44)	0.91 (0.86–0.95)
Eastern Sub- Saharan Africa	13,972,063 (7.35)	25,837,299 (7.91)	36,093,949 (8.77)	0.61 (0.59, 0.63)	1.15 (1.10, 1.19)
Southern Sub- Saharan Africa	6,093,904 (11.61)	10,487,515 (14.79)	13,162,068 (16.75)	1.27 (1.26, 1.29)	1.40 (1.37, 1.44)
Western Sub- Saharan Africa	16,081,260 (8.35)	31,344,615 (8.88)	42,321,947 (9.27)	0.36 (0.35, 0.37)	0.48 (0.45, 0.51)
	NAFLD-LRM (Mortality Rate per 100,000)			APC (95% CI)	
	1990	2010	2019	1990, 2019	2010, 2019
Global	93,758 (1.75)	132,562 (1.90)	168,969 (2.18)	0.77 (0.70, 0.84)	1.59 (1.42, 1.77)
Sex					
Males	48,688 (1.81)	69,940 (1.99)	89,763 (2.31)	0.87 (0.81, 0.94)	1.72 (1.63, 1.81)
Females	45,070 (1.70)	62,622 (1.80)	79,206 (2.05)	0.67 (0.60, 0.74)	1.50 (1.31, 1.68)
Region					
Australasia	259 (1.28)	486 (1.88)	637 (2.19)	1.90 (1.66, 2.14)	1.73 (1.34, 2.12)
High-income North America	4,488 (1.60)	7,113 (2.08)	9,903 (2.72)	1.85 (1.66, 2.04)	2.94 (2.54, 3.34)
High-income Asia Pacific	2,310 (1.33)	3,508 (1.90)	3,984 (2.13)	1.59 (1.45, 1.73)	1.07 (0.90, 1.23)
Southern Latin America	936 (1.89)	1,275 (2.08)	1,630 (2.44)	0.93 (0.73, 1.12)	1.79 (1.40, 2.18)
Western Europe	10,607 (2.76)	11,528 (2.73)	12,359 (2.83)	0.09 (−0.02, 0.19)	0.38 (0.25, 0.50)
Central Europe	2,205 (1.79)	2,684 (2.29)	2,658 (2.33)	0.90 (0.73, 1.08)	0.20 (−0.13, 0.52)
Eastern Europe	2,977 (1.31)	8,069 (3.81)	8,174 (3.89)	3.95 (3.03, 4.87)	0.07 (−1.30, 1.45)
Central Asia	969 (1.40)	2,441 (2.96)	3,069 (3.28)	3.00 (2.78, 3.23)	1.07 (0.79, 1.35)
Southeast Asia	10,733 (2.30)	18,508 (2.99)	23,704 (3.52)	1.48 (1.38, 1.57)	1.83 (1.74, 1.92)
East Asia	21,510 (1.76)	19,006 (1.34)	25,413 (1.73)	−0.03 (−0.26, 0.20)	2.91 (2.68, 3.15)
Oceania	47 (0.73)	83 (0.77)	107 (0.81)	0.33 (0.23, 0.43)	0.48 (0.37, 0.58)
South Asia	11,572 (1.05)	15,611 (0.98)	21,896 (1.21)	0.55 (0.31, 0.78)	2.40 (2.05, 2.76)
Andean Latin America	1,250 (3.27)	2,469 (4.57)	3,133 (4.93)	1.41 (1.10, 1.71)	0.77 (0.48, 1.06)
Caribbean	1,057 (3.00)	1,282 (2.91)	1,773 (3.76)	0.82 (0.57, 1.07)	2.91 (2.26, 3.56)
Central Latin America	6,039 (3.68)	11,121 (4.86)	14,757 (5.90)	1.66 (1.37, 1.95)	2.22 (1.74, 2.71)
Tropical Latin America	2,072 (1.36)	3,622 (1.77)	4,828 (2.16)	1.66 (1.30, 2.02)	2.27 (1.51, 3.05)
North Africa and Middle East	6,019 (1.74)	10,690 (2.03)	14,476 (2.38)	1.10 (0.96, 1.25)	1.81 (1.72, 1.90)
Central Sub- Saharan Africa	740 (1.33)	1,127 (1.11)	1,554 (1.18)	−0.39 (−0.55−, -0.22)	0.92 (0.56, 1.28)
Eastern Sub- Saharan Africa	3,541 (1.86)	5,083 (1.56)	6,726 (1.63)	−0.47 (−0.53−, 0.41)	0.54 (0.45, 0.62)
Southern Sub- Saharan Africa	700 (1.33)	1,299 (1.83)	1,322 (1.68)	0.81 (0.47, 1.17)	−0.72 (−1.09, 0.35)
Western Sub- Saharan Africa	3,727 (1.94)	5,557 (1.57)	6,865 (1.50)	−0.87 (−1.01, −0.74)	−0.53 (−0.64, −0.43)

Abbreviation: APC, annual percent change; NAFLD-LRM, NAFLD-LRM.

**TABLE 2:** Among adults (+ 20 years), NAFLD prevalent cases, crude prevalence rate, NAFLD-related liver mortality, crude mortality rate, and annual percent change in rates during 1990–2019 and 2010–2019

	Prevalent cases (Prevalence %)			APC (95% CI)	
	1990	2010	2019	1990–2019	2010–2019
Global	541,998,304 (17.62)	931,368,866 (20.74)	1,206,538,206 (23.39)	1.00 (0.97, 1.02)	1.40 (1.35, 1.46)
Sex					
Males	297,136,714 (19.43)	505,388,487 (22.73)	660,742,269 (25.90)	0.98 (0.96, 1.01)	1.44 (1.40, 1.48)
Females	244,861,590 (15.83)	425,980,378 (18.79)	545,795,937 (20.94)	1.03 (0.94, 1.12)	1.34 (1.12, 1.57)
Region					
Australasia	1,579,650 (11.28)	2,792,989 (14.71)	3,394,082 (15.53)	1.10 (1.07, 1.14)	0.58 (0.55, 0.60)
High-income North America	22,620,572 (11.34)	35,813,556 (14.28)	43,322,838 (15.78)	1.15 (1.12, 1.17)	1.12 (1.06, 1.18)
High-income Asia Pacific	13,110,525 (10.65)	18,328,625 (12.35)	20,653,624 (13.33)	0.73 (0.61, 0.84)	0.72 (0.51, 0.92)
Southern Latin America	3,039,862 (10.08)	5,272,934 (12.87)	6,323,217 (13.52)	1.02 (0.99, 1.04)	0.53 (0.52, 0.55)
Western Europe	36,165,965 (12.64)	53,374,396 (16.15)	58,084,826 (16.88)	0.99 (0.95, 1.02)	0.45 (0.38, 0.51)
Central Europe	14,463,857 (17.14)	17,388,705 (18.96)	18,403,049 (20.26)	0.58 (0.57, 0.59)	0.73 (0.73, 0.74)
Eastern Europe	28,218,916 (17.73)	32,058,002 (19.22)	33,674,328 (20.72)	0.54 (0.52, 0.56)	0.83 (0.78, 0.87)
Central Asia	6,599,590 (17.49)	9,712,990 (19.08)	12,442,259 (20.89)	0.61 (0.59, 0.63)	1.01 (0.97, 1.05)
Southeast Asia	54,512,513 (22.20)	99,136,133 (25.68)	125,205,117 (27.93)	0.80 (0.79, 0.81)	0.95 (0.94, 0.97)
East Asia	134,255,369 (17.67)	227,449,801 (21.15)	299,143,103 (25.76)	1.36 (1.21, 1.52)	2.54 (2.24, 2.84)
Oceania	668,542 (21.16)	1,268,576 (22.79)	1,675,881 (23.38)	0.34 (0.33, 0.35)	0.27 (0.25, 0.28)
South Asia	92,625,840 (16.85)	164,321,539 (18.27)	236,119,508 (21.26)	0.81 (0.75, 0.88)	1.71 (1.57, 1.84)
Andean Latin America	3,085,817 (16.21)	6,130,668 (19.16)	8,152,126 (20.42)	0.80 (0.79, 0.81)	0.71 (0.70, 0.72)
Caribbean	4,201,653 (20.82)	6,835,833 (24.13)	7,989,450 (25.26)	0.67 (0.66, 0.69)	0.51 (0.50, 0.52)
Central Latin America	16,319,895 (19.94)	32,233,066 (23.51)	40,926,199 (25.17)	0.81 (0.80, 0.81)	0.76 (0.75, 0.78)
Tropical Latin America	15,204,734 (18.26)	29,349,975 (21.84)	37,319,346 (23.80)	0.92 (0.90, 0.93)	0.98 (0.95, 1.01)
North Africa and Middle East	56,599,312 (34.36)	117,422,974 (38.17)	155,207,646 (40.85)	0.60 (0.58, 0.62)	0.77 (0.75, 0.79)
Central Sub- Saharan Africa	4,061,698 (16.92)	7,719,866 (17.28)	10,835,635 (17.94)	0.21 (0.19, 0.22)	0.43 (0.40, 0.46)
Eastern Sub- Saharan Africa	13,366,407 (16.81)	24,659,293 (17.49)	34,465,330 (18.31)	0.30 (0.28, 0.31)	0.50 (0.49, 0.52)
Southern Sub- Saharan Africa	5,841,878 (22.23)	10,102,265 (24.67)	12,761,886 (26.61)	0.62 (0.60, 0.63)	0.86 (0.83, 0.88)
Western Sub- Saharan Africa	15,455,709 (18.13)	29,996,681 (18.97)	40,438,756 (19.44)	0.24 (0.22, 0.25)	0.27 (0.23, 0.31)
	NAFLD-LRM (Mortality Rate per 100,000)			APC (95% CI)	
	1990	2010	2019	1990–2019	2010-2019
Global	93,358 (3.03)	132,147 (2.94)	168,566 (3.27)	0.27 (0.20, 0.33)	1.18 (1.04, 1.33)
Sex					
Males	48,479 (3.17)	69,714 (3.14)	89,543 (3.51)	0.37 (0.31, 0.43)	1.29 (1.21, 1.38)
Females	44,879 (2.90)	62,433 (2.75)	79,022 (3.03)	0.16 (0.10, 0.22)	1.10 (0.94, 1.25)
Region					
Australasia	259 (1.85)	485 (2.56)	637 (2.91)	1.61 (1.37, 1.85)	1.45 (1.06, 1.85)
High-income North America	4,485 (2.25)	7,109 (2.84)	9,899 (3.60)	1.65 (1.41, 1.88)	2.49 (1.97, 3.01)
High-income Asia Pacific	2,306 (1.87)	3,507 (2.36)	3,983 (2.57)	1.06 (0.92, 1.20)	0.75 (0.58, 0.92)
Southern Latin America	935 (3.10)	1,274 (3.11)	1,629 (3.48)	0.43 (0.19, 0.67)	1.28 (0.91, 1.66)
Western Europe	10,602 (3.70)	11,524 (3.49)	12,356 (3.59)	−0.11 (-0.24, 0.02)	0.27 (0.13, 0.40)
Central Europe	2,202 (2.61)	2,682 (2.93)	2,657 (2.93)	0.39 (0.21, 0.56)	0.02 (−0.30, 0.35)
Eastern Europe	2,972 (1.87)	8,065 (4.83)	8,171 (5.03)	3.74 (2.83, 4.67)	0.63 (0.17, 1.10)
Central Asia	964 (2.55)	2,430 (4.77)	3,061 (5.14)	2.45 (2.20, 2.70)	0.67 (0.32, 1.02)
Southeast Asia	10,669 (4.35)	18,449 (4.78)	23,644 (5.27)	0.67 (0.58, 0.77)	1.11 (1.03, 1.20)
East Asia	21,439 (2.82)	18,985 (1.76)	25,399 (2.19)	−0.86 (-1.05, −0.66)	2.48 (2.28, 2.67)
Oceania	47 (1.48)	82 (1.47)	107 (1.49)	−0.01 (−0.10, 0.09)	0.07 (−0.04, 0.17)
South Asia	11,447 (2.08)	15,453 (1.72)	21,750 (1.96)	−0.14 (−0.38, 0.10)	1.52 (1.16, 1.88)
Andean Latin America	1,246 (6.54)	2,465 (7.70)	3,129 (7.84)	0.61 (0.31, 0.92)	0.14 (−0.15, 0.42)
Caribbean	1,054 (5.22)	1,281 (4.52)	1,771 (5.60)	0.27 (0.02, 0.53)	2.43 (1.77, 3.09)
Central Latin America	6,024 (7.36)	11,106 (8.10)	14,742 (9.07)	0.74 (0.45, 1.02)	1.28 (0.81, 1.76)
Tropical Latin America	2,064 (2.48)	3,615 (2.69)	4,822 (3.08)	0.78 (0.40 , 1.15)	1.56 (0.77, 2.35)
North Africa and Middle East	5,993 (3.64)	10,659 (3.47)	14,443 (3.80)	0.16 (0.01, 0.31)	1.03 (0.93, 1.12)
Central Sub- Saharan Africa	733 (3.05)	1,116 (2.50)	1,541 (2.55)	−0.58 (−0.81, −0.35)	0.41 (−0.17, 0.99)
Eastern Sub- Saharan Africa	3,519 (4.42)	5,048 (3.58)	6,686 (3.55)	−0.77 (−0.83, −0.72)	−0.10 (−0.19, −0.00)
Southern Sub- Saharan Africa	696 (2.65)	1,294 (3.16)	1,317 (2.75)	0.13 (−0.21, 0.48)	−1.33 (−1.70, −0.96)
Western Sub- Saharan Africa	3,703 (4.34)	5,520 (3.49)	6,820 (3.28)	−0.96 (−1.09, −0.82)	−0.69 (−0.79, −0.59)

Abbreviation: APC, annual percent change.

Of 21 GBD regions, the highest number of NAFLD cases and NAFLD-LRM were observed in East Asia (303.13 million prevalent cases and 25,413 deaths), followed by South Asia (241.84 million and 21,896 deaths). Middle East and North Africa (MENA) had the (161.46 million and 14,476 deaths). Within MENA, Egypt accounted for 52.4% of NAFLD-LRM, with 18.9% of the prevalent cases. Southeast Asia had the fourth highest number of prevalent cases (128.07 million) but ranked second for NAFLD-LRM (23,704 deaths). Globally in 2019, Asia regions accounted for 57.2% of the global prevalent cases and 46.2% of the global NAFLD-LRM. This was mainly driven by China, India, and Indonesia. On the other hand, Western Europe ranked fifth for NAFLD prevalent cases (59.0 million) and sixth for NAFLD-LRM (12,359 deaths).

#### All-age crude NAFLD prevalence rate and NAFLD-LRM rate

In addition to cases, we assessed prevalence and LRM rates. In 2019, the global NAFLD prevalence rate and NAFLD-LRM rate (per 100,000) were 15.97% and 2.18 per 100,000, respectively. In this context, the highest NAFLD prevalence (%) was observed in MENA (26.5%), while the MENA region experienced the 10th highest NAFLD-LRM rate (3.84 per 100,000). Surprisingly, the highest NAFLD-LRM rate was observed in Central Latin America (5.90 per 100,000) and Andean Latin America (4.93 per 100,000). These high rates were observed despite a lower NAFLD prevalence rate.

**FIGURE 1:**
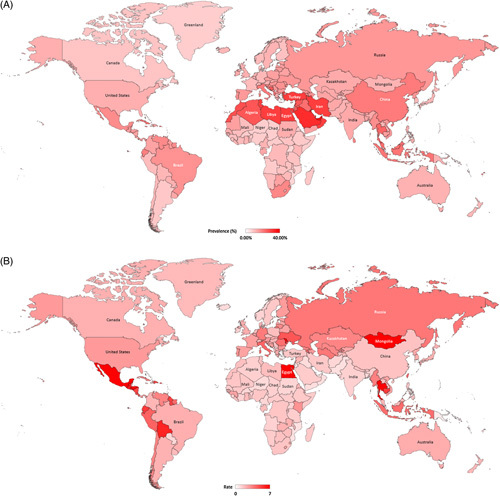
All-age crude NAFLD Prevalence and NAFLD-related liver mortality rate in 2019 worldwide. (A) All-age crude NAFLD prevalence rate (%). (B) All-age crude NAFLD-liver mortality rate (per 100,000). Source Data: Global Burden of Disease 2019.

At the country level, United Arab Emirates, Qatar, Bahrain, Kuwait, Saudi Arabia*, Egypt*, Turkey*, Iran*, Tunisia, and Libya had the highest NAFLD prevalence (≥30.2%), whereas the top 10 countries with the lowest NAFLD prevalence rate were (in ascending order) Uganda*, Mozambique*, Rwanda, Somalia*, Chad, Niger*, Greenland, Burundi, Nigeria*, and Angola* (<8.25%) (Figure 1A).

The top 10 countries with the highest NAFLD-LRM rate (>6.0 per 100,000) were Mongolia, Mexico*, Egypt*, Honduras, Moldova, Thailand*, Puerto Rico, Virgin Islands, United States, Guatemala, and Bolivia (Figure 1B). In contrast, the top 10 countries with the least NAFLD-LRM rate (<0.87 per 100, 000) were (in ascending order) Papua New Guinea, Equatorial Guinea, Kuwait, Brunei, United Arab Emirates, Singapore, Jordan, Mozambique*, Pakistan*, and Oman.

In addition to the prevalence rates according to countries, we also assessed LRM rates at the country level. At the country level of these regions, the highest NAFLD-LRM rate (≥6.0 per 100,000) was observed in Mexico, Honduras, Guatemala, and Bolivia. In contrast, Central, Eastern, and Western Sub-Saharan Africa and Southern Latin America had the lowest NAFLD prevalence (<10%), and African regions, South Asia, and Oceania had the lowest NAFLD-LRM rate (<1.70 per 100,000) (Figure 1B).

#### All-age age-standardized NAFLD prevalence rate and NAFLD-LRM rate

Although these were crude rates, different patterns were observed in age-standardized rates (Supplemental Figure 1A, http://links.lww.com/HC9/A502 and 1B, http://links.lww.com/HC9/A505). In this context, the lowest age-standardized prevalence rate and LRM rate were observed in High-income regions, including Western Europe, Australasia, High-income North America, Southern Latin America, and High-income Asia Pacific (<10% and <1.5 per 100,000).

### Changes in the prevalence rates for NAFLD from 1990 through 2019

**FIGURE 2:**
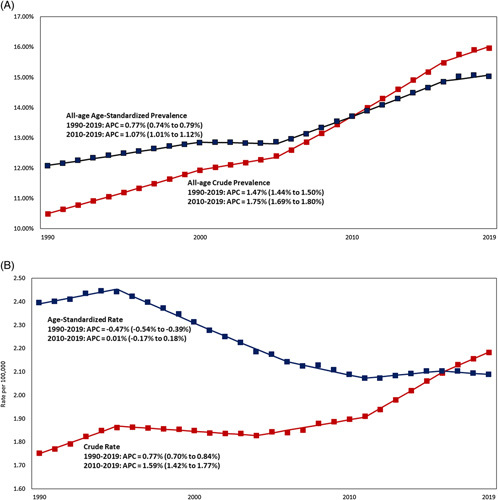
Trends in global NAFLD prevalence and NAFLD-liver mortality rate from 1990 to 2019. (A) Trends in all-age NAFLD prevalence rate (%). (B) Trends in all-age NAFLD-liver mortality rate. Source Data: Global Burden of Disease 2019.

From 1990 through 2019, the crude global NAFLD prevalence among all ages (children and adults) continuously increased from 10.5% (561.37 million) to 16.0% (1, 235.70 million) with an average annual increase of + 1.47% (95% CI, 1.44%, 1.50%) (Table 1, Figure 2A-B). Similarly, the all-age age-standardized global NAFLD prevalence increased by APC = + 0.77% (0.74%, 0.79%) from 12.1% to 15.0% (Supplemental Table 2, http://links.lww.com/HC9/A501). Among adults (+ 20 y), crude Global NAFLD prevalence increased by APC = + 1.00% (0.97%, 1.02%) from 17.6%, 23.4% (Table 2).

Joinpoint analysis showed that the annual increase in all-age crude global NAFLD prevalence rate sped up from APC = + 0.70% (95% CI, 0.59%, 0.81%) during the 2000–2005 period to APC = + 2.09% (2.07%, 2.12%) during the 2005–2016 period and APC = + 1.06 (0.90%, 1.22%) for the 2016–2019 (test on slope difference, *p* < 0.001). (Supplemental Table 3, http://links.lww.com/HC9/A501).

During the last decade (2010–2019), the all-age crude global NAFLD prevalence rate annually increased by APC = + 1.75% (1.69%, 1.80%) from 13.7% to 16.0%. The pattern of all-age crude NAFLD prevalence rate was similar in males (APC = + 1.80%, 1.76%, 1.83%) and females (APC = + 1.70%, 1.57%, 1.83%), although males had a consistently higher prevalence rate than females in most of the regions with exceptions of the Central and Tropical Latin America. The male-to-female ratio for all-age age-standardized NAFLD prevalence was 1.21 at the Global level, ranging from the highest in the High-income Asia-Pacific region at 2.20 to the lowest in Tropical Latin America at 0.96 (Data not shown).

**FIGURE 3:**
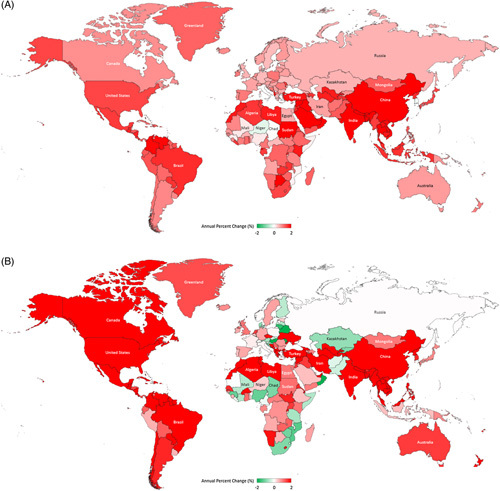
Trends in all-age crude NAFLD prevalence and NAFLD-liver deaths from 2010 to 2019 worldwide. (A) Annual percent change (%) in all-age crude NAFLD prevalence rate. (B) Annual percent change (%) in all-age crude NAFLD-liver mortality rate. Source Data: Global Burden of Disease 2019.

It is important to note that every region of the world experienced an increase in the NAFLD prevalence rate (Table 1 and Figure 3A). The 2 regions with the largest contributions to the global increasing trend in all-age crude NAFLD prevalence were East Asia (APC = + 2.76%, 95% CI, 2.52%, 3.00%) and South Asia (APC = + 2.59%, 2.41%, 2.77%)—faster than the global trend. Within these regions, the most important countries to this trend are India and China (APC of ≥ + 2.8%) (Supplemental Table 4, http://links.lww.com/HC9/A501).

During the 2010–2019 period, a worsening trend (APC of ≥ 0%) in crude all-age NAFLD prevalence rate was observed among 202 out of 204 countries (Figure 3A-B and Supplemental Table 4, http://links.lww.com/HC9/A501). The top 10 countries with a substantial increase (APC of ≥ 2.34%) were Maldives, India*, China*, Nepal*, Syria, Sudan*, Oman*, Saudi Arabia*, Bangladesh*, and Bhutan. The 2 countries with an improvement trend (APC of < 0%) were South Korea* and Niger*. Joinpoint analysis showed that in South Korea*, NAFLD prevalence peaked in 2004 and has dropped substantially (APC = − 2.16%, − 3.35%, − 0.96% for the 2014–2019 period) (Data not shown). Slightly different patterns were observed in all-age age-standardized NAFLD prevalence. See Supplementary information for all-age age-standardized prevalence results (Supplemental Table 5, http://links.lww.com/HC9/A501 and Supplemental Figure 2A, http://links.lww.com/HC9/A503).

### Changes in NAFLD-LRM rates, including cirrhosis and liver cancer, from 1990 to 2019

Among all ages (Children and Adults), from 1990 through 2019, the crude global NAFLD-LRM rate annually increased by + 0.77% (0.70%, 0.84%) from 1.75 to 2.18 (Table 1 and Figure 2B). Joinpoint analysis showed that since 2004, there has been a marked shift towards a substantial increase in crude all-age global NAFLD-LRM and accelerated over the recent time periods: 2011–2016 (APC = 1.93%, 1.67%, 2.18%) and 2016–2019 (APC = 1.36%, 0.97%, 1.75%) (Supplemental Table 6, http://links.lww.com/HC9/A501).

From 2010 to 2019, the all-age crude global NAFLD-LRM rate per 100,000 annually increased by + 1.59% (1.42%, 1.53%) from 1.90 to 2.18, whereas the all-age age-standardized NAFLD-LRM rate leveled (APC = 0.01%, −0.17%, 0.18%) (Table 1 and Supplemental Table 2, http://links.lww.com/HC9/A501). Among adults (+ 20 y), crude global NAFLD-LRM rate per 100,000 increased by APC = + 1.18% (1.04%, 1.33%) from 2.94 to 3.27 (Table 2).

Most of the regions (19 out of 21 regions) experienced a worsening trend (>0%) in all-age crude NAFLD-LRM rate. A substantial increase (APC of ≥ + 2.0%) was observed in High-income North America, the Caribbean, East Asia, South Asia, and Tropical and Central Latin America. When the effects of population growth and aging were adjusted by converting counts to all-age age-standardized rates, High-income North American and Caribbean had still a substantially worsening trend (APC of ≥ + 1.5%), mainly driven by United States (APC = + 1.60%), Dominican Republic, Saint Vincent and the Grenadines, Saint Lucia, and Cuba (APC of ≥ + 2.00%) (Supplemental Tables 2 and 7, http://links.lww.com/HC9/A501, Supplemental Figure 2B, http://links.lww.com/HC9/A504).

At the country level, a worsening trend (≥ 0%) in all-age crude NAFLD-LRM rate was observed among 167 out of 204 countries, while this trend in the age-standardized death rate was observed among 97 countries (Supplemental Tables 7, http://links.lww.com/HC9/A501 and 8 and Figure 3B). Among countries with ≥ 20 million population size, Ukraine*, Nepal*, Morocco*, Burkina Faso*, Unites States*, Myanmar*, Vietnam*, and Algeria* have experienced an annual increase of ≥ + 1.0% in the age-standardized death rate.

#### LRM rates according to cirrhosis and liver cancer

**FIGURE 4:**
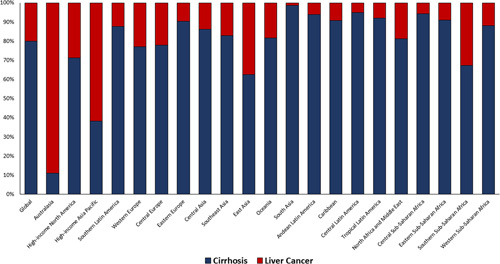
Contribution of cirrhosis and liver cancer for NAFLD-related liver mortality by region, 2019. Source Data: Global Burden of Disease 2019.

Globally in 2019, cirrhosis contributed 80.0% of NAFLD-LRM, while 20% was accounted for by liver cancer (Figure 4). On the other hand, liver cancer accounted for 88.9% of NAFLD-LRM in Australasia and 61.7% in High-income Asia Pacific regions.

**FIGURE 5:**
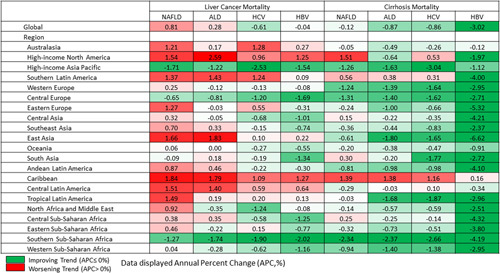
Annual percent chance in age-standardized mortality of liver cancer and cirrhosis by each liver disease from 2010 to 2019. Source Data: Global Burden of Disease 2019.

During this period, the all-age age-standardized global death related to liver cancer due to NAFLD increased faster than those of other liver diseases (NAFLD, APC = + 0.81% [0.67%, 0.95%] vs. ALD, + 0.28% [0.67%, 0.95%)]; HCV, − 0.61% [− 0.69%, − 0.54%]; and HBV, − 0.04% *p* = 0.685]) (Figure 5). A worsening trend (APC of ≥ 0%) in all-age age-standardized liver cancer death for NAFLD occurred in most of the regions (17 out 21 regions), while for ALD in 12 regions; for HCV in 8 regions; and for HBV in 6 regions. Furthermore, a decrease in the all-age age-standardized global death rate for cirrhosis due to NAFLD was slower than those of other liver diseases (NAFLD, APC = − 0.12% [− 0.29%, − 0.02%] vs. ALD, -0.87% [− 1.10%, − 0.86%)]; HCV, − 0.86% [− 0.98%, − 0.75%]; and HBV, − 3.02 [− 3.33%, − 2.70]). A worsening trend (APC of ≥ 0%) in all-age age-standardized cirrhosis death for NAFLD occurred in some regions (5 out 21 regions), while for ALD in 2 regions; for HCV in 4 regions; and for HBV in 1 region.

### Data quality

Since the GBD estimates lack primary data sources, data are also obtained from cancer registries, vital registration systems, or other similar sources. In this context, the GBD has developed a simple star-rating system from 0 to 5 to give an assessment of the quality of the data available in a given country over the entire time series used for their estimates. Data quality rating for the causes of death data is available in Supplemental Table 9, http://links.lww.com/HC9/A501.

## DISCUSSION

Over the past 3 decades, country-specific and meta-analytic data have provided estimates for the prevalence of NAFLD.^9–12,14^ In this context, the most recent data suggest that the global prevalence of NAFLD among adults is 30% and children is 7.4%.^9,14^ Despite being a powerful tool, meta-analytic assessment of the burden of NAFLD has some limitations, including significant heterogeneity that can potentially bias the data generated.^51,54,55^ An alternative is the use of other methodologies, such as that provided by GBD data sets. In this context, GBD not only can provide country-specific and region-specific prevalence and mortality data but will also allow the assessment of change in these rates over time.

Our current analysis suggests the global prevalence of NAFLD among adults increased over 3 decades from 17.6% (1990) to 23.4% (2019), with an average increase of 1.0% annually. The highest prevalence of NAFLD among adults was observed in the MENA region (2019 prevalence rate of 40.85%). These findings are not surprising as the prevalence of NAFLD appears to follow the trajectory of obesity and T2D, and both are highly prevalent diseases globally, especially in the MENA region.^9,10,13,14^. These increases in NAFLD burden were similarly experienced in men and women even though the actual rates were higher in males than females.^56–58^

We also investigated NAFLD prevalence by region. Not surprisingly, East and South Asia accounted for the highest growth, with China and India responsible for the most rapid growth. These findings confirm prior reports which pointed out that Asia is experiencing an explosive increase in NAFLD prevalence rates. In fact, the top 10 countries that experienced growth in NAFLD included Maldives, India, China, Nepal, Syria, Sudan, Oman, Saudi Arabia, Bangladesh, and Bhutan. The age-standardized prevalence rates were similar, except the top 10 countries included China, India, Nepal, Sudan, Nepal, Nauru, Turkmenistan, Japan, Botswana, Uzbekistan, and Oman. These differences can be explained by population growth, the aging trend seen in different countries, and changes in diet and lifestyles that now closely resemble those seen in Western countries.^3,4,8,59–62^ Nevertheless, these geographical disparities in the NAFLD prevalence highlight the importance of culturally appropriate interventions, so any policies should be developed in consult with local public health officials.^63^

In addition to the global prevalence rates, our study assessed NAFLD-LRM rates. Again, these rates increased globally as well as across most regions of the world. In this context, an acceleration in NAFLD-LRM was observed from 2010 to 2019 with an APC of + 1.18%. Major regional drivers of the increase included High-Income North America, East Asia, and the Caribbean, with each area experiencing an acceleration in deaths from 2010 to 2019 with an APC of + 2.5%. More specifically, the United States, Dominican Republic, Saint Vincent, the Grenadines, Saint Lucia, and Cuba were the main country drivers of this increase after adjusting for the effects of population growth and aging. These accelerations in NAFLD-related liver deaths also appear to be following the global burden of disease increases of T2D and obesity.^64^ Therefore, efforts must continue to focus on education about healthy lifestyle choices, which include diet, exercise, stopping/not smoking, avoiding alcohol, and the development of environments that provide healthy food choices, safe places to exercise, and healthy air to breathe.^65–70^

Unlike trends in crude NAFLD-LRM rates, global age-standardized NAFLD-LRM rates have been steady, with most countries showing a trend of improvement over the past decade. We suspect this pattern in age-standardized “NAFLD liver-related deaths” is likely not only due to changes in population growth and the aging of the population but also due to the methodology used by GBD to estimate liver deaths and the associated etiologies. GBD estimates overall liver deaths and then splits these deaths into 5 etiology-specific deaths according to a proportion model. Since etiological proportion data are limited, and overall liver deaths have been decreasing due to improved outcomes for HCV and HBV, a worsening trend in NAFLD-liver mortality can be masked and underestimated. Therefore, we suggest that improvement in viral hepatitis liver-related deaths rather than better treatment of NAFLD risk factors (eg, the treatment of T2D with GLP1 agonist) are the drivers of this improvement in age-standardized mortality rates for NAFLD.

In fact, given the scope of the GBD data, we also explored liver cancer and cirrhosis-related deaths for the major causes of liver disease [(NAFLD, HCV, HBV, and ALD] and found that from 2010 to 2019, the global age-standardized death rate from liver cancer due to NAFLD increased faster than those of other liver diseases (ALD, HCV, and HBV), a trend noted in 80% of the global regions. Similar patterns were noted for deaths from liver cirrhosis related to NAFLD compared with ALD, HBV, and HCV. Together these results validate 2 prior GBD reports and provide more evidence as to why NAFLD-related deaths may also be masked in the way LRM is reported.^71,72^ Noteworthy in this particular analysis is the large decrease in HBV LRM and, to some extent in HCV LRM, which is excellent news for public health and the World Health Organization’s goal of viral hepatitis elimination by 2030.^73^

Finally, we also assessed the burden of NAFLD only for the year 2019. Our data confirmed previously published findings that East and South Asia continued to have the highest burden of NAFLD. Furthermore, the MENA region is now ranked third for having the most prevalent cases and fifth for the highest number of deaths related to NAFLD. In this context, United Arab Emirates, Qatar, Bahrain, Kuwait, Saudi Arabia, Egypt, Turkey, Iran, Tunisia, and Libya had the highest NAFLD prevalence at ≥30.2%. In fact, Egypt was also one of the top 10 countries with the highest NAFLD-liver death rate (>6.0 per 100,000). The other 9 countries included Mongolia, Mexico, Egypt, Honduras, Moldova, Thailand, Puerto Rico, Virgin Islands, United States, Guatemala, and Bolivia.

There are strengths of this study. The most important strength of the current study is that we used the updated data from GBD estimates, which provide the only peer-reviewed estimates of cause-specific mortality available for each age, sex, year, and location throughout the world. These data also have limitations. The accuracy of the GBD estimates was limited by the quality and availability of each country’s vital registration system. For some locations without these data sources, GBD estimates heavily relied on the modeling process, predictive covariates, trends from past time, or trends from neighboring countries, resulting in some uncertainty, which potentially underestimates liver cancer mortality in low-income countries. GBD NAFLD prevalence estimates were lower than meta-analysis studies mainly because of GBD data sources and methodologies. However, we found a similar geographical pattern between GBD 2019 and a recent meta-analysis on the global prevalence of NAFLD, providing useful confirmation of estimates at regional and national levels.^9^ Since there are no available IHME publications that describe the GBD methodology for obtaining the incidence of NAFLD, we chose not to report NAFLD incidence at this time. In addition, since liver cancer and cirrhosis etiological proportion data are limited over time, temporal trends in etiological liver deaths are heavily influenced by those in the parent liver death estimates, so further study will be needed to validate or refute this methodology of determining the etiology of LRM.^74,75^

In summary, the use of GBD data has provided a unique opportunity to explore the burden of NAFLD on the global level using one source of data. Our data suggest the burden of NAFLD is high and increasing. In addition, Asia and MENA not only have the highest NAFLD prevalence rates but also the highest LRM rates. Our study supports other reports that NAFLD is becoming the most common cause of liver disease. These data have important implications for policymakers at the country level as well as the global level for the World Health Organization. Policymakers around the world need to understand the contributors of NAFLD within their countries and work together to develop culturally appropriate interventions aimed at reducing the risk factors of NAFLD. In addition, these data have important implications related to the current nomenclature debate about NAFLD. In this context, the future nomenclature of NAFLD and its associated coding must be carefully considered to not adversely affect the data collection for the future cycles of GBD.

## Supplementary Material

SUPPLEMENTARY MATERIAL
