# Supplementary material for: The burden of nonalcoholic fatty liver disease (NAFLD) is rapidly growing in every region of the world from 1990 to 2019
Source: Hepatol Commun. 2023 Oct 2;7(10):e0251. doi: 10.1097/HC9.0000000000000251 (PMC10545420; doi:10.1097/HC9.0000000000000251)
Supplement: SUPPLEMENTARY MATERIAL [file hc9-7-e0251-s001.docx]

Supplemental Table 1: CODem and Dismod-MR Covariates Used for Cirrhosis

| Covariates selected for CODEm model | Covariate selected for DisMod-MR model | | | |
| --- | --- | --- | --- | --- |
| Cirrhosis | Cirrhosis due to HBV | Cirrhosis due to HCV | Cirrhosis due to ALD | Cirrhosis due to NAFLD/NASH |
| Liters of alcohol per capita Seroprevalence (HBsAg) age-standardized  Seroprevalence (anti-HCV) age-standardized Hepatitis B vaccine coverage proportion, aged through time -  Mean BMI  Healthcare access and quality index Diabetes prevalence age-standardized  Schistosomiasis prevalence Intravenous drug use  Education (years per capita)  Lag distributed income (LDI)  Socio-demographic index | Seroprevalence (HBsAg) age standardized  Proportion of liver cancer due to hepatitis B (age-standardized)  Hepatitis B 3-dose coverage (proportion), lagged 10 years Proportion of cirrhosis due to alcohol  Proportion of cirrhosis due to hepatitis C Proportion of cirrhosis due to other causes Proportion of cirrhosis due to NASH | Seroprevalence (anti-HCV) age standardized  Proportion of liver cancer due to hepatitis C (Age Standardized)  Proportion of cirrhosis due to alcohol  Proportion of cirrhosis due to hepatitis B  Proportion of cirrhosis due to other causes  Proportion of cirrhosis due to NASH | Liters of alcohol consumed per capita Alcohol abstainer proportion, age-standardized  Proportion of liver cancer due to alcohol (Age Standardized)  Proportion of cirrhosis due to hepatitis B Proportion of cirrhosis due to hepatitis C  Proportion of cirrhosis due to other causes  Proportion of cirrhosis due to NASH | Mean BMI  Prevalence of obesity  NAFLD/NASH prevalence  Proportion of liver cancer due to NASH (Age Standardized)  Proportion of cirrhosis due to hepatitis B  Proportion of cirrhosis due to hepatitis C  Proportion of cirrhosis due to alcohol Proportion of cirrhosis due to other causes |
| Liver cancer | Liver cancer due to HBV | Liver cancer due to HCV | Liver cancer due to ALD | Liver cancer due to NAFLD/NASH |
| Alcohol (liters per capita) HIV age-standardized prevalence Hepatitis B (HBsAg) Seroprevalence Hepatitis C (IgG) Seroprevalence Log-transformed SEV scalar: Liver C Hepatitis B 3-dose coverage (proportion) Hepatitis B 3-dose coverage (proportion), lagged 5 years Hepatitis B 3-dose coverage (proportion), lagged 10 years Hepatitis B vaccine coverage (proportion), aged through time Intravenous drug use (age standardized proportion) Cumulative cigarettes (5 years)  Cumulative cigarettes (10 years)  Cumulative cigarettes (15 years)  Cumulative cigarettes (20 years)  Diabetes Age-Standardized Prevalence (proportion) Diabetes fasting plasma glucose (mmol/L) Mean BMI Tobacco (cigarettes per capita) Red meats adjusted (g) Healthcare access and quality index Education (years per capita) National income per capita ($ per capita) Socio-demographic Index | Alcohol (liters per capita) Hepatitis B (HBsAg) seroprevalence Hepatitis C (IgG) seroprevalence NAFLD/NASH prevalence Hepatitis B (HBsAg) seroprevalence Hepatitis B 3-dose coverage (proportion) | ` | Alcohol (liters per capita) Hepatitis B (HBsAg) seroprevalence Hepatitis C (IgG) seroprevalence NAFLD/NASH prevalence Alcohol abstainer proportion, age-standardized | Mean BMI Prevalence of obesity NAFLD/NASH prevalence |

| S Table 2. Among All-ages (Children and Adults), NAFLD Prevalent Cases, Age-standardized Prevalence Rate, NAFLD-related Liver Mortality, Age-standardized Mortality Rate, and Annual Percent Change in Age-standardized Rates During 1990-2019 and 2010-2019 | | | | | |
| --- | --- | --- | --- | --- | --- |
|  | Prevalent cases (Prevalence %) | | | APC (95% CI) | |
|  | 1990 | 2010 | 2019 | 1990-2019 | 2010-2019 |
| Global | 561,366,824 (12.07) | 958,781,491 (13.71) | 1,235,699,718 (15.02) | 0.77 (0.74 - 0.79) | 1.07 (1.01 - 1.12) |
| Sex |  |  |  |  |  |
| Males | 309,499,362 (13.42) | 522,832,002 (15.13) | 679,286,300 (16.79) | 0.76 (0.74 - 0.79) | 1.14 (1.10 - 1.18) |
| Females | 251,867,461 (10.75) | 435,949,489 (12.31) | 556,413,418 (13.28) | 0.78 (0.71 - 0.85) | 0.99 (0.81 - 1.17) |
| Region |  |  |  |  |  |
| Australasia | 1,624,919 (7.27) | 2,856,678 (9.07) | 3,460,100 (9.44) | 0.90 (0.87 - 0.94) | 0.41 (0.39 - 0.44) |
| High-income North America | 23,246,580 (7.26) | 36,801,272 (8.80) | 44,316,610 (9.40) | 0.89 (0.85 - 0.93) | 0.73 (0.64 - 0.82) |
| High-income Asia Pacific | 13,535,203 (6.84) | 18,679,483 (7.42) | 20,940,590 (7.67) | 0.33 (0.19 - 0.46) | 0.19 (-0.08 - 0.47) |
| Southern Latin America | 3,140,551 (6.62) | 5,433,572 (8.31) | 6,483,932 (8.60) | 0.91 (0.89 - 0.93) | 0.37 (0.35 - 0.39) |
| Western Europe | 37,026,088 (7.88) | 54,280,513 (9.68) | 59,006,466 (9.93) | 0.78 (0.75 - 0.82) | 0.24 (0.17 - 0.31) |
| Central Europe | 14,758,541 (10.68) | 17,645,673 (11.46) | 18,628,411 (11.90) | 0.37 (0.36 - 0.38) | 0.41 (0.40 - 0.42) |
| Eastern Europe | 28,742,280 (11.03) | 32,546,807 (11.85) | 34,111,363 (12.30) | 0.37 (0.37 - 0.38) | 0.41 (0.40 - 0.42) |
| Central Asia | 6,860,544 (12.30) | 10,106,415 (13.35) | 12,815,306 (14.15) | 0.48 (0.48 - 0.49) | 0.66 (0.64 - 0.67) |
| Southeast Asia | 56,613,903 (16.11) | 101,868,137 (17.50) | 128,069,888 (18.30) | 0.44 (0.43 - 0.45) | 0.50 (0.48 - 0.53) |
| East Asia | 138,889,890 (12.54) | 232,240,656 (13.70) | 303,125,668 (15.68) | 0.81 (0.62 - 1.00) | 1.86 (1.45 - 2.26) |
| Oceania | 702,208 (15.75) | 1,328,167 (16.70) | 1,744,123 (16.87) | 0.24 (0.23 - 0.25) | 0.13 (0.12 - 0.14) |
| South Asia | 95,443,036 (12.25) | 169,135,884 (12.85) | 241,842,616 (14.51) | 0.59 (0.52 - 0.66) | 1.37 (1.23 - 1.50) |
| Andean Latin America | 3,227,341 (11.76) | 6,378,816 (13.13) | 8,442,452 (13.69) | 0.52 (0.51 - 0.54) | 0.46 (0.45 - 0.47) |
| Caribbean | 4,341,266 (14.54) | 7,017,608 (15.83) | 8,176,331 (16.17) | 0.37 (0.36 - 0.38) | 0.24 (0.23 - 0.25) |
| Central Latin America | 17,059,656 (14.59) | 33,401,494 (15.99) | 42,166,980 (16.62) | 0.45 (0.44 - 0.46) | 0.42 (0.41 - 0.44) |
| Tropical Latin America | 15,654,055 (13.16) | 29,996,957 (14.60) | 37,986,673 (15.24) | 0.51 (0.49 - 0.52) | 0.48 (0.46 - 0.51) |
| North Africa and Middle East | 60,106,799 (24.42) | 123,303,691 (26.80) | 161,460,206 (27.75) | 0.45 (0.42 - 0.48) | 0.39 (0.31 - 0.47) |
| Central Sub-Saharan Africa | 4,246,738 (12.57) | 8,090,240 (12.98) | 11,344,041 (13.33) | 0.20 (0.20 - 0.21) | 0.30 (0.30 - 0.31) |
| Eastern Sub-Saharan Africa | 13,972,063 (12.56) | 25,837,299 (13.28) | 36,093,949 (13.71) | 0.30 (0.29 - 0.32) | 0.35 (0.32 - 0.39) |
| Southern Sub-Saharan Africa | 6,093,904 (16.00) | 10,487,515 (17.36) | 13,162,068 (18.08) | 0.42 (0.41 - 0.43) | 0.46 (0.44 - 0.47) |
| Western Sub-Saharan Africa | 16,081,260 (13.16) | 31,344,615 (14.05) | 42,321,947 (14.28) | 0.28 (0.27 - 0.29) | 0.18 (0.17 - 0.19) |
|  | **NAFLD-related Liver Mortality (Mortality Rate per 100,000)** | | | **APC (95% CI)** | |
|  | 1990 | 2010 | 2019 | 1990-2019 | 2010-2019 |
| Global | 93,758 (2.39) | 132,562 (2.09) | 168,969 (2.09) | -0.47 (-0.54 - -0.39) | 0.01 (-0.17 - 0.18) |
| Sex |  |  |  |  |  |
| Males | 48,688 (2.66) | 69,940 (2.35) | 89,763 (2.38) | -0.37 (-0.44 - -0.30) | 0.14 (0.05 - 0.23) |
| Females | 45,070 (2.15) | 62,622 (1.84) | 79,206 (1.82) | -0.56 (-0.63 - -0.49) | -0.10 (-0.28 - 0.08) |
| Region |  |  |  |  |  |
| Australasia | 259 (1.15) | 486 (1.27) | 637 (1.32) | 0.47 (0.23 - 0.70) | 0.40 (0.01 - 0.78) |
| High-income North America | 4,488 (1.32) | 7,113 (1.41) | 9,903 (1.65) | 0.79 (0.51 - 1.06) | 1.52 (0.91 - 2.13) |
| High-income Asia Pacific | 2,310 (1.18) | 3,508 (0.93) | 3,984 (0.82) | -1.25 (-1.38 - -1.12) | -1.54 (-1.82 - -1.27) |
| Southern Latin America | 936 (2.05) | 1,275 (1.86) | 1,630 (1.96) | -0.12 (-0.36 - 0.12) | 0.66 (0.28 - 1.03) |
| Western Europe | 10,607 (1.90) | 11,528 (1.49) | 12,359 (1.38) | -1.13 (-1.23 - -1.03) | -0.95 (-1.08 - -0.82) |
| Central Europe | 2,205 (1.53) | 2,684 (1.48) | 2,658 (1.32) | -0.52 (-0.70 - -0.34) | -1.20 (-1.54 - -0.86) |
| Eastern Europe | 2,977 (1.09) | 8,069 (2.76) | 8,174 (2.65) | 3.25 (2.30 - 4.20) | -0.66 (-2.11 - 0.81) |
| Central Asia | 969 (2.08) | 2,441 (4.12) | 3,069 (4.22) | 2.49 (2.24 - 2.74) | 0.22 (-0.11 - 0.56) |
| Southeast Asia | 10,733 (4.25) | 18,508 (4.23) | 23,704 (4.18) | -0.06 (-0.13 - 0.01) | -0.17 (-0.33 - -0.00) |
| East Asia | 21,510 (2.53) | 19,006 (1.29) | 25,413 (1.29) | -2.28 (-2.50 - -2.06) | 0.08 (-0.14 - 0.31) |
| Oceania | 47 (1.51) | 83 (1.47) | 107 (1.46) | -0.14 (-0.23 - -0.05) | -0.16 (-0.25 - -0.06) |
| South Asia | 11,572 (2.01) | 15,611 (1.57) | 21,896 (1.60) | -0.75 (-1.03 - -0.46) | 0.21 (-0.17 - 0.60) |
| Andean Latin America | 1,250 (6.10) | 2,469 (6.05) | 3,133 (5.68) | -0.25 (-0.52 - 0.02) | -0.64 (-0.93 - -0.35) |
| Caribbean | 1,057 (4.08) | 1,282 (3.02) | 1,773 (3.43) | -0.57 (-0.82 - -0.31) | 1.45 (0.77 - 2.12) |
| Central Latin America | 6,039 (6.96) | 11,121 (6.37) | 14,757 (6.24) | -0.37 (-0.63 - -0.10) | -0.20 (-0.65 - 0.26) |
| Tropical Latin America | 2,072 (2.25) | 3,622 (1.99) | 4,828 (2.00) | -0.39 (-0.78 - 0.00) | 0.10 (-0.74 - 0.95) |
| North Africa and Middle East | 6,019 (4.24) | 10,690 (3.86) | 14,476 (3.84) | -0.33 (-0.48 - -0.18) | -0.03 (-0.25 - 0.19) |
| Central Sub-Saharan Africa | 740 (3.32) | 1,127 (2.87) | 1,554 (2.89) | -0.44 (-0.66 - -0.21) | 0.25 (-0.31 - 0.82) |
| Eastern Sub-Saharan Africa | 3,541 (5.02) | 5,083 (4.50) | 6,726 (4.40) | -0.46 (-0.51 - -0.40) | -0.24 (-0.31 - -0.18) |
| Southern Sub-Saharan Africa | 700 (2.51) | 1,299 (2.94) | 1,322 (2.41) | -0.11 (-0.44 - 0.21) | -1.96 (-2.12 - -1.79) |
| Western Sub-Saharan Africa | 3,727 (4.49) | 5,557 (4.09) | 6,865 (3.80) | -0.56 (-0.70 - -0.43) | -0.81 (-0.91 - -0.72) |
| Abbreviations: CI, confidence interval; APC, Annual Percent Change Standardized to the World standard population developed for the GBD study | | | | | |

| Supplementary Table 3. Joinpoint Analysis of All-ages Crude NAFLD Prevalence Rate, Stratified by Region, 1990-2019 | | | |
| --- | --- | --- | --- |
| Region | Trend Segment | Years | APC (95% CI) |
| Global | 1 | 1990 - 2000 | 1.29 (1.26 - 1.32) |
| Global | 2 | 2000 - 2005 | 0.70 (0.59 - 0.81) |
| Global | 3 | 2005 - 2016 | 2.09 (2.07 - 2.12) |
| Global | 4 | 2016 - 2019 | 1.06 (0.90 - 1.22) |
| Australasia | 1 | 1990 - 1995 | 1.83 (1.76 - 1.90) |
| Australasia | 2 | 1995 - 2000 | 2.05 (1.96 - 2.14) |
| Australasia | 3 | 2000 - 2004 | 1.61 (1.47 - 1.75) |
| Australasia | 4 | 2004 - 2009 | 1.17 (1.08 - 1.26) |
| Australasia | 5 | 2009 - 2019 | 0.82 (0.80 - 0.84) |
| High-income North America | 1 | 1990 - 1995 | 0.97 (0.92 - 1.02) |
| High-income North America | 2 | 1995 - 1999 | 1.49 (1.37 - 1.60) |
| High-income North America | 3 | 1999 - 2010 | 1.39 (1.37 - 1.41) |
| High-income North America | 4 | 2010 - 2014 | 2.13 (2.03 - 2.24) |
| High-income North America | 5 | 2014 - 2019 | 0.82 (0.78 - 0.87) |
| High-income Asia Pacific | 1 | 1990 - 1995 | 0.99 (0.59 - 1.40) |
| High-income Asia Pacific | 2 | 1995 - 2000 | 0.27 (-0.26 - 0.79) |
| High-income Asia Pacific | 3 | 2000 - 2006 | 2.29 (1.94 - 2.64) |
| High-income Asia Pacific | 4 | 2006 - 2014 | 1.56 (1.36 - 1.77) |
| High-income Asia Pacific | 5 | 2014 - 2019 | 0.54 (0.18 - 0.90) |
| Southern Latin America | 1 | 1990 - 1994 | 1.71 (1.61 - 1.81) |
| Southern Latin America | 2 | 1994 - 2000 | 2.09 (2.02 - 2.16) |
| Southern Latin America | 3 | 2000 - 2005 | 1.75 (1.65 - 1.84) |
| Southern Latin America | 4 | 2005 - 2010 | 1.24 (1.14 - 1.33) |
| Southern Latin America | 5 | 2010 - 2019 | 0.96 (0.94 - 0.99) |
| Western Europe | 1 | 1990 - 1996 | 1.86 (1.77 - 1.96) |
| Western Europe | 2 | 1996 - 2005 | 1.50 (1.44 - 1.56) |
| Western Europe | 3 | 2005 - 2014 | 0.86 (0.81 - 0.92) |
| Western Europe | 4 | 2014 - 2019 | 0.26 (0.14 - 0.37) |
| Central Europe | 1 | 1990 - 1996 | 1.17 (1.15 - 1.18) |
| Central Europe | 2 | 1996 - 2002 | 1.23 (1.22 - 1.25) |
| Central Europe | 3 | 2002 - 2007 | 1.09 (1.07 - 1.12) |
| Central Europe | 4 | 2007 - 2014 | 0.99 (0.98 - 1.00) |
| Central Europe | 5 | 2014 - 2019 | 0.81 (0.79 - 0.83) |
| Eastern Europe | 1 | 1990 - 1996 | 0.85 (0.83 - 0.88) |
| Eastern Europe | 2 | 1996 - 2008 | 1.03 (1.02 - 1.04) |
| Eastern Europe | 3 | 2008 - 2013 | 0.85 (0.80 - 0.90) |
| Eastern Europe | 4 | 2013 - 2019 | 0.51 (0.48 - 0.53) |
| Central Asia | 1 | 1990 - 1996 | 0.53 (0.50 - 0.56) |
| Central Asia | 2 | 1996 - 2000 | 0.98 (0.89 - 1.07) |
| Central Asia | 3 | 2000 - 2004 | 1.20 (1.10 - 1.29) |
| Central Asia | 4 | 2004 - 2012 | 1.56 (1.53 - 1.59) |
| Central Asia | 5 | 2012 - 2019 | 1.19 (1.16 - 1.21) |
| Southeast Asia | 1 | 1990 - 2000 | 1.41 (1.40 - 1.42) |
| Southeast Asia | 2 | 2000 - 2005 | 1.71 (1.68 - 1.74) |
| Southeast Asia | 3 | 2005 - 2016 | 1.64 (1.63 - 1.65) |
| Southeast Asia | 4 | 2016 - 2019 | 1.57 (1.52 - 1.61) |
| East Asia | 1 | 1990 - 2000 | 1.68 (1.57 - 1.78) |
| East Asia | 2 | 2000 - 2005 | -0.81 (-1.20 - -0.41) |
| East Asia | 3 | 2005 - 2010 | 4.84 (4.44 - 5.25) |
| East Asia | 4 | 2010 - 2016 | 3.73 (3.46 - 4.00) |
| East Asia | 5 | 2016 - 2019 | 0.85 (0.28 - 1.42) |
| Oceania | 1 | 1990 - 1993 | 0.90 (0.84 - 0.95) |
| Oceania | 2 | 1993 - 1997 | 0.77 (0.71 - 0.83) |
| Oceania | 3 | 1997 - 2005 | 0.82 (0.80 - 0.83) |
| Oceania | 4 | 2005 - 2010 | 0.21 (0.18 - 0.25) |
| Oceania | 5 | 2010 - 2019 | 0.65 (0.64 - 0.66) |
| South Asia | 1 | 1990 - 2001 | 1.03 (0.97 - 1.09) |
| South Asia | 2 | 2001 - 2005 | 0.41 (-0.03 - 0.86) |
| South Asia | 3 | 2005 - 2010 | 1.54 (1.27 - 1.82) |
| South Asia | 4 | 2010 - 2015 | 3.17 (2.89 - 3.45) |
| South Asia | 5 | 2015 - 2019 | 1.87 (1.60 - 2.14) |
| Andean Latin America | 1 | 1990 - 1996 | 1.75 (1.72 - 1.77) |
| Andean Latin America | 2 | 1996 - 2000 | 1.89 (1.82 - 1.96) |
| Andean Latin America | 3 | 2000 - 2005 | 1.68 (1.63 - 1.72) |
| Andean Latin America | 4 | 2005 - 2009 | 1.49 (1.43 - 1.56) |
| Andean Latin America | 5 | 2009 - 2019 | 1.30 (1.29 - 1.31) |
| Caribbean | 1 | 1990 - 1994 | 1.30 (1.25 - 1.36) |
| Caribbean | 2 | 1994 - 2000 | 1.16 (1.12 - 1.19) |
| Caribbean | 3 | 2000 - 2005 | 1.65 (1.60 - 1.70) |
| Caribbean | 4 | 2005 - 2010 | 1.15 (1.10 - 1.20) |
| Caribbean | 5 | 2010 - 2019 | 0.94 (0.92 - 0.95) |
| Central Latin America | 1 | 1990 - 1993 | 1.91 (1.86 - 1.95) |
| Central Latin America | 2 | 1993 - 1999 | 1.78 (1.76 - 1.80) |
| Central Latin America | 3 | 1999 - 2006 | 1.55 (1.53 - 1.56) |
| Central Latin America | 4 | 2006 - 2015 | 1.74 (1.74 - 1.75) |
| Central Latin America | 5 | 2015 - 2019 | 1.49 (1.47 - 1.52) |
| Tropical Latin America | 1 | 1990 - 1994 | 1.96 (1.92 - 2.00) |
| Tropical Latin America | 2 | 1994 - 2004 | 1.83 (1.82 - 1.84) |
| Tropical Latin America | 3 | 2004 - 2010 | 1.68 (1.66 - 1.71) |
| Tropical Latin America | 4 | 2010 - 2014 | 1.89 (1.83 - 1.95) |
| Tropical Latin America | 5 | 2014 - 2019 | 1.45 (1.43 - 1.48) |
| North Africa and Middle East | 1 | 1990 - 1995 | 1.05 (0.97 - 1.13) |
| North Africa and Middle East | 2 | 1995 - 2000 | 1.48 (1.37 - 1.60) |
| North Africa and Middle East | 3 | 2000 - 2004 | 1.86 (1.69 - 2.04) |
| North Africa and Middle East | 4 | 2004 - 2009 | 1.71 (1.60 - 1.81) |
| North Africa and Middle East | 5 | 2009 - 2019 | 1.38 (1.36 - 1.41) |
| Central Sub-Saharan Africa | 1 | 1990 - 2001 | 0.19 (0.18 - 0.20) |
| Central Sub-Saharan Africa | 2 | 2001 - 2005 | 0.02 (-0.09 - 0.12) |
| Central Sub-Saharan Africa | 3 | 2005 - 2011 | 0.37 (0.32 - 0.41) |
| Central Sub-Saharan Africa | 4 | 2011 - 2015 | 0.68 (0.58 - 0.79) |
| Central Sub-Saharan Africa | 5 | 2015 - 2019 | 1.26 (1.20 - 1.33) |
| Eastern Sub-Saharan Africa | 1 | 1990 - 1997 | 0.13 (0.10 - 0.15) |
| Eastern Sub-Saharan Africa | 2 | 1997 - 2007 | 0.44 (0.42 - 0.46) |
| Eastern Sub-Saharan Africa | 3 | 2007 - 2011 | 0.71 (0.61 - 0.80) |
| Eastern Sub-Saharan Africa | 4 | 2011 - 2015 | 1.06 (0.96 - 1.15) |
| Eastern Sub-Saharan Africa | 5 | 2015 - 2019 | 1.35 (1.30 - 1.41) |
| Southern Sub-Saharan Africa | 1 | 1990 - 1995 | 1.32 (1.28 - 1.35) |
| Southern Sub-Saharan Africa | 2 | 1995 - 2006 | 1.14 (1.13 - 1.15) |
| Southern Sub-Saharan Africa | 3 | 2006 - 2010 | 1.28 (1.21 - 1.36) |
| Southern Sub-Saharan Africa | 4 | 2010 - 2014 | 1.56 (1.49 - 1.63) |
| Southern Sub-Saharan Africa | 5 | 2014 - 2019 | 1.28 (1.24 - 1.31) |
| Western Sub-Saharan Africa | 1 | 1990 - 1998 | 0.57 (0.55 - 0.58) |
| Western Sub-Saharan Africa | 2 | 1998 - 2004 | 0.33 (0.30 - 0.36) |
| Western Sub-Saharan Africa | 3 | 2004 - 2011 | -0.07 (-0.09 - -0.05) |
| Western Sub-Saharan Africa | 4 | 2011 - 2015 | 0.18 (0.11 - 0.24) |
| Western Sub-Saharan Africa | 5 | 2015 - 2019 | 0.92 (0.88 - 0.96) |
| Abbreviations: CI, confidence interval; APC, Annual Percent Change Trend Segment were detected by Joinpoint Regression Model | | | |

| S Table 4. Among All ages (Children and Adults), NAFLD Prevalent Cases, Crude Prevalence Rate, and Annual Percent Change in Crude Rate During 1990-2019 and 2010-2019, By 204 Countries and Territories | | | | | | |
| --- | --- | --- | --- | --- | --- | --- |
|  |  | Prevalent cases (Prevalence %) | | | Average APC (95% CI) | |
| Region | Country | 1990 | 2010 | 2019 | 1990-2019 | 2010-2019 |
| Australasia | Australia | 1,365,098 (8.10) | 2,422,803 (11.22) | 2,959,992 (12.05) | 1.38 (1.35 - 1.41) | 0.77 (0.75 - 0.79) |
| Australasia | New Zealand | 259,821 (7.60) | 433,875 (10.16) | 500,107 (11.12) | 1.32 (1.30 - 1.35) | 1.00 (0.95 - 1.05) |
| High-income North America | Canada | 1,830,124 (6.71) | 2,875,744 (8.56) | 3,391,406 (9.29) | 1.11 (1.08 - 1.14) | 0.88 (0.81 - 0.95) |
| High-income North America | United States | 21,412,780 (8.44) | 33,920,854 (10.98) | 40,920,076 (12.48) | 1.36 (1.33 - 1.39) | 1.46 (1.37 - 1.55) |
| High-income North America | Greenland | 3,145 (5.66) | 3,992 (7.05) | 4,424 (7.87) | 1.14 (1.11 - 1.17) | 1.23 (1.18 - 1.27) |
| High-income Asia Pacific | Brunei | 14,384 (5.56) | 29,656 (7.55) | 37,775 (8.64) | 1.52 (1.50 - 1.54) | 1.50 (1.48 - 1.52) |
| High-income Asia Pacific | Japan | 10,370,150 (8.24) | 12,831,976 (9.87) | 14,371,841 (11.25) | 1.09 (1.04 - 1.13) | 1.46 (1.37 - 1.55) |
| High-income Asia Pacific | South Korea | 2,915,757 (6.58) | 5,283,046 (10.71) | 5,852,771 (10.96) | 1.64 (1.17 - 2.10) | -0.12 (-0.85 - 0.62) |
| High-income Asia Pacific | Singapore | 234,912 (7.71) | 534,805 (10.53) | 678,203 (11.97) | 1.52 (1.50 - 1.55) | 1.43 (1.40 - 1.46) |
| Southern Latin America | Argentina | 1,974,336 (5.96) | 3,325,753 (8.09) | 3,977,457 (8.82) | 1.36 (1.33 - 1.38) | 0.94 (0.93 - 0.96) |
| Southern Latin America | Chile | 864,001 (6.51) | 1,685,406 (10.11) | 2,037,159 (11.19) | 1.88 (1.84 - 1.92) | 1.11 (1.07 - 1.15) |
| Southern Latin America | Uruguay | 302,087 (9.62) | 422,155 (12.55) | 468,990 (13.65) | 1.21 (1.19 - 1.24) | 0.92 (0.87 - 0.98) |
| Western Europe | Andorra | 4,731 (8.75) | 10,330 (12.33) | 11,061 (13.32) | 1.45 (1.37 - 1.54) | 0.92 (0.73 - 1.11) |
| Western Europe | Austria | 656,609 (8.45) | 958,783 (11.45) | 1,091,219 (12.24) | 1.28 (1.24 - 1.31) | 0.73 (0.68 - 0.79) |
| Western Europe | Belgium | 850,966 (8.53) | 1,202,838 (11.06) | 1,329,730 (11.64) | 1.08 (1.04 - 1.11) | 0.55 (0.53 - 0.58) |
| Western Europe | Cyprus | 58,602 (7.53) | 120,425 (10.74) | 153,099 (11.66) | 1.51 (1.49 - 1.53) | 0.90 (0.87 - 0.93) |
| Western Europe | Denmark | 387,017 (7.52) | 533,473 (9.64) | 590,321 (10.17) | 1.04 (1.02 - 1.06) | 0.59 (0.57 - 0.61) |
| Western Europe | Finland | 335,936 (6.71) | 497,506 (9.24) | 548,703 (9.91) | 1.36 (1.33 - 1.40) | 0.80 (0.75 - 0.85) |
| Western Europe | France | 4,366,743 (7.56) | 6,358,804 (9.97) | 7,060,640 (10.66) | 1.19 (1.16 - 1.22) | 0.73 (0.68 - 0.77) |
| Western Europe | Germany | 6,162,111 (7.71) | 8,694,195 (10.65) | 9,492,808 (11.18) | 1.28 (1.26 - 1.30) | 0.54 (0.49 - 0.59) |
| Western Europe | Greece | 968,012 (9.32) | 1,419,451 (12.81) | 1,394,277 (13.49) | 1.28 (1.26 - 1.30) | 0.56 (0.54 - 0.59) |
| Western Europe | Iceland | 19,194 (7.56) | 33,124 (10.39) | 38,942 (11.29) | 1.39 (1.38 - 1.41) | 0.91 (0.89 - 0.93) |
| Western Europe | Ireland | 314,726 (8.74) | 560,899 (12.19) | 657,168 (13.38) | 1.48 (1.46 - 1.50) | 1.02 (0.98 - 1.07) |
| Western Europe | Israel | 554,627 (11.18) | 1,217,786 (15.52) | 1,547,067 (16.62) | 1.37 (1.35 - 1.39) | 0.75 (0.72 - 0.78) |
| Western Europe | Italy | 8,677,703 (15.28) | 12,317,463 (20.42) | 12,593,986 (20.88) | 1.01 (0.90 - 1.11) | 0.03 (-0.24 - 0.30) |
| Western Europe | Luxembourg | 33,675 (8.83) | 54,263 (10.79) | 71,313 (11.53) | 0.92 (0.90 - 0.94) | 0.74 (0.69 - 0.79) |
| Western Europe | Malta | 30,296 (8.17) | 53,434 (12.60) | 59,918 (13.64) | 1.78 (1.75 - 1.80) | 0.86 (0.84 - 0.88) |
| Western Europe | Netherlands | 1,321,225 (8.85) | 1,850,819 (11.15) | 2,071,519 (12.07) | 1.07 (1.06 - 1.08) | 0.87 (0.84 - 0.90) |
| Western Europe | Norway | 340,484 (8.02) | 484,764 (9.96) | 569,498 (10.65) | 0.98 (0.96 - 0.99) | 0.72 (0.71 - 0.73) |
| Western Europe | Portugal | 1,111,905 (10.97) | 1,715,232 (15.89) | 1,836,905 (17.25) | 1.56 (1.53 - 1.59) | 0.90 (0.86 - 0.93) |
| Western Europe | Spain | 4,180,355 (10.78) | 6,829,136 (14.54) | 7,348,821 (15.97) | 1.36 (1.32 - 1.39) | 1.04 (0.97 - 1.12) |
| Western Europe | Sweden | 777,117 (9.05) | 1,065,850 (11.31) | 1,213,633 (11.87) | 0.94 (0.92 - 0.96) | 0.53 (0.51 - 0.55) |
| Western Europe | Switzerland | 558,874 (8.14) | 811,459 (10.19) | 963,866 (10.98) | 1.04 (1.03 - 1.04) | 0.82 (0.80 - 0.84) |
| Western Europe | United Kingdom | 5,278,485 (9.18) | 7,434,353 (11.69) | 8,300,436 (12.35) | 1.02 (1.00 - 1.04) | 0.59 (0.56 - 0.62) |
| Western Europe | Monaco | 3,759 (12.35) | 5,302 (14.84) | 5,816 (15.48) | 0.78 (0.76 - 0.80) | 0.46 (0.45 - 0.47) |
| Western Europe | San Marino | 2,194 (9.31) | 3,800 (12.24) | 4,278 (12.93) | 1.13 (1.11 - 1.15) | 0.61 (0.58 - 0.63) |
| Central Europe | Albania | 420,174 (12.69) | 529,096 (18.32) | 584,546 (21.49) | 1.84 (1.79 - 1.89) | 1.81 (1.75 - 1.86) |
| Central Europe | Bosnia and Herzegovina | 763,914 (16.83) | 886,692 (23.51) | 850,731 (25.78) | 1.50 (1.46 - 1.55) | 1.06 (1.01 - 1.11) |
| Central Europe | Bulgaria | 1,151,676 (13.27) | 1,125,753 (15.13) | 1,113,143 (16.05) | 0.66 (0.65 - 0.67) | 0.65 (0.64 - 0.66) |
| Central Europe | Croatia | 665,800 (13.59) | 732,369 (16.75) | 753,562 (17.74) | 0.92 (0.90 - 0.94) | 0.63 (0.61 - 0.65) |
| Central Europe | Czech Republic | 1,213,858 (11.79) | 1,519,532 (14.51) | 1,631,958 (15.33) | 0.91 (0.90 - 0.92) | 0.62 (0.60 - 0.64) |
| Central Europe | Hungary | 1,346,429 (12.96) | 1,577,410 (15.83) | 1,633,702 (16.89) | 0.91 (0.89 - 0.93) | 0.71 (0.69 - 0.74) |
| Central Europe | Macedonia | 238,853 (11.85) | 327,428 (15.47) | 365,157 (16.96) | 1.23 (1.18 - 1.29) | 1.04 (0.94 - 1.14) |
| Central Europe | Montenegro | 77,148 (12.33) | 95,178 (15.09) | 101,666 (16.39) | 0.99 (0.98 - 0.99) | 0.92 (0.90 - 0.93) |
| Central Europe | Poland | 4,115,068 (10.78) | 5,216,734 (13.60) | 5,746,331 (14.95) | 1.13 (1.13 - 1.14) | 1.07 (1.04 - 1.09) |
| Central Europe | Romania | 2,764,983 (11.82) | 3,225,618 (15.58) | 3,295,031 (17.13) | 1.29 (1.27 - 1.30) | 1.04 (1.02 - 1.07) |
| Central Europe | Serbia | 1,225,638 (13.04) | 1,388,721 (15.44) | 1,439,997 (16.46) | 0.81 (0.75 - 0.87) | 0.72 (0.66 - 0.78) |
| Central Europe | Slovakia | 520,793 (9.86) | 673,507 (12.45) | 740,952 (13.63) | 1.13 (1.10 - 1.15) | 1.00 (0.95 - 1.06) |
| Central Europe | Slovenia | 254,207 (12.90) | 347,636 (17.04) | 371,635 (17.92) | 1.14 (1.12 - 1.16) | 0.56 (0.53 - 0.59) |
| Eastern Europe | Belarus | 1,180,628 (11.28) | 1,312,536 (13.56) | 1,380,370 (14.53) | 0.87 (0.86 - 0.89) | 0.75 (0.73 - 0.78) |
| Eastern Europe | Estonia | 176,816 (11.27) | 187,159 (14.05) | 196,267 (14.96) | 0.99 (0.96 - 1.01) | 0.69 (0.64 - 0.75) |
| Eastern Europe | Latvia | 313,096 (11.78) | 308,206 (14.57) | 296,954 (15.50) | 0.95 (0.94 - 0.97) | 0.70 (0.68 - 0.72) |
| Eastern Europe | Lithuania | 418,533 (11.39) | 447,731 (14.49) | 434,632 (15.55) | 1.08 (1.04 - 1.12) | 0.77 (0.74 - 0.80) |
| Eastern Europe | Moldova | 458,592 (10.31) | 522,526 (13.49) | 570,595 (15.47) | 1.42 (1.39 - 1.44) | 1.53 (1.50 - 1.56) |
| Eastern Europe | Russian Federation | 19,075,852 (12.63) | 22,504,527 (15.48) | 23,977,534 (16.34) | 0.89 (0.87 - 0.91) | 0.61 (0.58 - 0.63) |
| Eastern Europe | Ukraine | 7,118,763 (13.52) | 7,264,122 (15.70) | 7,255,010 (16.47) | 0.68 (0.66 - 0.70) | 0.51 (0.47 - 0.56) |
| Central Asia | Armenia | 393,984 (11.54) | 510,452 (16.43) | 547,959 (18.15) | 1.58 (1.56 - 1.59) | 1.11 (1.08 - 1.14) |
| Central Asia | Azerbaijan | 847,725 (11.56) | 1,384,074 (14.91) | 1,768,073 (17.20) | 1.38 (1.36 - 1.40) | 1.61 (1.59 - 1.63) |
| Central Asia | Georgia | 727,972 (13.22) | 629,380 (15.80) | 616,670 (16.83) | 0.84 (0.82 - 0.85) | 0.69 (0.66 - 0.72) |
| Central Asia | Kazakhstan | 1,709,857 (10.45) | 2,037,684 (12.57) | 2,448,967 (13.32) | 0.83 (0.81 - 0.86) | 0.61 (0.59 - 0.63) |
| Central Asia | Kyrgyzstan | 478,891 (10.73) | 685,456 (12.19) | 858,945 (13.14) | 0.70 (0.69 - 0.71) | 0.83 (0.80 - 0.86) |
| Central Asia | Mongolia | 171,552 (7.97) | 299,199 (10.57) | 402,490 (11.88) | 1.39 (1.37 - 1.42) | 1.29 (1.24 - 1.34) |
| Central Asia | Tajikistan | 399,778 (7.44) | 657,081 (8.46) | 928,176 (9.78) | 0.95 (0.93 - 0.97) | 1.63 (1.59 - 1.67) |
| Central Asia | Turkmenistan | 321,573 (8.68) | 560,080 (12.22) | 727,381 (14.31) | 1.74 (1.70 - 1.79) | 1.76 (1.67 - 1.85) |
| Central Asia | Uzbekistan | 1,809,210 (8.64) | 3,343,009 (11.47) | 4,516,647 (13.41) | 1.54 (1.51 - 1.57) | 1.78 (1.75 - 1.81) |
| Southeast Asia | Cambodia | 1,010,073 (9.74) | 1,897,659 (13.05) | 2,545,969 (15.33) | 1.59 (1.57 - 1.61) | 1.81 (1.77 - 1.85) |
| Southeast Asia | Indonesia | 25,705,107 (13.87) | 45,285,728 (18.81) | 56,324,162 (21.71) | 1.56 (1.55 - 1.57) | 1.62 (1.60 - 1.63) |
| Southeast Asia | Laos | 337,543 (8.13) | 613,144 (9.65) | 815,319 (11.39) | 1.18 (1.16 - 1.21) | 1.89 (1.87 - 1.92) |
| Southeast Asia | Malaysia | 2,532,217 (14.34) | 5,483,840 (19.49) | 7,067,747 (22.58) | 1.59 (1.57 - 1.60) | 1.66 (1.64 - 1.68) |
| Southeast Asia | Maldives | 20,255 (9.13) | 52,525 (14.65) | 95,083 (19.08) | 2.59 (2.54 - 2.65) | 3.03 (2.98 - 3.08) |
| Southeast Asia | Myanmar | 4,935,223 (12.01) | 7,881,166 (15.56) | 9,732,642 (17.80) | 1.37 (1.36 - 1.38) | 1.52 (1.51 - 1.53) |
| Southeast Asia | Philippines | 5,553,549 (8.77) | 10,771,201 (11.15) | 14,059,848 (12.54) | 1.24 (1.23 - 1.26) | 1.32 (1.30 - 1.34) |
| Southeast Asia | Sri Lanka | 2,212,000 (12.84) | 3,429,288 (16.73) | 4,189,860 (19.17) | 1.41 (1.35 - 1.47) | 1.57 (1.44 - 1.70) |
| Southeast Asia | Thailand | 7,349,703 (12.92) | 13,708,605 (20.26) | 16,983,772 (24.22) | 2.19 (2.14 - 2.25) | 2.06 (1.97 - 2.14) |
| Southeast Asia | Timor-Leste | 74,546 (9.52) | 117,061 (10.59) | 151,893 (11.38) | 0.63 (0.60 - 0.66) | 0.83 (0.80 - 0.87) |
| Southeast Asia | Vietnam | 6,669,624 (9.82) | 12,257,864 (13.70) | 15,662,604 (16.25) | 1.76 (1.75 - 1.77) | 1.92 (1.90 - 1.95) |
| Southeast Asia | Mauritius | 127,742 (11.61) | 212,980 (16.79) | 249,469 (19.54) | 1.81 (1.81 - 1.82) | 1.70 (1.69 - 1.72) |
| Southeast Asia | Seychelles | 11,069 (15.16) | 19,034 (20.41) | 23,736 (23.24) | 1.47 (1.43 - 1.52) | 1.47 (1.37 - 1.56) |
| East Asia | China | 133951892 (11.32) | 224013997 (16.42) | 293422204 (20.63) | 2.13 (2.00 - 2.26) | 2.80 (2.55 - 3.06) |
| East Asia | North Korea | 2,268,955 (10.78) | 3,579,799 (14.07) | 4,269,361 (16.27) | 1.43 (1.41 - 1.45) | 1.63 (1.59 - 1.67) |
| East Asia | Taiwan | 2,669,043 (13.08) | 4,646,860 (20.04) | 5,434,103 (23.01) | 1.99 (1.93 - 2.05) | 1.53 (1.40 - 1.66) |
| Oceania | Fiji | 101,916 (13.42) | 164,040 (18.75) | 185,650 (20.37) | 1.45 (1.44 - 1.47) | 0.92 (0.91 - 0.94) |
| Oceania | Kiribati | 9,004 (12.16) | 15,303 (14.51) | 18,047 (15.21) | 0.77 (0.73 - 0.81) | 0.56 (0.47 - 0.66) |
| Oceania | Marshall Islands | 3,970 (8.68) | 6,937 (12.69) | 8,441 (14.85) | 1.88 (1.84 - 1.92) | 1.78 (1.69 - 1.87) |
| Oceania | Federated States of Micronesia | 11,240 (10.78) | 15,903 (15.07) | 17,102 (16.75) | 1.52 (1.47 - 1.57) | 1.19 (1.08 - 1.29) |
| Oceania | Papua New Guinea | 413,697 (10.12) | 848,184 (11.19) | 1,186,420 (12.02) | 0.60 (0.58 - 0.62) | 0.80 (0.79 - 0.82) |
| Oceania | Samoa | 20,846 (12.74) | 29,067 (15.70) | 33,966 (16.07) | 0.80 (0.78 - 0.82) | 0.26 (0.22 - 0.31) |
| Oceania | Solomon Islands | 31,541 (9.26) | 64,808 (11.68) | 81,638 (12.45) | 1.03 (1.00 - 1.06) | 0.74 (0.68 - 0.81) |
| Oceania | Tonga | 12,184 (12.59) | 16,804 (15.83) | 17,380 (16.98) | 1.02 (0.97 - 1.08) | 0.82 (0.68 - 0.95) |
| Oceania | Vanuatu | 16,343 (10.80) | 32,514 (13.22) | 42,052 (14.28) | 0.97 (0.96 - 0.98) | 0.86 (0.85 - 0.87) |
| Oceania | American Samoa | 7,166 (14.80) | 10,981 (19.23) | 11,677 (21.04) | 1.22 (1.19 - 1.24) | 1.05 (1.02 - 1.08) |
| Oceania | Cook Islands | 2,809 (14.77) | 4,140 (22.14) | 4,534 (25.21) | 1.88 (1.78 - 1.98) | 1.46 (1.42 - 1.51) |
| Oceania | Guam | 19,927 (14.57) | 31,350 (19.08) | 35,294 (20.68) | 1.22 (1.20 - 1.23) | 0.90 (0.87 - 0.92) |
| Oceania | Nauru | 1,174 (11.45) | 1,296 (12.46) | 1,488 (14.10) | 0.73 (0.69 - 0.76) | 1.47 (1.38 - 1.56) |
| Oceania | Niue | 358 (15.40) | 353 (21.92) | 390 (23.32) | 1.44 (1.42 - 1.46) | 0.67 (0.64 - 0.70) |
| Oceania | Northern Mariana Islands | 7,384 (16.26) | 12,108 (22.20) | 10,766 (25.33) | 1.52 (1.47 - 1.57) | 1.48 (1.37 - 1.60) |
| Oceania | Palau | 2,342 (15.20) | 4,116 (22.29) | 4,646 (25.80) | 1.84 (1.80 - 1.87) | 1.71 (1.67 - 1.74) |
| Oceania | Tokelau | 211 (12.48) | 203 (16.25) | 252 (17.86) | 1.24 (1.18 - 1.31) | 1.04 (0.95 - 1.12) |
| Oceania | Tuvalu | 1,239 (13.25) | 1,694 (15.85) | 2,004 (16.98) | 0.85 (0.83 - 0.88) | 0.80 (0.74 - 0.85) |
| South Asia | Bangladesh | 8,426,250 (7.73) | 16,350,935 (11.31) | 22,317,625 (14.01) | 2.08 (2.06 - 2.09) | 2.41 (2.39 - 2.42) |
| South Asia | Bhutan | 50,748 (8.29) | 91,675 (12.53) | 116,651 (15.47) | 2.15 (2.10 - 2.20) | 2.34 (2.28 - 2.40) |
| South Asia | India | 75,075,584 (8.77) | 130227795 (10.58) | 188363474 (13.54) | 1.52 (1.45 - 1.59) | 2.81 (2.66 - 2.96) |
| South Asia | Nepal | 1,509,370 (7.73) | 2,842,845 (10.27) | 3,947,139 (12.98) | 1.82 (1.78 - 1.85) | 2.68 (2.60 - 2.76) |
| South Asia | Pakistan | 10,381,084 (9.20) | 19,622,634 (10.72) | 27,097,727 (12.09) | 0.94 (0.90 - 0.98) | 1.30 (1.26 - 1.34) |
| Andean Latin America | Bolivia | 501,359 (7.81) | 1,049,016 (10.31) | 1,372,503 (11.43) | 1.33 (1.31 - 1.35) | 1.18 (1.13 - 1.23) |
| Andean Latin America | Ecuador | 1,331,828 (13.28) | 2,642,551 (17.69) | 3,491,265 (19.85) | 1.40 (1.36 - 1.43) | 1.32 (1.28 - 1.35) |
| Andean Latin America | Peru | 1,394,154 (6.42) | 2,687,249 (9.29) | 3,578,684 (10.53) | 1.72 (1.69 - 1.76) | 1.35 (1.27 - 1.43) |
| Caribbean | Antigua and Barbuda | 7,443 (12.26) | 13,777 (16.01) | 16,243 (18.36) | 1.40 (1.39 - 1.41) | 1.54 (1.53 - 1.54) |
| Caribbean | The Bahamas | 31,956 (12.46) | 58,705 (16.60) | 70,206 (18.63) | 1.39 (1.36 - 1.41) | 1.28 (1.25 - 1.30) |
| Caribbean | Barbados | 42,544 (16.76) | 59,660 (21.20) | 69,437 (23.32) | 1.15 (1.12 - 1.17) | 1.07 (1.05 - 1.08) |
| Caribbean | Belize | 18,953 (10.20) | 48,387 (14.72) | 66,908 (16.32) | 1.63 (1.59 - 1.67) | 1.12 (1.07 - 1.16) |
| Caribbean | Cuba | 1,621,098 (14.97) | 2,349,773 (20.49) | 2,580,954 (22.72) | 1.45 (1.43 - 1.48) | 1.16 (1.15 - 1.18) |
| Caribbean | Dominica | 9,537 (12.89) | 12,120 (17.39) | 13,212 (19.24) | 1.40 (1.37 - 1.42) | 1.14 (1.12 - 1.16) |
| Caribbean | Dominican Republic | 627,747 (8.71) | 1,251,801 (12.75) | 1,555,728 (14.30) | 1.72 (1.69 - 1.75) | 1.28 (1.26 - 1.30) |
| Caribbean | Grenada | 9,430 (11.01) | 16,664 (15.66) | 18,332 (17.76) | 1.67 (1.65 - 1.68) | 1.41 (1.39 - 1.43) |
| Caribbean | Guyana | 79,244 (10.29) | 105,155 (14.07) | 121,651 (15.78) | 1.47 (1.45 - 1.50) | 1.25 (1.21 - 1.29) |
| Caribbean | Haiti | 559,861 (8.81) | 1,021,466 (9.89) | 1,360,875 (10.97) | 0.76 (0.75 - 0.77) | 1.16 (1.13 - 1.19) |
| Caribbean | Jamaica | 287,703 (12.17) | 466,896 (16.95) | 533,895 (18.99) | 1.54 (1.53 - 1.56) | 1.26 (1.25 - 1.27) |
| Caribbean | Saint Lucia | 14,185 (10.32) | 27,934 (16.56) | 34,128 (19.54) | 2.22 (2.21 - 2.24) | 1.85 (1.83 - 1.87) |
| Caribbean | Saint Vincent and the Grenadines | 11,206 (10.18) | 17,879 (16.03) | 20,903 (18.47) | 2.08 (2.06 - 2.10) | 1.60 (1.58 - 1.62) |
| Caribbean | Suriname | 45,872 (11.87) | 85,805 (15.92) | 103,625 (17.99) | 1.44 (1.43 - 1.46) | 1.37 (1.34 - 1.40) |
| Caribbean | Trinidad and Tobago | 159,590 (13.27) | 259,601 (19.27) | 303,550 (21.88) | 1.75 (1.72 - 1.77) | 1.41 (1.36 - 1.47) |
| Caribbean | Bermuda | 11,612 (19.53) | 16,141 (24.66) | 17,277 (26.98) | 1.12 (1.11 - 1.13) | 1.01 (0.99 - 1.02) |
| Caribbean | Puerto Rico | 637,743 (17.65) | 933,937 (24.59) | 976,434 (27.73) | 1.57 (1.54 - 1.61) | 1.38 (1.33 - 1.43) |
| Caribbean | Saint Kitts and Nevis | 5,278 (12.76) | 9,285 (17.13) | 11,836 (19.89) | 1.54 (1.52 - 1.55) | 1.66 (1.65 - 1.67) |
| Caribbean | Virgin Islands, U.S. | 15,646 (14.76) | 23,568 (21.75) | 24,164 (23.24) | 1.58 (1.56 - 1.60) | 0.75 (0.72 - 0.78) |
| Central Latin America | Colombia | 2,776,687 (8.53) | 5,416,908 (12.12) | 6,886,762 (14.41) | 1.82 (1.80 - 1.84) | 1.93 (1.89 - 1.98) |
| Central Latin America | Costa Rica | 265,870 (8.75) | 535,394 (12.20) | 661,290 (14.02) | 1.64 (1.64 - 1.65) | 1.56 (1.55 - 1.57) |
| Central Latin America | El Salvador | 508,761 (9.66) | 835,517 (13.91) | 982,775 (15.71) | 1.70 (1.68 - 1.71) | 1.35 (1.34 - 1.36) |
| Central Latin America | Guatemala | 833,380 (10.46) | 1,913,627 (13.16) | 2,727,663 (15.34) | 1.32 (1.31 - 1.34) | 1.71 (1.67 - 1.75) |
| Central Latin America | Honduras | 485,133 (10.30) | 1,100,113 (13.77) | 1,517,827 (15.47) | 1.41 (1.39 - 1.42) | 1.29 (1.27 - 1.30) |
| Central Latin America | Mexico | 9,491,947 (11.10) | 18,019,194 (15.75) | 22,589,109 (18.08) | 1.70 (1.69 - 1.71) | 1.55 (1.54 - 1.56) |
| Central Latin America | Nicaragua | 328,139 (8.44) | 717,986 (12.53) | 951,735 (14.62) | 1.92 (1.90 - 1.94) | 1.73 (1.70 - 1.75) |
| Central Latin America | Panama | 266,886 (11.17) | 528,502 (15.12) | 700,779 (16.84) | 1.42 (1.41 - 1.44) | 1.19 (1.18 - 1.20) |
| Central Latin America | Venezuela | 2,102,852 (11.17) | 4,334,253 (15.59) | 5,149,040 (18.34) | 1.76 (1.69 - 1.82) | 1.94 (1.90 - 1.99) |
| Tropical Latin America | Brazil | 15,327,546 (10.30) | 29,352,411 (14.80) | 37,141,599 (17.14) | 1.77 (1.76 - 1.78) | 1.65 (1.63 - 1.68) |
| Tropical Latin America | Paraguay | 326,509 (8.07) | 644,545 (10.57) | 845,074 (12.19) | 1.44 (1.42 - 1.46) | 1.60 (1.58 - 1.62) |
| North Africa and Middle East | Algeria | 3,970,015 (15.70) | 8,350,534 (23.04) | 11,118,713 (26.57) | 1.84 (1.81 - 1.86) | 1.57 (1.53 - 1.62) |
| North Africa and Middle East | Bahrain | 116,544 (22.94) | 385,793 (30.91) | 535,817 (37.14) | 1.68 (1.64 - 1.71) | 2.09 (2.05 - 2.12) |
| North Africa and Middle East | Egypt | 13,421,484 (24.10) | 24,441,821 (29.18) | 30,467,230 (30.75) | 0.86 (0.83 - 0.89) | 0.64 (0.58 - 0.70) |
| North Africa and Middle East | Iran | 9,354,195 (15.98) | 21,386,052 (27.93) | 25,559,769 (30.32) | 2.23 (2.13 - 2.33) | 0.73 (0.54 - 0.93) |
| North Africa and Middle East | Iraq | 2,836,669 (16.12) | 6,441,938 (18.95) | 9,519,937 (22.60) | 1.18 (1.13 - 1.23) | 1.98 (1.87 - 2.10) |
| North Africa and Middle East | Jordan | 594,795 (15.76) | 1,615,982 (22.20) | 2,999,784 (25.78) | 1.72 (1.70 - 1.73) | 1.69 (1.67 - 1.72) |
| North Africa and Middle East | Kuwait | 432,278 (24.57) | 956,902 (32.04) | 1,600,107 (36.15) | 1.33 (1.30 - 1.36) | 1.35 (1.31 - 1.39) |
| North Africa and Middle East | Lebanon | 613,246 (18.72) | 1,051,356 (25.36) | 1,433,478 (27.69) | 1.38 (1.33 - 1.43) | 1.08 (0.97 - 1.19) |
| North Africa and Middle East | Libya | 719,933 (16.99) | 1,567,500 (25.77) | 2,033,932 (30.20) | 2.01 (1.99 - 2.04) | 1.78 (1.72 - 1.83) |
| North Africa and Middle East | Morocco | 4,416,953 (17.46) | 7,856,128 (23.48) | 9,908,795 (27.56) | 1.59 (1.58 - 1.60) | 1.80 (1.78 - 1.82) |
| North Africa and Middle East | Palestine | 288,457 (13.93) | 701,462 (16.88) | 962,178 (19.41) | 1.16 (1.13 - 1.18) | 1.59 (1.56 - 1.62) |
| North Africa and Middle East | Oman | 314,522 (16.19) | 682,853 (24.06) | 1,379,662 (30.10) | 2.18 (1.87 - 2.49) | 2.50 (1.97 - 3.03) |
| North Africa and Middle East | Qatar | 127,521 (28.65) | 659,259 (38.04) | 1,125,348 (39.29) | 1.09 (1.04 - 1.14) | 0.29 (0.25 - 0.33) |
| North Africa and Middle East | Saudi Arabia | 2,784,089 (17.35) | 7,438,721 (26.64) | 11,832,599 (33.11) | 2.26 (2.24 - 2.27) | 2.47 (2.44 - 2.49) |
| North Africa and Middle East | Syria | 1,997,332 (15.49) | 4,341,788 (20.75) | 3,785,734 (26.12) | 1.87 (1.73 - 2.01) | 2.68 (2.42 - 2.94) |
| North Africa and Middle East | Tunisia | 1,549,933 (18.37) | 2,840,422 (26.24) | 3,496,703 (30.22) | 1.74 (1.72 - 1.75) | 1.58 (1.55 - 1.62) |
| North Africa and Middle East | Turkey | 11,040,073 (18.47) | 19,371,876 (25.91) | 24,813,466 (30.50) | 1.75 (1.73 - 1.78) | 1.84 (1.79 - 1.89) |
| North Africa and Middle East | United Arab Emirates | 433,509 (23.16) | 2,782,937 (32.99) | 3,655,018 (39.55) | 1.86 (1.80 - 1.93) | 2.05 (1.95 - 2.15) |
| North Africa and Middle East | Yemen | 1,503,593 (10.95) | 3,272,368 (12.99) | 4,597,944 (14.60) | 1.01 (0.98 - 1.04) | 1.32 (1.27 - 1.38) |
| North Africa and Middle East | Afghanistan | 1,640,338 (14.37) | 3,552,529 (12.42) | 5,227,239 (13.66) | -0.16 (-0.21 - -0.11) | 1.11 (1.02 - 1.19) |
| North Africa and Middle East | Sudan | 1,910,892 (9.46) | 3,483,283 (10.35) | 5,242,711 (12.85) | 1.07 (1.03 - 1.11) | 2.52 (2.44 - 2.60) |
| Central Sub-Saharan Africa | Angola | 777,279 (7.53) | 1,687,664 (7.73) | 2,486,248 (8.25) | 0.32 (0.30 - 0.33) | 0.72 (0.70 - 0.74) |
| Central Sub-Saharan Africa | Central African Republic | 211,525 (7.71) | 366,070 (7.93) | 440,589 (8.31) | 0.27 (0.24 - 0.29) | 0.54 (0.48 - 0.60) |
| Central Sub-Saharan Africa | Congo | 197,318 (8.07) | 416,534 (9.89) | 580,176 (11.02) | 1.08 (1.07 - 1.10) | 1.21 (1.19 - 1.23) |
| Central Sub-Saharan Africa | Democratic Republic of the Congo | 2,935,756 (7.61) | 5,350,258 (7.81) | 7,447,467 (8.49) | 0.38 (0.36 - 0.39) | 0.94 (0.90 - 0.97) |
| Central Sub-Saharan Africa | Equatorial Guinea | 32,694 (7.60) | 88,946 (8.53) | 148,538 (10.46) | 1.13 (1.09 - 1.17) | 2.33 (2.30 - 2.37) |
| Central Sub-Saharan Africa | Gabon | 92,166 (9.30) | 180,768 (12.01) | 241,022 (13.77) | 1.37 (1.36 - 1.38) | 1.53 (1.51 - 1.54) |
| Eastern Sub-Saharan Africa | Burundi | 406,198 (7.29) | 677,748 (7.55) | 956,422 (8.01) | 0.31 (0.26 - 0.36) | 0.67 (0.56 - 0.79) |
| Eastern Sub-Saharan Africa | Comoros | 47,058 (10.10) | 84,237 (13.18) | 108,398 (15.17) | 1.41 (1.40 - 1.43) | 1.58 (1.57 - 1.59) |
| Eastern Sub-Saharan Africa | Djibouti | 35,493 (7.30) | 94,020 (10.20) | 141,140 (11.73) | 1.64 (1.60 - 1.67) | 1.56 (1.52 - 1.59) |
| Eastern Sub-Saharan Africa | Eritrea | 199,024 (6.63) | 416,078 (7.36) | 563,284 (8.39) | 0.82 (0.80 - 0.83) | 1.50 (1.49 - 1.52) |
| Eastern Sub-Saharan Africa | Ethiopia | 3,743,130 (7.28) | 6,500,364 (7.57) | 9,072,447 (8.43) | 0.51 (0.49 - 0.52) | 1.20 (1.17 - 1.22) |
| Eastern Sub-Saharan Africa | Kenya | 1,838,034 (7.92) | 3,990,068 (9.81) | 5,787,827 (11.52) | 1.30 (1.29 - 1.31) | 1.80 (1.79 - 1.81) |
| Eastern Sub-Saharan Africa | Madagascar | 887,198 (7.42) | 1,669,928 (7.91) | 2,353,108 (8.82) | 0.60 (0.58 - 0.62) | 1.23 (1.17 - 1.28) |
| Eastern Sub-Saharan Africa | Malawi | 831,726 (8.70) | 1,303,904 (9.13) | 1,845,634 (10.01) | 0.49 (0.46 - 0.53) | 1.02 (0.95 - 1.08) |
| Eastern Sub-Saharan Africa | Mozambique | 901,733 (6.90) | 1,541,230 (6.70) | 2,001,987 (6.78) | -0.09 (-0.14 - -0.03) | 0.11 (0.00 - 0.21) |
| Eastern Sub-Saharan Africa | Rwanda | 408,719 (5.70) | 670,039 (6.49) | 961,223 (7.58) | 0.99 (0.92 - 1.06) | 1.73 (1.65 - 1.80) |
| Eastern Sub-Saharan Africa | Somalia | 540,919 (7.57) | 1,114,547 (7.56) | 1,555,077 (7.64) | 0.03 (-0.00 - 0.06) | 0.13 (0.06 - 0.20) |
| Eastern Sub-Saharan Africa | Tanzania | 2,009,647 (7.76) | 3,913,560 (8.75) | 5,398,390 (9.51) | 0.71 (0.70 - 0.72) | 0.94 (0.92 - 0.96) |
| Eastern Sub-Saharan Africa | Uganda | 1,013,438 (5.85) | 1,884,807 (5.79) | 2,668,538 (6.49) | 0.36 (0.34 - 0.38) | 1.27 (1.24 - 1.29) |
| Eastern Sub-Saharan Africa | Zambia | 612,859 (7.72) | 1,148,291 (8.43) | 1,772,633 (9.72) | 0.79 (0.74 - 0.83) | 1.61 (1.51 - 1.71) |
| Eastern Sub-Saharan Africa | South Sudan | 486,633 (8.31) | 807,560 (8.64) | 878,984 (9.47) | 0.46 (0.42 - 0.50) | 1.03 (0.94 - 1.12) |
| Southern Sub-Saharan Africa | Botswana | 111,992 (8.60) | 238,270 (11.93) | 339,733 (14.53) | 1.82 (1.79 - 1.84) | 2.21 (2.16 - 2.27) |
| Southern Sub-Saharan Africa | Lesotho | 156,611 (8.67) | 214,728 (10.92) | 259,610 (12.41) | 1.25 (1.23 - 1.28) | 1.44 (1.41 - 1.47) |
| Southern Sub-Saharan Africa | Namibia | 115,712 (8.21) | 200,413 (9.48) | 252,106 (10.49) | 0.85 (0.83 - 0.87) | 1.13 (1.10 - 1.16) |
| Southern Sub-Saharan Africa | South Africa | 4,718,507 (12.81) | 8,406,405 (16.57) | 10,495,299 (18.88) | 1.35 (1.34 - 1.36) | 1.46 (1.45 - 1.47) |
| Southern Sub-Saharan Africa | Swaziland | 82,389 (10.21) | 145,991 (13.58) | 174,537 (15.28) | 1.42 (1.31 - 1.53) | 1.34 (1.11 - 1.58) |
| Southern Sub-Saharan Africa | Zimbabwe | 908,693 (8.79) | 1,281,706 (9.84) | 1,640,783 (10.93) | 0.74 (0.71 - 0.78) | 1.22 (1.19 - 1.25) |
| Western Sub-Saharan Africa | Benin | 341,534 (7.04) | 787,125 (8.38) | 1,112,806 (8.79) | 0.77 (0.74 - 0.80) | 0.54 (0.50 - 0.59) |
| Western Sub-Saharan Africa | Burkina Faso | 679,018 (7.10) | 1,327,433 (7.83) | 1,875,089 (8.26) | 0.52 (0.52 - 0.53) | 0.60 (0.58 - 0.62) |
| Western Sub-Saharan Africa | Cameroon | 1,056,215 (10.16) | 2,394,148 (10.89) | 3,487,559 (11.98) | 0.57 (0.56 - 0.58) | 1.07 (1.04 - 1.09) |
| Western Sub-Saharan Africa | Cape Verde | 34,071 (9.69) | 66,562 (12.99) | 88,832 (15.76) | 1.69 (1.67 - 1.71) | 2.16 (2.14 - 2.18) |
| Western Sub-Saharan Africa | Chad | 490,330 (8.14) | 915,221 (7.77) | 1,279,747 (7.80) | -0.15 (-0.16 - -0.14) | 0.03 (0.01 - 0.06) |
| Western Sub-Saharan Africa | Cote dIvoire | 1,009,587 (8.26) | 2,128,402 (9.83) | 2,821,055 (10.78) | 0.92 (0.91 - 0.93) | 1.02 (0.98 - 1.05) |
| Western Sub-Saharan Africa | The Gambia | 85,012 (8.57) | 179,774 (10.10) | 242,284 (10.79) | 0.80 (0.78 - 0.81) | 0.74 (0.71 - 0.76) |
| Western Sub-Saharan Africa | Ghana | 1,235,706 (8.23) | 2,710,388 (10.72) | 3,756,033 (11.91) | 1.29 (1.27 - 1.31) | 1.20 (1.17 - 1.23) |
| Western Sub-Saharan Africa | Guinea | 597,963 (9.67) | 953,116 (9.56) | 1,226,420 (9.70) | 0.01 (-0.02 - 0.05) | 0.15 (0.07 - 0.23) |
| Western Sub-Saharan Africa | Guinea-Bissau | 85,653 (8.50) | 142,790 (9.17) | 188,634 (9.92) | 0.53 (0.52 - 0.55) | 0.89 (0.86 - 0.91) |
| Western Sub-Saharan Africa | Liberia | 195,856 (9.97) | 417,630 (10.29) | 567,936 (11.86) | 0.62 (0.54 - 0.69) | 1.55 (1.49 - 1.61) |
| Western Sub-Saharan Africa | Mali | 863,235 (9.95) | 1,615,693 (10.16) | 2,267,885 (10.35) | 0.13 (0.12 - 0.14) | 0.19 (0.17 - 0.21) |
| Western Sub-Saharan Africa | Mauritania | 244,019 (11.81) | 454,484 (13.68) | 597,946 (14.90) | 0.80 (0.78 - 0.82) | 0.94 (0.90 - 0.99) |
| Western Sub-Saharan Africa | Niger | 636,534 (7.93) | 1,317,784 (7.99) | 1,826,070 (7.84) | -0.05 (-0.07 - -0.02) | -0.22 (-0.27 - -0.18) |
| Western Sub-Saharan Africa | Nigeria | 7,086,631 (7.86) | 13,147,202 (7.88) | 17,223,337 (8.02) | 0.06 (0.04 - 0.08) | 0.19 (0.13 - 0.24) |
| Western Sub-Saharan Africa | Sao Tome and Principe | 11,976 (9.85) | 20,013 (11.46) | 27,644 (13.46) | 1.09 (1.06 - 1.11) | 1.81 (1.77 - 1.86) |
| Western Sub-Saharan Africa | Senegal | 776,441 (10.19) | 1,520,394 (12.15) | 1,982,800 (13.10) | 0.87 (0.85 - 0.90) | 0.82 (0.77 - 0.87) |
| Western Sub-Saharan Africa | Sierra Leone | 362,953 (9.94) | 619,491 (9.73) | 876,181 (10.58) | 0.18 (0.12 - 0.25) | 0.90 (0.83 - 0.96) |
| Western Sub-Saharan Africa | Togo | 287,987 (7.86) | 626,485 (9.81) | 873,098 (11.02) | 1.17 (1.15 - 1.20) | 1.28 (1.23 - 1.34) |
| Abbreviations: CI, confidence interval; APC, Annual Percent Change | | | | | | |

| S Table 5. Among All ages (Children and Adults), NAFLD Prevalent Cases, Age-standardized Prevalence Rate, and Annual Percent Change in Age-standardized Rate During 1990-2019 and 2010-2019, By 204 Countries and Territories | | | | | | |
| --- | --- | --- | --- | --- | --- | --- |
|  |  | Prevalent cases (Age-Stand Prevalence %) | | | APC (95% CI) | |
| Region | Country | 1990 | 2010 | 2019 | 1990-2019 | 2010-2019 |
| Australasia | Australia | 1,365,098 (7.31) | 2,422,803 (9.18) | 2,959,992 (9.55) | 0.92 (0.89 - 0.95) | 0.40 (0.37 - 0.43) |
| Australasia | New Zealand | 259,821 (7.04) | 433,875 (8.50) | 500,107 (8.88) | 0.80 (0.78 - 0.82) | 0.46 (0.43 - 0.49) |
| High-income North America | Canada | 1,830,124 (5.88) | 2,875,744 (6.63) | 3,391,406 (6.88) | 0.53 (0.50 - 0.56) | 0.39 (0.32 - 0.46) |
| High-income North America | United States | 21,412,780 (7.41) | 33,920,854 (9.05) | 40,920,076 (9.69) | 0.93 (0.90 - 0.96) | 0.76 (0.70 - 0.81) |
| High-income North America | Greenland | 3,145 (5.86) | 3,992 (6.45) | 4,424 (6.68) | 0.46 (0.43 - 0.48) | 0.39 (0.36 - 0.43) |
| High-income Asia Pacific | Brunei | 14,384 (7.16) | 29,656 (8.05) | 37,775 (8.30) | 0.51 (0.49 - 0.53) | 0.34 (0.32 - 0.37) |
| High-income Asia Pacific | Japan | 10,370,150 (6.78) | 12,831,976 (6.86) | 14,371,841 (7.46) | 0.32 (0.28 - 0.37) | 0.92 (0.83 - 1.01) |
| High-income Asia Pacific | South Korea | 2,915,757 (6.90) | 5,283,046 (8.67) | 5,852,771 (8.02) | 0.38 (-0.08 - 0.84) | -1.25 (-2.00 - -0.49) |
| High-income Asia Pacific | Singapore | 234,912 (7.56) | 534,805 (8.82) | 678,203 (9.08) | 0.63 (0.60 - 0.66) | 0.31 (0.26 - 0.36) |
| Southern Latin America | Argentina | 1,974,336 (6.17) | 3,325,753 (7.74) | 3,977,457 (8.02) | 0.91 (0.89 - 0.93) | 0.39 (0.37 - 0.40) |
| Southern Latin America | Chile | 864,001 (7.17) | 1,685,406 (9.11) | 2,037,159 (9.43) | 0.94 (0.91 - 0.97) | 0.36 (0.33 - 0.39) |
| Southern Latin America | Uruguay | 302,087 (8.89) | 422,155 (10.79) | 468,990 (11.24) | 0.80 (0.76 - 0.85) | 0.42 (0.40 - 0.44) |
| Western Europe | Andorra | 4,731 (7.57) | 10,330 (9.37) | 11,061 (9.64) | 0.83 (0.79 - 0.86) | 0.32 (0.27 - 0.37) |
| Western Europe | Austria | 656,609 (6.86) | 958,783 (8.54) | 1,091,219 (8.95) | 0.91 (0.88 - 0.94) | 0.50 (0.45 - 0.56) |
| Western Europe | Belgium | 850,966 (6.91) | 1,202,838 (8.48) | 1,329,730 (8.82) | 0.84 (0.81 - 0.86) | 0.41 (0.38 - 0.44) |
| Western Europe | Cyprus | 58,602 (7.14) | 120,425 (8.90) | 153,099 (9.05) | 0.81 (0.80 - 0.83) | 0.18 (0.15 - 0.20) |
| Western Europe | Denmark | 387,017 (6.07) | 533,473 (7.55) | 590,321 (7.73) | 0.83 (0.80 - 0.86) | 0.26 (0.23 - 0.29) |
| Western Europe | Finland | 335,936 (5.49) | 497,506 (6.88) | 548,703 (7.12) | 0.91 (0.88 - 0.94) | 0.41 (0.36 - 0.45) |
| Western Europe | France | 4,366,743 (6.43) | 6,358,804 (7.82) | 7,060,640 (8.15) | 0.81 (0.79 - 0.83) | 0.44 (0.41 - 0.47) |
| Western Europe | Germany | 6,162,111 (6.02) | 8,694,195 (7.55) | 9,492,808 (7.89) | 0.93 (0.90 - 0.95) | 0.48 (0.44 - 0.53) |
| Western Europe | Greece | 968,012 (7.72) | 1,419,451 (9.42) | 1,394,277 (9.47) | 0.70 (0.67 - 0.73) | 0.02 (-0.00 - 0.04) |
| Western Europe | Iceland | 19,194 (7.17) | 33,124 (8.87) | 38,942 (9.19) | 0.85 (0.84 - 0.87) | 0.39 (0.37 - 0.40) |
| Western Europe | Ireland | 314,726 (8.50) | 560,899 (10.46) | 657,168 (10.94) | 0.87 (0.86 - 0.89) | 0.48 (0.45 - 0.51) |
| Western Europe | Israel | 554,627 (11.79) | 1,217,786 (15.09) | 1,547,067 (15.83) | 1.01 (0.99 - 1.04) | 0.51 (0.48 - 0.54) |
| Western Europe | Italy | 8,677,703 (12.14) | 12,317,463 (14.70) | 12,593,986 (14.55) | 0.56 (0.34 - 0.78) | -0.23 (-0.71 - 0.25) |
| Western Europe | Luxembourg | 33,675 (7.13) | 54,263 (8.53) | 71,313 (8.86) | 0.75 (0.73 - 0.77) | 0.41 (0.39 - 0.44) |
| Western Europe | Malta | 30,296 (7.37) | 53,434 (9.60) | 59,918 (9.77) | 0.98 (0.95 - 1.01) | 0.19 (0.14 - 0.24) |
| Western Europe | Netherlands | 1,321,225 (7.43) | 1,850,819 (8.69) | 2,071,519 (9.03) | 0.67 (0.64 - 0.69) | 0.37 (0.34 - 0.40) |
| Western Europe | Norway | 340,484 (6.61) | 484,764 (7.96) | 569,498 (8.25) | 0.76 (0.74 - 0.78) | 0.39 (0.36 - 0.41) |
| Western Europe | Portugal | 1,111,905 (9.39) | 1,715,232 (11.67) | 1,836,905 (11.97) | 0.83 (0.80 - 0.86) | 0.25 (0.22 - 0.29) |
| Western Europe | Spain | 4,180,355 (9.15) | 6,829,136 (10.76) | 7,348,821 (11.31) | 0.72 (0.70 - 0.75) | 0.55 (0.47 - 0.62) |
| Western Europe | Sweden | 777,117 (7.13) | 1,065,850 (8.64) | 1,213,633 (9.02) | 0.81 (0.79 - 0.83) | 0.47 (0.44 - 0.49) |
| Western Europe | Switzerland | 558,874 (6.54) | 811,459 (7.67) | 963,866 (8.10) | 0.74 (0.73 - 0.75) | 0.59 (0.57 - 0.61) |
| Western Europe | United Kingdom | 5,278,485 (7.54) | 7,434,353 (9.26) | 8,300,436 (9.57) | 0.82 (0.81 - 0.84) | 0.35 (0.33 - 0.37) |
| Western Europe | Monaco | 3,759 (8.56) | 5,302 (10.35) | 5,816 (10.71) | 0.77 (0.75 - 0.79) | 0.35 (0.33 - 0.37) |
| Western Europe | San Marino | 2,194 (7.89) | 3,800 (9.61) | 4,278 (9.76) | 0.73 (0.71 - 0.75) | 0.15 (0.12 - 0.19) |
| Central Europe | Albania | 420,174 (15.50) | 529,096 (16.23) | 584,546 (16.97) | 0.32 (0.30 - 0.33) | 0.49 (0.46 - 0.53) |
| Central Europe | Bosnia and Herzegovina | 763,914 (16.21) | 886,692 (18.36) | 850,731 (18.86) | 0.52 (0.51 - 0.54) | 0.29 (0.28 - 0.31) |
| Central Europe | Bulgaria | 1,151,676 (10.76) | 1,125,753 (10.80) | 1,113,143 (11.16) | 0.12 (0.11 - 0.14) | 0.36 (0.34 - 0.39) |
| Central Europe | Croatia | 665,800 (11.30) | 732,369 (12.36) | 753,562 (12.62) | 0.38 (0.36 - 0.40) | 0.21 (0.19 - 0.23) |
| Central Europe | Czech Republic | 1,213,858 (9.99) | 1,519,532 (10.73) | 1,631,958 (11.02) | 0.34 (0.33 - 0.34) | 0.29 (0.27 - 0.31) |
| Central Europe | Hungary | 1,346,429 (10.67) | 1,577,410 (11.66) | 1,633,702 (12.00) | 0.40 (0.38 - 0.42) | 0.30 (0.29 - 0.31) |
| Central Europe | Macedonia | 238,853 (11.74) | 327,428 (12.61) | 365,157 (13.00) | 0.35 (0.33 - 0.37) | 0.32 (0.29 - 0.36) |
| Central Europe | Montenegro | 77,148 (11.93) | 95,178 (12.33) | 101,666 (12.76) | 0.23 (0.22 - 0.24) | 0.38 (0.37 - 0.39) |
| Central Europe | Poland | 4,115,068 (9.82) | 5,216,734 (10.48) | 5,746,331 (10.98) | 0.38 (0.37 - 0.39) | 0.53 (0.52 - 0.54) |
| Central Europe | Romania | 2,764,983 (10.58) | 3,225,618 (11.77) | 3,295,031 (12.39) | 0.54 (0.53 - 0.56) | 0.57 (0.54 - 0.60) |
| Central Europe | Serbia | 1,225,638 (11.32) | 1,388,721 (11.87) | 1,439,997 (12.31) | 0.29 (0.27 - 0.30) | 0.39 (0.37 - 0.42) |
| Central Europe | Slovakia | 520,793 (9.15) | 673,507 (9.78) | 740,952 (10.16) | 0.36 (0.35 - 0.37) | 0.43 (0.42 - 0.44) |
| Central Europe | Slovenia | 254,207 (11.08) | 347,636 (12.46) | 371,635 (12.73) | 0.47 (0.46 - 0.48) | 0.22 (0.20 - 0.24) |
| Eastern Europe | Belarus | 1,180,628 (9.87) | 1,312,536 (10.39) | 1,380,370 (10.93) | 0.35 (0.33 - 0.36) | 0.55 (0.53 - 0.58) |
| Eastern Europe | Estonia | 176,816 (9.65) | 187,159 (10.53) | 196,267 (10.97) | 0.45 (0.42 - 0.48) | 0.45 (0.40 - 0.50) |
| Eastern Europe | Latvia | 313,096 (9.92) | 308,206 (10.75) | 296,954 (11.12) | 0.39 (0.38 - 0.40) | 0.36 (0.34 - 0.38) |
| Eastern Europe | Lithuania | 418,533 (10.04) | 447,731 (10.89) | 434,632 (11.12) | 0.35 (0.33 - 0.37) | 0.23 (0.19 - 0.26) |
| Eastern Europe | Moldova | 458,592 (10.08) | 522,526 (10.89) | 570,595 (11.68) | 0.51 (0.48 - 0.54) | 0.79 (0.73 - 0.85) |
| Eastern Europe | Russian Federation | 19,075,852 (11.10) | 22,504,527 (12.04) | 23,977,534 (12.55) | 0.42 (0.41 - 0.43) | 0.46 (0.44 - 0.48) |
| Eastern Europe | Ukraine | 7,118,763 (11.27) | 7,264,122 (11.78) | 7,255,010 (12.00) | 0.22 (0.19 - 0.24) | 0.21 (0.19 - 0.23) |
| Central Asia | Armenia | 393,984 (12.55) | 510,452 (14.20) | 547,959 (14.89) | 0.59 (0.58 - 0.61) | 0.51 (0.48 - 0.55) |
| Central Asia | Azerbaijan | 847,725 (13.85) | 1,384,074 (14.96) | 1,768,073 (15.79) | 0.45 (0.44 - 0.47) | 0.60 (0.57 - 0.62) |
| Central Asia | Georgia | 727,972 (12.21) | 629,380 (12.75) | 616,670 (13.30) | 0.30 (0.29 - 0.30) | 0.48 (0.46 - 0.50) |
| Central Asia | Kazakhstan | 1,709,857 (11.61) | 2,037,684 (12.32) | 2,448,967 (12.94) | 0.37 (0.36 - 0.38) | 0.53 (0.52 - 0.54) |
| Central Asia | Kyrgyzstan | 478,891 (13.65) | 685,456 (14.24) | 858,945 (14.73) | 0.27 (0.26 - 0.28) | 0.38 (0.37 - 0.39) |
| Central Asia | Mongolia | 171,552 (11.97) | 299,199 (12.01) | 402,490 (12.54) | 0.16 (0.14 - 0.17) | 0.48 (0.47 - 0.49) |
| Central Asia | Tajikistan | 399,778 (11.25) | 657,081 (11.51) | 928,176 (12.10) | 0.25 (0.21 - 0.28) | 0.53 (0.45 - 0.62) |
| Central Asia | Turkmenistan | 321,573 (12.48) | 560,080 (13.89) | 727,381 (15.11) | 0.67 (0.65 - 0.68) | 0.94 (0.92 - 0.96) |
| Central Asia | Uzbekistan | 1,809,210 (12.37) | 3,343,009 (13.90) | 4,516,647 (14.92) | 0.66 (0.63 - 0.69) | 0.82 (0.79 - 0.84) |
| Southeast Asia | Cambodia | 1,010,073 (15.66) | 1,897,659 (16.34) | 2,545,969 (17.02) | 0.29 (0.28 - 0.31) | 0.46 (0.45 - 0.48) |
| Southeast Asia | Indonesia | 25,705,107 (18.37) | 45,285,728 (20.10) | 56,324,162 (21.06) | 0.48 (0.47 - 0.49) | 0.54 (0.53 - 0.54) |
| Southeast Asia | Laos | 337,543 (12.30) | 613,144 (13.05) | 815,319 (13.51) | 0.33 (0.32 - 0.34) | 0.39 (0.38 - 0.41) |
| Southeast Asia | Malaysia | 2,532,217 (18.85) | 5,483,840 (21.08) | 7,067,747 (22.19) | 0.57 (0.56 - 0.58) | 0.57 (0.54 - 0.59) |
| Southeast Asia | Maldives | 20,255 (15.34) | 52,525 (17.47) | 95,083 (18.52) | 0.65 (0.63 - 0.67) | 0.64 (0.61 - 0.67) |
| Southeast Asia | Myanmar | 4,935,223 (16.16) | 7,881,166 (17.39) | 9,732,642 (18.13) | 0.40 (0.39 - 0.41) | 0.48 (0.46 - 0.49) |
| Southeast Asia | Philippines | 5,553,549 (12.89) | 10,771,201 (13.89) | 14,059,848 (14.40) | 0.39 (0.37 - 0.42) | 0.43 (0.41 - 0.45) |
| Southeast Asia | Sri Lanka | 2,212,000 (15.30) | 3,429,288 (16.10) | 4,189,860 (17.02) | 0.38 (0.33 - 0.42) | 0.66 (0.54 - 0.78) |
| Southeast Asia | Thailand | 7,349,703 (15.05) | 13,708,605 (17.29) | 16,983,772 (18.38) | 0.69 (0.69 - 0.70) | 0.68 (0.67 - 0.69) |
| Southeast Asia | Timor-Leste | 74,546 (14.72) | 117,061 (15.10) | 151,893 (15.44) | 0.17 (0.15 - 0.18) | 0.26 (0.22 - 0.30) |
| Southeast Asia | Vietnam | 6,669,624 (13.43) | 12,257,864 (14.11) | 15,662,604 (14.76) | 0.33 (0.31 - 0.34) | 0.51 (0.48 - 0.54) |
| Southeast Asia | Mauritius | 127,742 (13.19) | 212,980 (14.97) | 249,469 (15.60) | 0.58 (0.58 - 0.59) | 0.46 (0.45 - 0.47) |
| Southeast Asia | Seychelles | 11,069 (17.75) | 19,034 (19.23) | 23,736 (20.21) | 0.46 (0.41 - 0.50) | 0.60 (0.56 - 0.64) |
| East Asia | China | 133951892 (12.53) | 224013997 (13.68) | 293422204 (15.71) | 0.82 (0.62 - 1.02) | 1.90 (1.48 - 2.32) |
| East Asia | North Korea | 2,268,955 (11.83) | 3,579,799 (12.81) | 4,269,361 (13.49) | 0.45 (0.44 - 0.46) | 0.57 (0.55 - 0.60) |
| East Asia | Taiwan | 2,669,043 (13.71) | 4,646,860 (15.84) | 5,434,103 (16.55) | 0.68 (0.62 - 0.73) | 0.50 (0.38 - 0.62) |
| Oceania | Fiji | 101,916 (17.93) | 164,040 (20.14) | 185,650 (20.99) | 0.54 (0.53 - 0.56) | 0.45 (0.44 - 0.46) |
| Oceania | Kiribati | 9,004 (16.96) | 15,303 (18.79) | 18,047 (18.77) | 0.33 (0.26 - 0.40) | 0.00 (-0.17 - 0.17) |
| Oceania | Marshall Islands | 3,970 (15.34) | 6,937 (16.63) | 8,441 (17.26) | 0.41 (0.37 - 0.45) | 0.44 (0.36 - 0.52) |
| Oceania | Federated States of Micronesia | 11,240 (17.01) | 15,903 (19.06) | 17,102 (18.87) | 0.35 (0.33 - 0.38) | -0.11 (-0.16 - -0.06) |
| Oceania | Papua New Guinea | 413,697 (14.90) | 848,184 (15.71) | 1,186,420 (15.98) | 0.24 (0.24 - 0.25) | 0.20 (0.19 - 0.20) |
| Oceania | Samoa | 20,846 (18.33) | 29,067 (19.24) | 33,966 (19.42) | 0.20 (0.18 - 0.21) | 0.12 (0.09 - 0.15) |
| Oceania | Solomon Islands | 31,541 (15.48) | 64,808 (17.05) | 81,638 (17.17) | 0.36 (0.34 - 0.38) | 0.11 (0.06 - 0.17) |
| Oceania | Tonga | 12,184 (17.86) | 16,804 (19.86) | 17,380 (19.99) | 0.38 (0.34 - 0.43) | 0.10 (0.01 - 0.20) |
| Oceania | Vanuatu | 16,343 (16.73) | 32,514 (18.01) | 42,052 (18.50) | 0.34 (0.33 - 0.35) | 0.30 (0.28 - 0.32) |
| Oceania | American Samoa | 7,166 (20.30) | 10,981 (22.40) | 11,677 (22.40) | 0.34 (0.31 - 0.36) | 0.03 (0.00 - 0.06) |
| Oceania | Cook Islands | 2,809 (18.16) | 4,140 (21.10) | 4,534 (21.84) | 0.64 (0.61 - 0.66) | 0.41 (0.38 - 0.44) |
| Oceania | Guam | 19,927 (16.94) | 31,350 (18.93) | 35,294 (19.63) | 0.51 (0.50 - 0.53) | 0.41 (0.38 - 0.43) |
| Oceania | Nauru | 1,174 (17.41) | 1,296 (17.59) | 1,488 (19.07) | 0.32 (0.28 - 0.35) | 0.98 (0.95 - 1.02) |
| Oceania | Niue | 358 (17.42) | 353 (19.96) | 390 (20.58) | 0.58 (0.55 - 0.61) | 0.37 (0.34 - 0.40) |
| Oceania | Northern Mariana Islands | 7,384 (18.62) | 12,108 (20.83) | 10,766 (20.69) | 0.36 (0.30 - 0.41) | -0.12 (-0.19 - -0.06) |
| Oceania | Palau | 2,342 (17.78) | 4,116 (20.30) | 4,646 (20.88) | 0.55 (0.53 - 0.58) | 0.35 (0.33 - 0.37) |
| Oceania | Tokelau | 211 (16.12) | 203 (18.17) | 252 (19.07) | 0.58 (0.56 - 0.60) | 0.58 (0.55 - 0.60) |
| Oceania | Tuvalu | 1,239 (15.62) | 1,694 (17.45) | 2,004 (17.94) | 0.48 (0.45 - 0.50) | 0.33 (0.27 - 0.39) |
| South Asia | Bangladesh | 8,426,250 (12.49) | 16,350,935 (13.95) | 22,317,625 (14.79) | 0.58 (0.58 - 0.59) | 0.64 (0.63 - 0.65) |
| South Asia | Bhutan | 50,748 (13.04) | 91,675 (15.23) | 116,651 (16.28) | 0.78 (0.74 - 0.82) | 0.73 (0.70 - 0.76) |
| South Asia | India | 75,075,584 (11.98) | 130227795 (12.35) | 188363474 (14.19) | 0.59 (0.53 - 0.66) | 1.57 (1.44 - 1.70) |
| South Asia | Nepal | 1,509,370 (11.65) | 2,842,845 (13.23) | 3,947,139 (14.82) | 0.84 (0.82 - 0.85) | 1.31 (1.28 - 1.34) |
| South Asia | Pakistan | 10,381,084 (14.34) | 19,622,634 (16.18) | 27,097,727 (16.92) | 0.57 (0.55 - 0.59) | 0.48 (0.46 - 0.50) |
| Andean Latin America | Bolivia | 501,359 (11.58) | 1,049,016 (12.61) | 1,372,503 (13.04) | 0.41 (0.39 - 0.44) | 0.39 (0.35 - 0.43) |
| Andean Latin America | Ecuador | 1,331,828 (18.33) | 2,642,551 (19.87) | 3,491,265 (20.75) | 0.43 (0.40 - 0.46) | 0.52 (0.50 - 0.55) |
| Andean Latin America | Peru | 1,394,154 (8.84) | 2,687,249 (9.98) | 3,578,684 (10.45) | 0.58 (0.56 - 0.61) | 0.52 (0.50 - 0.55) |
| Caribbean | Antigua and Barbuda | 7,443 (13.67) | 13,777 (15.16) | 16,243 (15.63) | 0.46 (0.45 - 0.47) | 0.33 (0.32 - 0.34) |
| Caribbean | The Bahamas | 31,956 (15.10) | 58,705 (16.46) | 70,206 (16.65) | 0.34 (0.33 - 0.34) | 0.12 (0.10 - 0.13) |
| Caribbean | Barbados | 42,544 (16.13) | 59,660 (17.39) | 69,437 (17.85) | 0.35 (0.33 - 0.36) | 0.28 (0.27 - 0.29) |
| Caribbean | Belize | 18,953 (15.80) | 48,387 (18.55) | 66,908 (18.38) | 0.52 (0.50 - 0.54) | -0.10 (-0.13 - -0.07) |
| Caribbean | Cuba | 1,621,098 (14.87) | 2,349,773 (16.37) | 2,580,954 (17.04) | 0.47 (0.46 - 0.49) | 0.45 (0.44 - 0.47) |
| Caribbean | Dominica | 9,537 (14.68) | 12,120 (16.31) | 13,212 (16.60) | 0.42 (0.41 - 0.44) | 0.19 (0.16 - 0.22) |
| Caribbean | Dominican Republic | 627,747 (12.45) | 1,251,801 (14.31) | 1,555,728 (14.87) | 0.61 (0.56 - 0.66) | 0.41 (0.38 - 0.44) |
| Caribbean | Grenada | 9,430 (13.77) | 16,664 (15.58) | 18,332 (16.02) | 0.52 (0.51 - 0.53) | 0.31 (0.29 - 0.32) |
| Caribbean | Guyana | 79,244 (14.36) | 105,155 (15.97) | 121,651 (16.46) | 0.47 (0.46 - 0.48) | 0.32 (0.31 - 0.34) |
| Caribbean | Haiti | 559,861 (12.93) | 1,021,466 (13.34) | 1,360,875 (13.72) | 0.21 (0.20 - 0.22) | 0.31 (0.28 - 0.33) |
| Caribbean | Jamaica | 287,703 (15.26) | 466,896 (17.28) | 533,895 (17.61) | 0.49 (0.48 - 0.51) | 0.21 (0.20 - 0.22) |
| Caribbean | Saint Lucia | 14,185 (13.99) | 27,934 (15.95) | 34,128 (16.30) | 0.52 (0.51 - 0.54) | 0.23 (0.22 - 0.24) |
| Caribbean | Saint Vincent and the Grenadines | 11,206 (13.81) | 17,879 (15.81) | 20,903 (16.27) | 0.56 (0.55 - 0.58) | 0.32 (0.31 - 0.33) |
| Caribbean | Suriname | 45,872 (14.58) | 85,805 (16.21) | 103,625 (16.93) | 0.52 (0.50 - 0.53) | 0.48 (0.45 - 0.50) |
| Caribbean | Trinidad and Tobago | 159,590 (15.72) | 259,601 (17.19) | 303,550 (18.00) | 0.47 (0.45 - 0.49) | 0.52 (0.47 - 0.56) |
| Caribbean | Bermuda | 11,612 (17.21) | 16,141 (18.69) | 17,277 (18.92) | 0.32 (0.30 - 0.34) | 0.14 (0.11 - 0.18) |
| Caribbean | Puerto Rico | 637,743 (17.66) | 933,937 (20.20) | 976,434 (20.52) | 0.52 (0.51 - 0.53) | 0.18 (0.16 - 0.20) |
| Caribbean | Saint Kitts and Nevis | 5,278 (15.01) | 9,285 (16.39) | 11,836 (16.98) | 0.42 (0.41 - 0.44) | 0.38 (0.35 - 0.41) |
| Caribbean | Virgin Islands, U.S. | 15,646 (15.47) | 23,568 (18.01) | 24,164 (18.16) | 0.55 (0.52 - 0.59) | 0.10 (0.03 - 0.17) |
| Central Latin America | Colombia | 2,776,687 (11.26) | 5,416,908 (12.52) | 6,886,762 (13.26) | 0.56 (0.54 - 0.58) | 0.64 (0.61 - 0.68) |
| Central Latin America | Costa Rica | 265,870 (11.50) | 535,394 (12.34) | 661,290 (12.72) | 0.35 (0.34 - 0.35) | 0.34 (0.32 - 0.35) |
| Central Latin America | El Salvador | 508,761 (13.72) | 835,517 (15.79) | 982,775 (16.08) | 0.55 (0.54 - 0.56) | 0.19 (0.17 - 0.20) |
| Central Latin America | Guatemala | 833,380 (16.44) | 1,913,627 (18.03) | 2,727,663 (18.64) | 0.43 (0.42 - 0.44) | 0.36 (0.34 - 0.39) |
| Central Latin America | Honduras | 485,133 (16.93) | 1,100,113 (18.65) | 1,517,827 (18.97) | 0.39 (0.38 - 0.40) | 0.18 (0.17 - 0.19) |
| Central Latin America | Mexico | 9,491,947 (15.77) | 18,019,194 (17.10) | 22,589,109 (17.69) | 0.40 (0.39 - 0.41) | 0.37 (0.36 - 0.39) |
| Central Latin America | Nicaragua | 328,139 (14.48) | 717,986 (15.98) | 951,735 (16.54) | 0.46 (0.45 - 0.48) | 0.37 (0.36 - 0.38) |
| Central Latin America | Panama | 266,886 (14.14) | 528,502 (15.81) | 700,779 (16.68) | 0.57 (0.55 - 0.59) | 0.59 (0.55 - 0.64) |
| Central Latin America | Venezuela | 2,102,852 (15.16) | 4,334,253 (16.68) | 5,149,040 (17.17) | 0.43 (0.40 - 0.45) | 0.31 (0.26 - 0.36) |
| Tropical Latin America | Brazil | 15,327,546 (13.20) | 29,352,411 (14.65) | 37,141,599 (15.30) | 0.51 (0.50 - 0.52) | 0.49 (0.46 - 0.51) |
| Tropical Latin America | Paraguay | 326,509 (11.64) | 644,545 (12.64) | 845,074 (13.08) | 0.41 (0.40 - 0.42) | 0.38 (0.37 - 0.39) |
| North Africa and Middle East | Algeria | 3,970,015 (22.98) | 8,350,534 (25.01) | 11,118,713 (26.70) | 0.52 (0.51 - 0.53) | 0.74 (0.73 - 0.76) |
| North Africa and Middle East | Bahrain | 116,544 (26.64) | 385,793 (28.58) | 535,817 (30.28) | 0.44 (0.43 - 0.45) | 0.64 (0.63 - 0.66) |
| North Africa and Middle East | Egypt | 13,421,484 (31.45) | 24,441,821 (33.68) | 30,467,230 (34.52) | 0.33 (0.31 - 0.36) | 0.32 (0.25 - 0.38) |
| North Africa and Middle East | Iran | 9,354,195 (24.12) | 21,386,052 (28.53) | 25,559,769 (28.07) | 0.51 (0.44 - 0.57) | -0.33 (-0.45 - -0.20) |
| North Africa and Middle East | Iraq | 2,836,669 (24.95) | 6,441,938 (25.57) | 9,519,937 (26.91) | 0.27 (0.23 - 0.31) | 0.58 (0.48 - 0.67) |
| North Africa and Middle East | Jordan | 594,795 (24.94) | 1,615,982 (28.14) | 2,999,784 (29.38) | 0.57 (0.56 - 0.59) | 0.48 (0.46 - 0.50) |
| North Africa and Middle East | Kuwait | 432,278 (28.38) | 956,902 (30.56) | 1,600,107 (31.68) | 0.39 (0.37 - 0.41) | 0.40 (0.36 - 0.43) |
| North Africa and Middle East | Lebanon | 613,246 (23.04) | 1,051,356 (25.01) | 1,433,478 (26.55) | 0.49 (0.48 - 0.50) | 0.66 (0.64 - 0.68) |
| North Africa and Middle East | Libya | 719,933 (25.66) | 1,567,500 (27.89) | 2,033,932 (28.28) | 0.34 (0.31 - 0.36) | 0.17 (0.11 - 0.23) |
| North Africa and Middle East | Morocco | 4,416,953 (23.32) | 7,856,128 (25.59) | 9,908,795 (27.16) | 0.53 (0.52 - 0.54) | 0.68 (0.66 - 0.70) |
| North Africa and Middle East | Palestine | 288,457 (23.22) | 701,462 (24.82) | 962,178 (25.51) | 0.33 (0.30 - 0.37) | 0.34 (0.30 - 0.37) |
| North Africa and Middle East | Oman | 314,522 (23.15) | 682,853 (27.26) | 1,379,662 (29.22) | 0.81 (0.78 - 0.85) | 0.79 (0.72 - 0.86) |
| North Africa and Middle East | Qatar | 127,521 (29.92) | 659,259 (32.33) | 1,125,348 (33.32) | 0.38 (0.36 - 0.40) | 0.35 (0.32 - 0.37) |
| North Africa and Middle East | Saudi Arabia | 2,784,089 (24.98) | 7,438,721 (28.71) | 11,832,599 (30.53) | 0.69 (0.68 - 0.71) | 0.69 (0.65 - 0.72) |
| North Africa and Middle East | Syria | 1,997,332 (25.14) | 4,341,788 (26.78) | 3,785,734 (27.12) | 0.27 (0.25 - 0.29) | 0.14 (0.11 - 0.17) |
| North Africa and Middle East | Tunisia | 1,549,933 (23.73) | 2,840,422 (25.61) | 3,496,703 (27.01) | 0.45 (0.44 - 0.46) | 0.60 (0.58 - 0.61) |
| North Africa and Middle East | Turkey | 11,040,073 (23.08) | 19,371,876 (25.13) | 24,813,466 (26.65) | 0.50 (0.49 - 0.52) | 0.68 (0.64 - 0.72) |
| North Africa and Middle East | United Arab Emirates | 433,509 (26.22) | 2,782,937 (29.13) | 3,655,018 (30.54) | 0.53 (0.51 - 0.55) | 0.52 (0.49 - 0.55) |
| North Africa and Middle East | Yemen | 1,503,593 (19.80) | 3,272,368 (20.72) | 4,597,944 (21.12) | 0.23 (0.21 - 0.25) | 0.22 (0.19 - 0.26) |
| North Africa and Middle East | Afghanistan | 1,640,338 (20.78) | 3,552,529 (21.43) | 5,227,239 (22.29) | 0.26 (0.22 - 0.29) | 0.46 (0.38 - 0.53) |
| North Africa and Middle East | Sudan | 1,910,892 (14.89) | 3,483,283 (15.97) | 5,242,711 (18.09) | 0.66 (0.64 - 0.69) | 1.41 (1.36 - 1.46) |
| Central Sub-Saharan Africa | Angola | 777,279 (12.37) | 1,687,664 (13.01) | 2,486,248 (13.49) | 0.31 (0.29 - 0.32) | 0.42 (0.40 - 0.44) |
| Central Sub-Saharan Africa | Central African Republic | 211,525 (12.17) | 366,070 (12.61) | 440,589 (12.63) | 0.13 (0.11 - 0.16) | 0.04 (-0.01 - 0.09) |
| Central Sub-Saharan Africa | Congo | 197,318 (12.96) | 416,534 (13.89) | 580,176 (14.34) | 0.35 (0.34 - 0.36) | 0.37 (0.35 - 0.38) |
| Central Sub-Saharan Africa | Democratic Republic of the Congo | 2,935,756 (12.62) | 5,350,258 (12.82) | 7,447,467 (13.13) | 0.14 (0.13 - 0.15) | 0.26 (0.24 - 0.28) |
| Central Sub-Saharan Africa | Equatorial Guinea | 32,694 (12.32) | 88,946 (14.92) | 148,538 (15.98) | 0.91 (0.89 - 0.93) | 0.77 (0.73 - 0.81) |
| Central Sub-Saharan Africa | Gabon | 92,166 (13.33) | 180,768 (15.75) | 241,022 (16.46) | 0.73 (0.71 - 0.75) | 0.49 (0.48 - 0.50) |
| Eastern Sub-Saharan Africa | Burundi | 406,198 (12.09) | 677,748 (12.72) | 956,422 (12.96) | 0.24 (0.23 - 0.26) | 0.22 (0.21 - 0.23) |
| Eastern Sub-Saharan Africa | Comoros | 47,058 (16.23) | 84,237 (17.21) | 108,398 (17.71) | 0.30 (0.30 - 0.30) | 0.32 (0.32 - 0.33) |
| Eastern Sub-Saharan Africa | Djibouti | 35,493 (12.71) | 94,020 (13.82) | 141,140 (14.40) | 0.43 (0.42 - 0.44) | 0.45 (0.44 - 0.46) |
| Eastern Sub-Saharan Africa | Eritrea | 199,024 (11.50) | 416,078 (11.86) | 563,284 (12.26) | 0.23 (0.21 - 0.25) | 0.39 (0.37 - 0.41) |
| Eastern Sub-Saharan Africa | Ethiopia | 3,743,130 (12.56) | 6,500,364 (13.15) | 9,072,447 (13.52) | 0.25 (0.25 - 0.26) | 0.30 (0.28 - 0.32) |
| Eastern Sub-Saharan Africa | Kenya | 1,838,034 (14.47) | 3,990,068 (15.56) | 5,787,827 (16.26) | 0.40 (0.40 - 0.41) | 0.50 (0.48 - 0.51) |
| Eastern Sub-Saharan Africa | Madagascar | 887,198 (12.16) | 1,669,928 (12.64) | 2,353,108 (13.09) | 0.26 (0.25 - 0.26) | 0.39 (0.38 - 0.41) |
| Eastern Sub-Saharan Africa | Malawi | 831,726 (14.46) | 1,303,904 (15.34) | 1,845,634 (15.83) | 0.32 (0.28 - 0.37) | 0.38 (0.34 - 0.41) |
| Eastern Sub-Saharan Africa | Mozambique | 901,733 (10.92) | 1,541,230 (11.29) | 2,001,987 (11.46) | 0.17 (0.15 - 0.19) | 0.18 (0.14 - 0.22) |
| Eastern Sub-Saharan Africa | Rwanda | 408,719 (9.56) | 670,039 (9.99) | 961,223 (10.42) | 0.30 (0.28 - 0.31) | 0.47 (0.44 - 0.51) |
| Eastern Sub-Saharan Africa | Somalia | 540,919 (12.90) | 1,114,547 (13.22) | 1,555,077 (13.23) | 0.09 (0.08 - 0.10) | 0.01 (-0.01 - 0.02) |
| Eastern Sub-Saharan Africa | Tanzania | 2,009,647 (12.99) | 3,913,560 (13.99) | 5,398,390 (14.49) | 0.38 (0.37 - 0.39) | 0.40 (0.38 - 0.41) |
| Eastern Sub-Saharan Africa | Uganda | 1,013,438 (10.42) | 1,884,807 (10.75) | 2,668,538 (11.08) | 0.21 (0.20 - 0.22) | 0.33 (0.31 - 0.34) |
| Eastern Sub-Saharan Africa | Zambia | 612,859 (13.76) | 1,148,291 (14.54) | 1,772,633 (15.18) | 0.34 (0.32 - 0.36) | 0.49 (0.45 - 0.53) |
| Eastern Sub-Saharan Africa | South Sudan | 486,633 (13.61) | 807,560 (14.62) | 878,984 (15.01) | 0.34 (0.33 - 0.35) | 0.29 (0.28 - 0.30) |
| Southern Sub-Saharan Africa | Botswana | 111,992 (13.56) | 238,270 (15.05) | 339,733 (16.21) | 0.61 (0.58 - 0.64) | 0.83 (0.77 - 0.89) |
| Southern Sub-Saharan Africa | Lesotho | 156,611 (12.49) | 214,728 (13.82) | 259,610 (14.51) | 0.52 (0.51 - 0.53) | 0.54 (0.50 - 0.58) |
| Southern Sub-Saharan Africa | Namibia | 115,712 (12.25) | 200,413 (12.82) | 252,106 (13.05) | 0.22 (0.21 - 0.23) | 0.20 (0.17 - 0.22) |
| Southern Sub-Saharan Africa | South Africa | 4,718,507 (16.57) | 8,406,405 (18.15) | 10,495,299 (18.98) | 0.47 (0.46 - 0.48) | 0.50 (0.48 - 0.51) |
| Southern Sub-Saharan Africa | Swaziland | 82,389 (17.52) | 145,991 (19.48) | 174,537 (19.55) | 0.39 (0.35 - 0.43) | 0.05 (-0.04 - 0.15) |
| Southern Sub-Saharan Africa | Zimbabwe | 908,693 (14.88) | 1,281,706 (14.88) | 1,640,783 (15.36) | 0.11 (0.09 - 0.13) | 0.39 (0.37 - 0.41) |
| Western Sub-Saharan Africa | Benin | 341,534 (12.23) | 787,125 (13.98) | 1,112,806 (14.09) | 0.49 (0.46 - 0.52) | 0.08 (0.02 - 0.14) |
| Western Sub-Saharan Africa | Burkina Faso | 679,018 (11.85) | 1,327,433 (12.83) | 1,875,089 (13.07) | 0.34 (0.33 - 0.35) | 0.20 (0.19 - 0.22) |
| Western Sub-Saharan Africa | Cameroon | 1,056,215 (16.31) | 2,394,148 (17.18) | 3,487,559 (17.63) | 0.27 (0.26 - 0.27) | 0.29 (0.28 - 0.30) |
| Western Sub-Saharan Africa | Cape Verde | 34,071 (14.49) | 66,562 (16.02) | 88,832 (16.72) | 0.49 (0.48 - 0.50) | 0.47 (0.46 - 0.48) |
| Western Sub-Saharan Africa | Chad | 490,330 (13.61) | 915,221 (14.37) | 1,279,747 (14.59) | 0.24 (0.23 - 0.25) | 0.17 (0.16 - 0.19) |
| Western Sub-Saharan Africa | Cote dIvoire | 1,009,587 (14.09) | 2,128,402 (15.09) | 2,821,055 (15.53) | 0.33 (0.31 - 0.35) | 0.29 (0.26 - 0.32) |
| Western Sub-Saharan Africa | The Gambia | 85,012 (14.80) | 179,774 (16.00) | 242,284 (16.23) | 0.31 (0.29 - 0.33) | 0.15 (0.10 - 0.19) |
| Western Sub-Saharan Africa | Ghana | 1,235,706 (12.89) | 2,710,388 (14.80) | 3,756,033 (15.22) | 0.58 (0.55 - 0.60) | 0.32 (0.29 - 0.36) |
| Western Sub-Saharan Africa | Guinea | 597,963 (14.61) | 953,116 (15.12) | 1,226,420 (15.41) | 0.18 (0.17 - 0.20) | 0.20 (0.18 - 0.22) |
| Western Sub-Saharan Africa | Guinea-Bissau | 85,653 (14.34) | 142,790 (15.02) | 188,634 (15.20) | 0.20 (0.19 - 0.21) | 0.13 (0.12 - 0.14) |
| Western Sub-Saharan Africa | Liberia | 195,856 (14.82) | 417,630 (15.98) | 567,936 (16.54) | 0.39 (0.36 - 0.41) | 0.38 (0.37 - 0.40) |
| Western Sub-Saharan Africa | Mali | 863,235 (15.87) | 1,615,693 (17.32) | 2,267,885 (17.62) | 0.36 (0.35 - 0.37) | 0.19 (0.17 - 0.20) |
| Western Sub-Saharan Africa | Mauritania | 244,019 (18.33) | 454,484 (20.39) | 597,946 (20.90) | 0.45 (0.44 - 0.46) | 0.28 (0.26 - 0.29) |
| Western Sub-Saharan Africa | Niger | 636,534 (14.35) | 1,317,784 (14.90) | 1,826,070 (14.97) | 0.15 (0.14 - 0.15) | 0.05 (0.04 - 0.06) |
| Western Sub-Saharan Africa | Nigeria | 7,086,631 (11.89) | 13,147,202 (12.42) | 17,223,337 (12.49) | 0.16 (0.14 - 0.19) | 0.06 (-0.00 - 0.12) |
| Western Sub-Saharan Africa | Sao Tome and Principe | 11,976 (15.75) | 20,013 (16.85) | 27,644 (17.45) | 0.36 (0.34 - 0.37) | 0.39 (0.36 - 0.42) |
| Western Sub-Saharan Africa | Senegal | 776,441 (17.17) | 1,520,394 (18.23) | 1,982,800 (18.71) | 0.29 (0.27 - 0.31) | 0.26 (0.23 - 0.28) |
| Western Sub-Saharan Africa | Sierra Leone | 362,953 (14.68) | 619,491 (15.17) | 876,181 (15.60) | 0.20 (0.19 - 0.22) | 0.29 (0.27 - 0.31) |
| Western Sub-Saharan Africa | Togo | 287,987 (14.02) | 626,485 (14.81) | 873,098 (15.25) | 0.29 (0.28 - 0.30) | 0.32 (0.30 - 0.34) |
| Abbreviations: CI, confidence interval; APC, Annual Percent Change | | | | | | |

| Supplementary Table 6. Joinpoint Analysis of All-ages Crude NALFD-related Mortality Rate, Stratified by Region, 1990-2019 | | | |
| --- | --- | --- | --- |
| Region | Trend Segment | Years | APC (95% CI) |
| Global | 1 | 1990 - 1995 | 1.32 (1.14 - 1.50) |
| Global | 2 | 1995 - 2004 | -0.24 (-0.33 - -0.16) |
| Global | 3 | 2004 - 2011 | 0.61 (0.47 - 0.74) |
| Global | 4 | 2011 - 2016 | 1.93 (1.69 - 2.18) |
| Global | 5 | 2016 - 2019 | 1.36 (0.97 - 1.75) |
| Australasia | 1 | 1990 - 2000 | 0.90 (0.70 - 1.10) |
| Australasia | 2 | 2000 - 2005 | 2.03 (1.29 - 2.77) |
| Australasia | 3 | 2005 - 2009 | 4.40 (3.25 - 5.57) |
| Australasia | 4 | 2009 - 2014 | 2.92 (2.21 - 3.65) |
| Australasia | 5 | 2014 - 2019 | 0.78 (0.29 - 1.28) |
| High-income North America | 1 | 1990 - 1995 | 1.60 (1.15 - 2.06) |
| High-income North America | 2 | 1995 - 1999 | -0.41 (-1.32 - 0.52) |
| High-income North America | 3 | 1999 - 2010 | 1.91 (1.77 - 2.05) |
| High-income North America | 4 | 2010 - 2015 | 4.75 (4.15 - 5.36) |
| High-income North America | 5 | 2015 - 2019 | 0.71 (0.11 - 1.32) |
| High-income Asia Pacific | 1 | 1990 - 1994 | 3.41 (2.76 - 4.06) |
| High-income Asia Pacific | 2 | 1994 - 2000 | 4.72 (4.30 - 5.13) |
| High-income Asia Pacific | 3 | 2000 - 2009 | -0.67 (-0.86 - -0.47) |
| High-income Asia Pacific | 4 | 2009 - 2019 | 1.07 (0.90 - 1.23) |
| Southern Latin America | 1 | 1990 - 1993 | -1.76 (-2.99 - -0.51) |
| Southern Latin America | 2 | 1993 - 2006 | 0.52 (0.37 - 0.66) |
| Southern Latin America | 3 | 2006 - 2016 | 2.37 (2.15 - 2.59) |
| Southern Latin America | 4 | 2016 - 2019 | 0.63 (-0.52 - 1.81) |
| Western Europe | 1 | 1990 - 1999 | 0.50 (0.40 - 0.59) |
| Western Europe | 2 | 1999 - 2003 | -0.26 (-0.75 - 0.23) |
| Western Europe | 3 | 2003 - 2007 | -1.01 (-1.49 - -0.54) |
| Western Europe | 4 | 2007 - 2014 | -0.08 (-0.24 - 0.08) |
| Western Europe | 5 | 2014 - 2019 | 0.74 (0.53 - 0.95) |
| Central Europe | 1 | 1990 - 1995 | 1.97 (1.59 - 2.34) |
| Central Europe | 2 | 1995 - 2000 | -1.30 (-1.85 - -0.75) |
| Central Europe | 3 | 2000 - 2009 | 2.53 (2.33 - 2.72) |
| Central Europe | 4 | 2009 - 2013 | -1.33 (-2.17 - -0.47) |
| Central Europe | 5 | 2013 - 2019 | 0.96 (0.66 - 1.27) |
| Eastern Europe | 1 | 1990 - 1994 | 12.29 (9.21 - 15.45) |
| Eastern Europe | 2 | 1994 - 1998 | -1.12 (-5.43 - 3.39) |
| Eastern Europe | 3 | 1998 - 2005 | 9.63 (8.01 - 11.27) |
| Eastern Europe | 4 | 2005 - 2016 | 1.10 (0.43 - 1.78) |
| Eastern Europe | 5 | 2016 - 2019 | -1.97 (-6.05 - 2.29) |
| Central Asia | 1 | 1990 - 1995 | 7.48 (6.92 - 8.05) |
| Central Asia | 2 | 1995 - 1999 | 1.78 (0.62 - 2.95) |
| Central Asia | 3 | 1999 - 2005 | 4.01 (3.50 - 4.52) |
| Central Asia | 4 | 2005 - 2014 | 1.91 (1.68 - 2.15) |
| Central Asia | 5 | 2014 - 2019 | 0.40 (-0.11 - 0.91) |
| Southeast Asia | 1 | 1990 - 1995 | 1.08 (0.85 - 1.31) |
| Southeast Asia | 2 | 1995 - 2002 | 0.77 (0.61 - 0.93) |
| Southeast Asia | 3 | 2002 - 2006 | 1.26 (0.80 - 1.73) |
| Southeast Asia | 4 | 2006 - 2011 | 2.67 (2.37 - 2.96) |
| Southeast Asia | 5 | 2011 - 2019 | 1.73 (1.62 - 1.83) |
| East Asia | 1 | 1990 - 1995 | 0.88 (0.35 - 1.41) |
| East Asia | 2 | 1995 - 2000 | -0.43 (-1.08 - 0.23) |
| East Asia | 3 | 2000 - 2004 | -5.31 (-6.36 - -4.24) |
| East Asia | 4 | 2004 - 2011 | -1.17 (-1.58 - -0.76) |
| East Asia | 5 | 2011 - 2019 | 3.43 (3.15 - 3.72) |
| Oceania | 1 | 1990 - 1995 | -0.49 (-0.71 - -0.26) |
| Oceania | 2 | 1995 - 1999 | 0.82 (0.33 - 1.31) |
| Oceania | 3 | 1999 - 2004 | -0.06 (-0.37 - 0.25) |
| Oceania | 4 | 2004 - 2012 | 0.80 (0.67 - 0.94) |
| Oceania | 5 | 2012 - 2019 | 0.38 (0.24 - 0.53) |
| South Asia | 1 | 1990 - 1996 | 1.66 (1.01 - 2.31) |
| South Asia | 2 | 1996 - 2004 | -2.63 (-3.08 - -2.17) |
| South Asia | 3 | 2004 - 2012 | 0.99 (0.53 - 1.46) |
| South Asia | 4 | 2012 - 2019 | 2.81 (2.34 - 3.29) |
| Andean Latin America | 1 | 1990 - 1995 | 2.36 (1.67 - 3.05) |
| Andean Latin America | 2 | 1995 - 2001 | 0.27 (-0.39 - 0.93) |
| Andean Latin America | 3 | 2001 - 2006 | 1.60 (0.65 - 2.57) |
| Andean Latin America | 4 | 2006 - 2010 | 3.15 (1.66 - 4.67) |
| Andean Latin America | 5 | 2010 - 2019 | 0.77 (0.48 - 1.06) |
| Caribbean | 1 | 1990 - 1994 | 0.75 (-0.00 - 1.50) |
| Caribbean | 2 | 1994 - 2001 | -1.36 (-1.76 - -0.96) |
| Caribbean | 3 | 2001 - 2011 | 0.50 (0.26 - 0.74) |
| Caribbean | 4 | 2011 - 2015 | 4.15 (2.83 - 5.48) |
| Caribbean | 5 | 2015 - 2019 | 2.28 (1.40 - 3.17) |
| Central Latin America | 1 | 1990 - 1993 | 2.10 (0.67 - 3.54) |
| Central Latin America | 2 | 1993 - 2005 | 0.66 (0.47 - 0.85) |
| Central Latin America | 3 | 2005 - 2009 | 3.22 (1.81 - 4.66) |
| Central Latin America | 4 | 2009 - 2014 | 1.13 (0.28 - 1.99) |
| Central Latin America | 5 | 2014 - 2019 | 3.10 (2.45 - 3.75) |
| Tropical Latin America | 1 | 1990 - 2000 | 0.21 (-0.05 - 0.47) |
| Tropical Latin America | 2 | 2000 - 2006 | 1.32 (0.58 - 2.06) |
| Tropical Latin America | 3 | 2006 - 2010 | 4.46 (2.78 - 6.18) |
| Tropical Latin America | 4 | 2010 - 2014 | 1.37 (-0.24 - 3.00) |
| Tropical Latin America | 5 | 2014 - 2019 | 3.00 (2.26 - 3.75) |
| North Africa and Middle East | 1 | 1990 - 1995 | -0.79 (-1.08 - -0.50) |
| North Africa and Middle East | 2 | 1995 - 2000 | -0.07 (-0.46 - 0.33) |
| North Africa and Middle East | 3 | 2000 - 2004 | 3.25 (2.61 - 3.90) |
| North Africa and Middle East | 4 | 2004 - 2008 | 0.90 (0.28 - 1.53) |
| North Africa and Middle East | 5 | 2008 - 2019 | 1.81 (1.72 - 1.90) |
| Central Sub-Saharan Africa | 1 | 1990 - 1998 | -0.53 (-0.77 - -0.29) |
| Central Sub-Saharan Africa | 2 | 1998 - 2005 | -1.69 (-2.07 - -1.31) |
| Central Sub-Saharan Africa | 3 | 2005 - 2015 | -0.66 (-0.87 - -0.44) |
| Central Sub-Saharan Africa | 4 | 2015 - 2019 | 2.92 (2.10 - 3.75) |
| Eastern Sub-Saharan Africa | 1 | 1990 - 1993 | -0.55 (-1.07 - -0.04) |
| Eastern Sub-Saharan Africa | 2 | 1993 - 2011 | -0.98 (-1.01 - -0.94) |
| Eastern Sub-Saharan Africa | 3 | 2011 - 2019 | 0.73 (0.62 - 0.83) |
| Southern Sub-Saharan Africa | 1 | 1990 - 1994 | 2.91 (1.50 - 4.34) |
| Southern Sub-Saharan Africa | 2 | 1994 - 1998 | 5.02 (3.11 - 6.95) |
| Southern Sub-Saharan Africa | 3 | 1998 - 2006 | 0.59 (0.18 - 1.00) |
| Southern Sub-Saharan Africa | 4 | 2006 - 2013 | -1.43 (-1.95 - -0.90) |
| Southern Sub-Saharan Africa | 5 | 2013 - 2019 | -0.36 (-0.90 - 0.19) |
| Western Sub-Saharan Africa | 1 | 1990 - 1993 | -0.42 (-1.13 - 0.30) |
| Western Sub-Saharan Africa | 2 | 1993 - 2000 | -1.31 (-1.53 - -1.08) |
| Western Sub-Saharan Africa | 3 | 2000 - 2005 | -0.49 (-0.90 - -0.08) |
| Western Sub-Saharan Africa | 4 | 2005 - 2009 | -1.78 (-2.41 - -1.14) |
| Western Sub-Saharan Africa | 5 | 2009 - 2019 | -0.53 (-0.64 - -0.43) |
| Abbreviations: CI, confidence interval; APC, Annual Percent Change Trend Segment were detected by Joinpoint Regression Model | | | |

| S Table 7. NAFLD-related Liver Mortality, Age-standardized Mortality Rate, and Annual Percent Change in Age-standardized Rate During 1990-2019 and 2010-2019, By 204 Countries and Territories | | | | | | |
| --- | --- | --- | --- | --- | --- | --- |
|  |  | Prevalent cases (Age-Stand Prevalence %) | | | APC (95% CI) | |
| Region | Country | 1990 | 2010 | 2019 | 1990-2019 | 2010-2019 |
| Australasia | Australia | 221 (1.17) | 420 (1.31) | 551 (1.36) | 0.50 (0.26 - 0.75) | 0.26 (-0.23 - 0.76) |
| Australasia | New Zealand | 39 (1.03) | 66 (1.06) | 86 (1.11) | 0.23 (-0.02 - 0.49) | 0.28 (0.19 - 0.37) |
| High-income North America | Canada | 314 (0.99) | 599 (1.10) | 791 (1.15) | 0.52 (0.40 - 0.65) | 0.41 (0.20 - 0.62) |
| High-income North America | United States | 4,173 (1.35) | 6,513 (1.45) | 9,111 (1.71) | 0.81 (0.50 - 1.13) | 1.60 (0.90 - 2.31) |
| High-income North America | Greenland | 1 (1.49) | 1 (1.57) | 1 (1.53) | 0.07 (-0.21 - 0.36) | -0.33 (-0.57 - -0.10) |
| High-income Asia Pacific | Brunei | 1 (1.15) | 2 (1.24) | 3 (1.20) | 0.13 (-0.22 - 0.48) | -0.29 (-0.52 - -0.05) |
| High-income Asia Pacific | Japan | 1,844 (1.11) | 2,546 (0.80) | 2,752 (0.68) | -1.65 (-1.76 - -1.55) | -1.95 (-2.13 - -1.76) |
| High-income Asia Pacific | South Korea | 451 (1.58) | 929 (1.55) | 1,184 (1.35) | -0.53 (-0.78 - -0.28) | -1.55 (-2.05 - -1.05) |
| High-income Asia Pacific | Singapore | 15 (0.70) | 31 (0.68) | 46 (0.62) | -0.35 (-0.54 - -0.16) | -1.03 (-1.43 - -0.62) |
| Southern Latin America | Argentina | 534 (1.69) | 755 (1.65) | 958 (1.78) | 0.20 (-0.02 - 0.43) | 0.96 (0.46 - 1.45) |
| Southern Latin America | Chile | 348 (3.41) | 460 (2.54) | 607 (2.54) | -0.87 (-1.26 - -0.49) | 0.21 (-0.07 - 0.48) |
| Southern Latin America | Uruguay | 54 (1.42) | 59 (1.21) | 65 (1.18) | -0.69 (-1.03 - -0.35) | -0.35 (-1.17 - 0.47) |
| Western Europe | Andorra | 1 (1.68) | 2 (1.58) | 2 (1.61) | -0.13 (-0.22 - -0.04) | 0.21 (0.06 - 0.35) |
| Western Europe | Austria | 236 (2.14) | 244 (1.67) | 245 (1.43) | -1.41 (-1.71 - -1.10) | -1.61 (-1.73 - -1.49) |
| Western Europe | Belgium | 202 (1.36) | 257 (1.31) | 281 (1.24) | -0.28 (-0.47 - -0.08) | -0.63 (-0.93 - -0.32) |
| Western Europe | Cyprus | 13 (1.89) | 18 (1.38) | 22 (1.22) | -1.51 (-1.72 - -1.29) | -1.42 (-1.58 - -1.26) |
| Western Europe | Denmark | 81 (1.14) | 129 (1.41) | 124 (1.16) | 0.14 (-0.17 - 0.45) | -2.04 (-2.55 - -1.53) |
| Western Europe | Finland | 72 (1.06) | 154 (1.67) | 150 (1.43) | 1.00 (0.68 - 1.32) | -1.73 (-1.95 - -1.51) |
| Western Europe | France | 1,574 (2.01) | 1,659 (1.49) | 1,735 (1.31) | -1.45 (-1.70 - -1.20) | -1.36 (-1.78 - -0.93) |
| Western Europe | Germany | 2,436 (2.02) | 2,811 (1.75) | 3,086 (1.69) | -0.63 (-0.85 - -0.41) | -0.29 (-0.50 - -0.09) |
| Western Europe | Greece | 221 (1.49) | 211 (0.98) | 240 (0.96) | -1.53 (-1.77 - -1.29) | -0.24 (-0.59 - 0.12) |
| Western Europe | Iceland | 2 (0.72) | 3 (0.68) | 3 (0.64) | -0.42 (-0.62 - -0.21) | -0.76 (-0.99 - -0.53) |
| Western Europe | Ireland | 25 (0.65) | 55 (0.95) | 64 (0.88) | 1.00 (0.63 - 1.38) | -1.40 (-1.91 - -0.88) |
| Western Europe | Israel | 60 (1.26) | 114 (1.26) | 139 (1.16) | -0.32 (-0.53 - -0.10) | -1.08 (-1.66 - -0.50) |
| Western Europe | Italy | 3,047 (3.47) | 2,515 (1.93) | 2,552 (1.69) | -2.45 (-2.73 - -2.16) | -1.50 (-2.06 - -0.95) |
| Western Europe | Luxembourg | 11 (2.07) | 12 (1.56) | 13 (1.28) | -1.64 (-1.83 - -1.46) | -2.20 (-2.64 - -1.76) |
| Western Europe | Malta | 4 (1.05) | 6 (0.86) | 6 (0.74) | -1.18 (-1.34 - -1.02) | -1.71 (-2.17 - -1.26) |
| Western Europe | Netherlands | 193 (0.99) | 262 (0.92) | 311 (0.89) | -0.33 (-0.54 - -0.13) | -0.37 (-0.65 - -0.08) |
| Western Europe | Norway | 52 (0.80) | 58 (0.71) | 65 (0.67) | -0.56 (-0.79 - -0.33) | -0.76 (-1.04 - -0.47) |
| Western Europe | Portugal | 379 (2.88) | 310 (1.64) | 317 (1.42) | -2.43 (-2.63 - -2.22) | -1.52 (-1.94 - -1.11) |
| Western Europe | Spain | 1,122 (2.13) | 1,123 (1.33) | 1,167 (1.17) | -2.06 (-2.23 - -1.89) | -1.52 (-1.71 - -1.34) |
| Western Europe | Sweden | 101 (0.70) | 136 (0.75) | 155 (0.74) | 0.21 (0.01 - 0.40) | 0.08 (-0.21 - 0.37) |
| Western Europe | Switzerland | 102 (1.02) | 152 (1.06) | 166 (0.94) | -0.27 (-0.58 - 0.05) | -1.19 (-1.29 - -1.10) |
| Western Europe | United Kingdom | 664 (0.79) | 1,282 (1.25) | 1,502 (1.29) | 1.74 (1.49 - 2.00) | 0.31 (-0.03 - 0.66) |
| Western Europe | Monaco | 1 (1.34) | 2 (1.86) | 2 (1.74) | 0.92 (0.83 - 1.00) | -0.71 (-0.80 - -0.62) |
| Western Europe | San Marino | 1 (1.91) | 1 (1.86) | 1 (1.81) | -0.16 (-0.24 - -0.07) | -0.21 (-0.31 - -0.10) |
| Central Europe | Albania | 35 (1.81) | 35 (1.06) | 49 (1.15) | -1.60 (-2.01 - -1.19) | 0.82 (0.52 - 1.12) |
| Central Europe | Bosnia and Herzegovina | 54 (1.33) | 80 (1.46) | 87 (1.47) | 0.31 (-0.06 - 0.68) | 0.13 (-0.05 - 0.31) |
| Central Europe | Bulgaria | 158 (1.31) | 205 (1.58) | 204 (1.56) | 0.69 (0.38 - 1.00) | -0.04 (-0.49 - 0.41) |
| Central Europe | Croatia | 107 (1.68) | 116 (1.51) | 103 (1.22) | -1.17 (-1.54 - -0.79) | -2.35 (-3.07 - -1.62) |
| Central Europe | Czech Republic | 148 (1.10) | 157 (0.92) | 189 (0.98) | -0.39 (-0.78 - 0.01) | 0.64 (-0.36 - 1.65) |
| Central Europe | Hungary | 339 (2.44) | 263 (1.60) | 205 (1.15) | -2.55 (-3.04 - -2.05) | -3.60 (-4.44 - -2.75) |
| Central Europe | Macedonia | 21 (1.17) | 36 (1.36) | 44 (1.44) | 0.77 (0.55 - 0.98) | 0.80 (0.62 - 0.98) |
| Central Europe | Montenegro | 4 (0.71) | 7 (0.81) | 8 (0.83) | 0.72 (0.49 - 0.95) | 0.59 (0.18 - 1.00) |
| Central Europe | Poland | 635 (1.51) | 672 (1.19) | 683 (1.07) | -1.31 (-1.81 - -0.81) | -1.35 (-1.70 - -0.99) |
| Central Europe | Romania | 459 (1.65) | 794 (2.44) | 755 (2.16) | 0.98 (0.24 - 1.71) | -0.82 (-1.12 - -0.51) |
| Central Europe | Serbia | 110 (0.99) | 138 (0.95) | 151 (0.98) | 0.02 (-0.34 - 0.38) | 0.31 (-0.05 - 0.68) |
| Central Europe | Slovakia | 92 (1.57) | 125 (1.64) | 123 (1.41) | -0.42 (-0.79 - -0.05) | -1.83 (-2.49 - -1.16) |
| Central Europe | Slovenia | 44 (1.79) | 57 (1.63) | 57 (1.38) | -0.74 (-1.28 - -0.20) | -2.12 (-3.20 - -1.03) |
| Eastern Europe | Belarus | 81 (0.64) | 312 (2.30) | 239 (1.62) | 3.27 (2.51 - 4.03) | -4.13 (-5.60 - -2.64) |
| Eastern Europe | Estonia | 15 (0.75) | 30 (1.43) | 34 (1.52) | 2.36 (1.87 - 2.86) | -0.60 (-1.14 - -0.06) |
| Eastern Europe | Latvia | 26 (0.75) | 55 (1.67) | 47 (1.41) | 2.36 (1.52 - 3.21) | -1.81 (-2.53 - -1.09) |
| Eastern Europe | Lithuania | 34 (0.78) | 114 (2.52) | 90 (1.95) | 3.25 (2.28 - 4.23) | -2.97 (-3.49 - -2.45) |
| Eastern Europe | Moldova | 256 (5.68) | 362 (7.02) | 265 (4.68) | -0.55 (-1.56 - 0.48) | -3.49 (-4.09 - -2.88) |
| Eastern Europe | Russian Federation | 1,777 (1.01) | 5,413 (2.73) | 5,459 (2.55) | 3.43 (2.37 - 4.50) | -0.75 (-2.15 - 0.67) |
| Eastern Europe | Ukraine | 788 (1.14) | 1,783 (2.76) | 2,041 (3.17) | 3.96 (2.16 - 5.80) | 2.20 (0.44 - 3.99) |
| Central Asia | Armenia | 27 (1.07) | 119 (3.39) | 125 (3.14) | 3.78 (3.19 - 4.37) | -0.91 (-1.91 - 0.10) |
| Central Asia | Azerbaijan | 114 (2.36) | 235 (3.94) | 285 (3.92) | 1.83 (1.59 - 2.07) | -0.03 (-0.64 - 0.59) |
| Central Asia | Georgia | 113 (1.90) | 116 (2.01) | 124 (2.21) | 0.54 (0.05 - 1.03) | 1.73 (1.38 - 2.09) |
| Central Asia | Kazakhstan | 174 (1.41) | 590 (4.01) | 622 (3.67) | 3.30 (3.11 - 3.49) | -0.91 (-1.18 - -0.64) |
| Central Asia | Kyrgyzstan | 69 (2.23) | 148 (3.75) | 160 (3.26) | 1.34 (0.84 - 1.85) | -1.84 (-2.42 - -1.26) |
| Central Asia | Mongolia | 88 (8.68) | 217 (17.25) | 285 (15.31) | 1.92 (1.69 - 2.15) | -1.33 (-1.51 - -1.15) |
| Central Asia | Tajikistan | 57 (1.93) | 112 (2.99) | 172 (3.50) | 2.02 (1.56 - 2.48) | 1.73 (0.69 - 2.79) |
| Central Asia | Turkmenistan | 55 (2.81) | 117 (3.51) | 189 (4.39) | 1.89 (0.67 - 3.11) | 2.98 (1.86 - 4.11) |
| Central Asia | Uzbekistan | 271 (2.31) | 787 (5.01) | 1,106 (5.10) | 2.80 (2.33 - 3.28) | 0.14 (-0.65 - 0.94) |
| Southeast Asia | Cambodia | 359 (7.57) | 580 (6.75) | 890 (7.79) | 0.11 (0.04 - 0.17) | 1.71 (1.56 - 1.87) |
| Southeast Asia | Indonesia | 5,145 (5.43) | 9,174 (6.17) | 10,416 (5.74) | 0.17 (0.08 - 0.26) | -0.79 (-0.94 - -0.63) |
| Southeast Asia | Laos | 74 (3.54) | 93 (2.81) | 130 (3.03) | -0.51 (-0.60 - -0.41) | 0.93 (0.84 - 1.03) |
| Southeast Asia | Malaysia | 149 (1.67) | 418 (2.49) | 610 (2.49) | 1.44 (1.09 - 1.79) | -0.04 (-0.27 - 0.18) |
| Southeast Asia | Maldives | 2 (2.16) | 3 (1.75) | 5 (1.76) | -0.78 (-1.02 - -0.54) | -0.06 (-0.13 - 0.02) |
| Southeast Asia | Myanmar | 949 (3.56) | 1,350 (3.32) | 1,903 (3.79) | 0.26 (0.10 - 0.42) | 1.59 (1.23 - 1.95) |
| Southeast Asia | Philippines | 774 (2.63) | 1,263 (2.23) | 1,794 (2.38) | -0.31 (-0.49 - -0.14) | 0.71 (0.35 - 1.07) |
| Southeast Asia | Sri Lanka | 284 (2.64) | 359 (2.00) | 517 (2.19) | -0.63 (-1.10 - -0.16) | 0.92 (-0.18 - 2.02) |
| Southeast Asia | Thailand | 1,526 (4.28) | 3,565 (4.90) | 4,919 (4.92) | 0.44 (0.25 - 0.62) | 0.12 (-0.27 - 0.51) |
| Southeast Asia | Timor-Leste | 8 (2.80) | 15 (2.52) | 24 (3.05) | 0.32 (0.15 - 0.49) | 2.21 (1.75 - 2.68) |
| Southeast Asia | Vietnam | 1,427 (3.76) | 1,632 (2.43) | 2,432 (2.81) | -1.07 (-1.30 - -0.85) | 1.53 (1.02 - 2.04) |
| Southeast Asia | Mauritius | 19 (2.47) | 29 (2.14) | 30 (1.79) | -1.11 (-1.47 - -0.75) | -1.73 (-2.34 - -1.13) |
| Southeast Asia | Seychelles | 2 (3.22) | 3 (3.81) | 4 (3.75) | 0.60 (0.38 - 0.82) | -0.15 (-0.33 - 0.03) |
| East Asia | China | 20,652 (2.51) | 17,654 (1.24) | 23,789 (1.25) | -2.39 (-2.64 - -2.14) | 0.33 (0.07 - 0.58) |
| East Asia | North Korea | 358 (2.31) | 469 (1.85) | 615 (1.95) | -0.57 (-0.64 - -0.50) | 0.61 (0.43 - 0.79) |
| East Asia | Taiwan | 500 (3.47) | 882 (2.95) | 1,008 (2.60) | -0.99 (-1.38 - -0.59) | -1.75 (-2.32 - -1.18) |
| Oceania | Fiji | 6 (1.71) | 10 (1.81) | 13 (1.89) | 0.37 (0.19 - 0.56) | 0.59 (0.50 - 0.68) |
| Oceania | Kiribati | 2 (5.51) | 3 (4.90) | 3 (4.56) | -0.66 (-0.75 - -0.56) | -0.80 (-1.02 - -0.59) |
| Oceania | Marshall Islands | 1 (3.83) | 1 (3.32) | 1 (3.37) | -0.47 (-0.61 - -0.33) | 0.04 (-0.38 - 0.45) |
| Oceania | Federated States of Micronesia | 2 (3.99) | 2 (3.51) | 2 (3.59) | -0.37 (-0.48 - -0.26) | 0.25 (0.15 - 0.34) |
| Oceania | Papua New Guinea | 18 (0.81) | 36 (0.89) | 50 (0.91) | 0.37 (0.26 - 0.48) | 0.22 (0.06 - 0.37) |
| Oceania | Samoa | 2 (2.66) | 3 (2.26) | 3 (2.21) | -0.66 (-0.72 - -0.60) | -0.23 (-0.32 - -0.14) |
| Oceania | Solomon Islands | 5 (3.24) | 10 (3.28) | 12 (3.19) | -0.07 (-0.38 - 0.24) | -0.43 (-1.14 - 0.28) |
| Oceania | Tonga | 2 (4.53) | 4 (5.09) | 4 (5.04) | 0.34 (0.11 - 0.56) | -0.23 (-0.36 - -0.11) |
| Oceania | Vanuatu | 2 (3.24) | 4 (3.22) | 5 (3.08) | -0.19 (-0.44 - 0.07) | -0.51 (-0.99 - -0.02) |
| Oceania | American Samoa | 0 (2.16) | 1 (2.13) | 1 (2.14) | -0.07 (-0.33 - 0.18) | -0.20 (-0.29 - -0.12) |
| Oceania | Cook Islands | 0 (2.38) | 0 (2.18) | 1 (2.19) | -0.28 (-0.47 - -0.10) | 0.01 (-0.13 - 0.15) |
| Oceania | Guam | 2 (2.65) | 3 (1.92) | 4 (2.05) | -0.92 (-1.30 - -0.54) | 0.69 (0.52 - 0.85) |
| Oceania | Nauru | 0 (3.74) | 0 (3.73) | 0 (3.70) | -0.02 (-0.12 - 0.08) | -0.11 (-0.31 - 0.10) |
| Oceania | Niue | 0 (2.82) | 0 (2.90) | 0 (2.77) | -0.08 (-0.14 - -0.02) | -0.55 (-0.58 - -0.52) |
| Oceania | Northern Mariana Islands | 1 (4.21) | 1 (3.14) | 1 (2.97) | -1.19 (-1.26 - -1.13) | -0.64 (-0.73 - -0.55) |
| Oceania | Palau | 0 (2.52) | 0 (2.74) | 1 (2.61) | 0.15 (0.08 - 0.21) | -0.48 (-0.58 - -0.38) |
| Oceania | Tokelau | 0 (2.75) | 0 (2.29) | 0 (2.25) | -0.68 (-0.71 - -0.66) | -0.21 (-0.24 - -0.19) |
| Oceania | Tuvalu | 0 (3.31) | 0 (2.80) | 0 (2.85) | -0.50 (-0.57 - -0.44) | 0.24 (0.16 - 0.33) |
| South Asia | Bangladesh | 1,017 (2.03) | 1,142 (1.38) | 1,667 (1.34) | -1.31 (-1.61 - -1.01) | 0.11 (-0.34 - 0.57) |
| South Asia | Bhutan | 4 (1.62) | 9 (2.10) | 11 (2.06) | 0.85 (0.75 - 0.95) | -0.29 (-0.38 - -0.20) |
| South Asia | India | 9,201 (1.99) | 12,476 (1.53) | 17,772 (1.60) | -0.73 (-1.06 - -0.39) | 0.41 (-0.03 - 0.86) |
| South Asia | Nepal | 210 (2.19) | 335 (2.02) | 499 (2.38) | 0.35 (0.24 - 0.47) | 2.18 (1.95 - 2.41) |
| South Asia | Pakistan | 1,139 (2.00) | 1,648 (2.02) | 1,947 (1.80) | -0.36 (-0.46 - -0.26) | -1.33 (-1.41 - -1.24) |
| Andean Latin America | Bolivia | 262 (8.35) | 549 (8.58) | 726 (8.70) | 0.14 (0.00 - 0.27) | 0.17 (-0.13 - 0.47) |
| Andean Latin America | Ecuador | 280 (5.27) | 726 (6.78) | 952 (6.64) | 0.76 (0.53 - 0.99) | 0.06 (-0.15 - 0.27) |
| Andean Latin America | Peru | 709 (5.89) | 1,194 (5.10) | 1,455 (4.52) | -0.83 (-1.42 - -0.23) | -1.14 (-2.55 - 0.29) |
| Caribbean | Antigua and Barbuda | 2 (3.40) | 2 (2.57) | 3 (2.68) | -0.78 (-1.33 - -0.24) | 0.37 (0.02 - 0.73) |
| Caribbean | The Bahamas | 7 (4.32) | 8 (2.75) | 12 (2.96) | -1.26 (-1.45 - -1.07) | 0.69 (0.45 - 0.93) |
| Caribbean | Barbados | 7 (2.34) | 7 (1.89) | 10 (2.02) | -0.52 (-0.78 - -0.26) | 0.56 (0.28 - 0.84) |
| Caribbean | Belize | 4 (3.90) | 7 (3.96) | 12 (4.08) | 0.23 (-0.06 - 0.52) | 0.50 (0.19 - 0.80) |
| Caribbean | Cuba | 252 (2.44) | 304 (1.92) | 405 (2.15) | -0.23 (-0.77 - 0.31) | 2.11 (1.54 - 2.70) |
| Caribbean | Dominica | 3 (3.68) | 2 (2.39) | 2 (2.54) | -1.26 (-1.42 - -1.09) | 0.85 (0.44 - 1.27) |
| Caribbean | Dominican Republic | 231 (6.42) | 330 (4.52) | 548 (6.06) | -0.14 (-0.52 - 0.24) | 3.71 (2.95 - 4.49) |
| Caribbean | Grenada | 3 (4.14) | 3 (2.95) | 3 (2.93) | -1.17 (-1.50 - -0.84) | 0.20 (-0.03 - 0.43) |
| Caribbean | Guyana | 31 (7.77) | 32 (5.88) | 40 (6.22) | -0.50 (-1.30 - 0.30) | 0.83 (0.32 - 1.34) |
| Caribbean | Haiti | 211 (6.51) | 265 (4.93) | 328 (4.67) | -1.13 (-1.24 - -1.02) | -0.60 (-0.71 - -0.48) |
| Caribbean | Jamaica | 29 (1.64) | 32 (1.21) | 42 (1.38) | -0.36 (-1.13 - 0.42) | 1.13 (-0.13 - 2.40) |
| Caribbean | Saint Lucia | 4 (4.83) | 4 (2.35) | 6 (2.91) | -1.72 (-1.99 - -1.44) | 2.37 (1.68 - 3.06) |
| Caribbean | Saint Vincent and the Grenadines | 2 (3.17) | 2 (2.07) | 3 (2.60) | -0.69 (-0.98 - -0.40) | 2.57 (1.92 - 3.22) |
| Caribbean | Suriname | 12 (4.75) | 17 (3.67) | 23 (3.97) | -0.56 (-1.12 - 0.01) | 1.33 (0.85 - 1.81) |
| Caribbean | Trinidad and Tobago | 27 (3.25) | 28 (1.95) | 37 (2.06) | -1.42 (-1.93 - -0.90) | 0.81 (-0.04 - 1.66) |
| Caribbean | Bermuda | 2 (3.30) | 1 (1.31) | 2 (1.26) | -3.23 (-3.50 - -2.96) | -0.24 (-0.54 - 0.06) |
| Caribbean | Puerto Rico | 189 (5.29) | 187 (3.21) | 228 (3.27) | -1.70 (-2.15 - -1.25) | -0.39 (-1.04 - 0.27) |
| Caribbean | Saint Kitts and Nevis | 2 (6.49) | 1 (3.11) | 2 (3.55) | -1.95 (-2.58 - -1.31) | 1.23 (0.44 - 2.03) |
| Caribbean | Virgin Islands, U.S. | 4 (4.24) | 6 (3.89) | 7 (3.75) | -0.36 (-0.68 - -0.05) | -0.33 (-0.58 - -0.08) |
| Central Latin America | Colombia | 397 (2.36) | 775 (2.04) | 963 (1.80) | -0.97 (-1.59 - -0.36) | -1.02 (-1.81 - -0.23) |
| Central Latin America | Costa Rica | 64 (3.61) | 159 (4.22) | 221 (4.30) | 0.69 (0.26 - 1.13) | 1.11 (0.48 - 1.75) |
| Central Latin America | El Salvador | 172 (5.56) | 249 (5.03) | 328 (5.45) | 0.05 (-0.37 - 0.46) | 0.95 (0.38 - 1.53) |
| Central Latin America | Guatemala | 397 (9.99) | 766 (9.29) | 1,116 (9.66) | -0.06 (-0.57 - 0.46) | 0.76 (0.43 - 1.09) |
| Central Latin America | Honduras | 197 (9.30) | 509 (12.12) | 716 (12.43) | 1.06 (0.82 - 1.31) | 0.29 (-0.05 - 0.63) |
| Central Latin America | Mexico | 4,284 (9.63) | 7,718 (8.87) | 10,017 (8.55) | -0.36 (-0.70 - -0.02) | -0.29 (-0.90 - 0.32) |
| Central Latin America | Nicaragua | 84 (5.32) | 235 (7.64) | 333 (7.81) | 1.43 (0.78 - 2.08) | 0.34 (-0.51 - 1.21) |
| Central Latin America | Panama | 42 (2.85) | 85 (2.84) | 115 (2.76) | -0.02 (-0.31 - 0.26) | -0.18 (-0.59 - 0.23) |
| Central Latin America | Venezuela | 402 (4.11) | 626 (2.97) | 948 (3.26) | -0.92 (-1.65 - -0.18) | 0.19 (-0.97 - 1.37) |
| Tropical Latin America | Brazil | 2,046 (2.28) | 3,570 (2.01) | 4,751 (2.01) | -0.41 (-0.80 - -0.02) | 0.07 (-0.77 - 0.92) |
| Tropical Latin America | Paraguay | 26 (1.19) | 51 (1.22) | 77 (1.39) | 0.61 (0.25 - 0.97) | 1.54 (0.84 - 2.25) |
| North Africa and Middle East | Algeria | 208 (2.18) | 346 (1.77) | 550 (1.98) | -0.34 (-0.39 - -0.28) | 1.15 (1.02 - 1.28) |
| North Africa and Middle East | Bahrain | 5 (3.92) | 12 (3.84) | 21 (3.41) | -0.48 (-0.98 - 0.01) | -1.37 (-2.06 - -0.68) |
| North Africa and Middle East | Egypt | 3,175 (14.76) | 5,813 (16.03) | 7,572 (15.98) | 0.29 (-0.05 - 0.63) | -0.02 (-0.54 - 0.51) |
| North Africa and Middle East | Iran | 449 (2.21) | 879 (1.84) | 1,269 (1.92) | -0.46 (-0.63 - -0.29) | 0.61 (0.47 - 0.74) |
| North Africa and Middle East | Iraq | 147 (1.97) | 290 (1.98) | 422 (2.03) | 0.08 (-0.17 - 0.33) | 0.24 (-0.39 - 0.87) |
| North Africa and Middle East | Jordan | 25 (2.30) | 51 (1.92) | 95 (1.79) | -0.80 (-0.99 - -0.61) | -0.76 (-1.18 - -0.34) |
| North Africa and Middle East | Kuwait | 7 (1.15) | 21 (1.57) | 32 (1.41) | 0.83 (0.12 - 1.55) | -1.33 (-2.49 - -0.16) |
| North Africa and Middle East | Lebanon | 37 (1.92) | 70 (1.75) | 88 (1.72) | -0.42 (-0.52 - -0.31) | -0.34 (-0.51 - -0.17) |
| North Africa and Middle East | Libya | 46 (2.70) | 85 (2.61) | 122 (2.61) | -0.16 (-0.41 - 0.09) | -0.14 (-0.30 - 0.01) |
| North Africa and Middle East | Morocco | 252 (2.16) | 439 (2.02) | 620 (2.33) | 0.25 (-0.08 - 0.59) | 1.77 (1.03 - 2.52) |
| North Africa and Middle East | Palestine | 24 (3.10) | 41 (2.88) | 54 (2.84) | -0.30 (-0.48 - -0.13) | 0.08 (-0.07 - 0.22) |
| North Africa and Middle East | Oman | 15 (2.56) | 32 (3.33) | 40 (2.91) | 0.58 (0.01 - 1.16) | -1.07 (-1.45 - -0.68) |
| North Africa and Middle East | Qatar | 5 (6.05) | 17 (7.47) | 31 (7.22) | 0.71 (-0.32 - 1.75) | 0.56 (0.18 - 0.94) |
| North Africa and Middle East | Saudi Arabia | 252 (5.41) | 377 (4.62) | 511 (4.33) | -0.77 (-0.88 - -0.65) | -0.68 (-0.75 - -0.61) |
| North Africa and Middle East | Syria | 134 (2.90) | 236 (2.97) | 292 (2.97) | 0.05 (-0.19 - 0.30) | 0.30 (-0.04 - 0.65) |
| North Africa and Middle East | Tunisia | 81 (1.92) | 156 (1.83) | 200 (1.74) | -0.34 (-0.43 - -0.25) | -0.48 (-0.64 - -0.32) |
| North Africa and Middle East | Turkey | 515 (1.55) | 875 (1.39) | 1,265 (1.52) | -0.02 (-0.34 - 0.29) | 0.96 (0.71 - 1.22) |
| North Africa and Middle East | United Arab Emirates | 7 (2.16) | 30 (2.84) | 72 (2.22) | 0.13 (-0.36 - 0.62) | -2.89 (-4.24 - -1.53) |
| North Africa and Middle East | Yemen | 119 (2.91) | 213 (2.57) | 292 (2.52) | -0.48 (-0.56 - -0.39) | -0.20 (-0.31 - -0.10) |
| North Africa and Middle East | Afghanistan | 262 (4.04) | 329 (3.65) | 427 (3.68) | -0.24 (-0.34 - -0.13) | 0.24 (0.00 - 0.48) |
| North Africa and Middle East | Sudan | 248 (3.00) | 367 (2.78) | 486 (2.94) | -0.06 (-0.09 - -0.03) | 0.66 (0.57 - 0.75) |
| Central Sub-Saharan Africa | Angola | 154 (3.91) | 264 (3.50) | 380 (3.47) | -0.37 (-0.69 - -0.05) | 0.06 (-0.64 - 0.78) |
| Central Sub-Saharan Africa | Central African Republic | 42 (3.52) | 59 (3.34) | 76 (3.43) | -0.17 (-0.29 - -0.05) | 0.03 (-0.30 - 0.36) |
| Central Sub-Saharan Africa | Congo | 40 (3.83) | 63 (3.43) | 83 (3.28) | -0.40 (-0.73 - -0.06) | -0.28 (-0.85 - 0.30) |
| Central Sub-Saharan Africa | Democratic Republic of the Congo | 480 (3.14) | 709 (2.64) | 972 (2.66) | -0.50 (-0.77 - -0.23) | 0.33 (-0.35 - 1.02) |
| Central Sub-Saharan Africa | Equatorial Guinea | 5 (2.72) | 7 (2.11) | 10 (2.07) | -0.93 (-1.14 - -0.72) | -0.24 (-0.60 - 0.12) |
| Central Sub-Saharan Africa | Gabon | 18 (3.40) | 25 (3.17) | 32 (3.19) | -0.17 (-0.27 - -0.08) | 0.05 (-0.17 - 0.26) |
| Eastern Sub-Saharan Africa | Burundi | 110 (4.82) | 118 (3.78) | 163 (3.70) | -0.91 (-1.07 - -0.75) | -0.17 (-0.45 - 0.12) |
| Eastern Sub-Saharan Africa | Comoros | 8 (3.69) | 12 (3.40) | 17 (3.65) | -0.04 (-0.46 - 0.38) | 0.63 (-0.36 - 1.62) |
| Eastern Sub-Saharan Africa | Djibouti | 5 (3.69) | 12 (3.51) | 20 (3.63) | -0.02 (-0.17 - 0.13) | 0.59 (0.20 - 0.98) |
| Eastern Sub-Saharan Africa | Eritrea | 44 (4.63) | 99 (5.40) | 139 (5.53) | 0.64 (0.43 - 0.86) | 0.20 (-0.33 - 0.72) |
| Eastern Sub-Saharan Africa | Ethiopia | 1,087 (5.73) | 1,218 (4.17) | 1,589 (4.11) | -1.11 (-1.31 - -0.92) | -0.10 (-0.34 - 0.15) |
| Eastern Sub-Saharan Africa | Kenya | 527 (6.84) | 1,087 (7.55) | 1,485 (7.41) | 0.25 (0.15 - 0.36) | -0.28 (-0.41 - -0.15) |
| Eastern Sub-Saharan Africa | Madagascar | 204 (4.11) | 291 (3.92) | 395 (3.81) | -0.24 (-0.37 - -0.11) | -0.55 (-0.64 - -0.46) |
| Eastern Sub-Saharan Africa | Malawi | 213 (5.92) | 260 (5.07) | 326 (4.71) | -0.77 (-0.91 - -0.64) | -0.66 (-0.81 - -0.51) |
| Eastern Sub-Saharan Africa | Mozambique | 130 (2.51) | 217 (2.74) | 246 (2.51) | 0.01 (-0.14 - 0.15) | -0.92 (-1.08 - -0.77) |
| Eastern Sub-Saharan Africa | Rwanda | 191 (6.91) | 163 (4.23) | 242 (4.29) | -1.65 (-1.81 - -1.49) | 0.11 (-0.04 - 0.25) |
| Eastern Sub-Saharan Africa | Somalia | 121 (5.00) | 223 (4.94) | 290 (4.64) | -0.25 (-0.37 - -0.13) | -0.65 (-0.87 - -0.43) |
| Eastern Sub-Saharan Africa | Tanzania | 400 (3.86) | 644 (3.64) | 781 (3.30) | -0.59 (-0.75 - -0.43) | -1.23 (-1.44 - -1.02) |
| Eastern Sub-Saharan Africa | Uganda | 235 (3.86) | 354 (3.66) | 507 (3.79) | -0.08 (-0.17 - 0.02) | 0.50 (0.28 - 0.72) |
| Eastern Sub-Saharan Africa | Zambia | 171 (6.31) | 262 (5.88) | 386 (5.89) | -0.19 (-0.39 - -0.00) | 0.20 (-0.15 - 0.55) |
| Eastern Sub-Saharan Africa | South Sudan | 93 (4.09) | 117 (4.00) | 135 (3.79) | -0.26 (-0.36 - -0.17) | -0.67 (-0.88 - -0.47) |
| Southern Sub-Saharan Africa | Botswana | 17 (2.89) | 32 (3.14) | 39 (2.78) | -0.09 (-0.20 - 0.02) | -1.28 (-1.38 - -1.18) |
| Southern Sub-Saharan Africa | Lesotho | 26 (2.69) | 42 (3.79) | 52 (4.19) | 1.58 (1.37 - 1.78) | 1.09 (0.58 - 1.61) |
| Southern Sub-Saharan Africa | Namibia | 17 (2.47) | 25 (2.28) | 34 (2.45) | -0.05 (-0.30 - 0.19) | 0.67 (0.19 - 1.15) |
| Southern Sub-Saharan Africa | South Africa | 492 (2.28) | 915 (2.62) | 899 (2.08) | -0.29 (-0.70 - 0.12) | -2.40 (-2.59 - -2.21) |
| Southern Sub-Saharan Africa | Swaziland | 10 (3.30) | 27 (5.82) | 28 (4.81) | 1.27 (1.11 - 1.43) | -2.12 (-2.25 - -1.99) |
| Southern Sub-Saharan Africa | Zimbabwe | 138 (3.58) | 257 (4.77) | 268 (3.97) | 0.41 (0.17 - 0.65) | -2.00 (-2.55 - -1.45) |
| Western Sub-Saharan Africa | Benin | 85 (4.37) | 135 (3.91) | 182 (3.77) | -0.48 (-0.58 - -0.39) | -0.35 (-0.53 - -0.17) |
| Western Sub-Saharan Africa | Burkina Faso | 158 (4.15) | 171 (2.75) | 257 (3.05) | -0.86 (-1.48 - -0.24) | 1.76 (0.53 - 3.00) |
| Western Sub-Saharan Africa | Cameroon | 157 (3.69) | 254 (3.00) | 331 (2.76) | -0.99 (-1.13 - -0.86) | -0.86 (-1.20 - -0.51) |
| Western Sub-Saharan Africa | Cape Verde | 5 (2.01) | 10 (2.83) | 16 (3.81) | 2.30 (1.70 - 2.90) | 3.71 (2.69 - 4.73) |
| Western Sub-Saharan Africa | Chad | 110 (4.01) | 168 (4.00) | 215 (3.84) | -0.16 (-0.26 - -0.05) | -0.52 (-0.82 - -0.23) |
| Western Sub-Saharan Africa | Cote dIvoire | 157 (4.08) | 292 (3.79) | 332 (3.18) | -0.87 (-1.06 - -0.69) | -1.87 (-2.09 - -1.64) |
| Western Sub-Saharan Africa | The Gambia | 19 (5.58) | 47 (6.44) | 63 (6.63) | 0.63 (0.02 - 1.25) | 0.73 (0.01 - 1.45) |
| Western Sub-Saharan Africa | Ghana | 296 (4.94) | 434 (3.76) | 593 (3.79) | -0.91 (-1.07 - -0.76) | 0.29 (-0.05 - 0.62) |
| Western Sub-Saharan Africa | Guinea | 184 (5.82) | 290 (6.07) | 321 (5.96) | 0.13 (-0.01 - 0.28) | 0.01 (-0.32 - 0.34) |
| Western Sub-Saharan Africa | Guinea-Bissau | 21 (5.29) | 29 (5.08) | 39 (5.31) | -0.13 (-0.32 - 0.07) | 0.08 (-0.46 - 0.62) |
| Western Sub-Saharan Africa | Liberia | 51 (4.95) | 69 (4.52) | 83 (4.13) | -0.72 (-0.95 - -0.49) | -1.13 (-1.73 - -0.53) |
| Western Sub-Saharan Africa | Mali | 209 (5.28) | 260 (4.04) | 338 (3.93) | -0.99 (-1.09 - -0.90) | -0.26 (-0.50 - -0.03) |
| Western Sub-Saharan Africa | Mauritania | 51 (5.27) | 52 (3.41) | 63 (3.15) | -1.76 (-1.90 - -1.61) | -0.87 (-1.14 - -0.61) |
| Western Sub-Saharan Africa | Niger | 100 (3.69) | 164 (3.07) | 242 (3.15) | -0.60 (-0.75 - -0.46) | 0.12 (-0.30 - 0.55) |
| Western Sub-Saharan Africa | Nigeria | 1,889 (4.56) | 2,857 (4.56) | 3,341 (4.08) | -0.41 (-0.59 - -0.24) | -1.23 (-1.66 - -0.81) |
| Western Sub-Saharan Africa | Sao Tome and Principe | 3 (4.78) | 4 (4.34) | 4 (4.30) | -0.33 (-0.59 - -0.07) | 0.15 (-0.14 - 0.43) |
| Western Sub-Saharan Africa | Senegal | 104 (3.35) | 152 (2.69) | 218 (2.97) | -0.50 (-1.09 - 0.09) | 0.76 (-1.11 - 2.66) |
| Western Sub-Saharan Africa | Sierra Leone | 75 (4.09) | 80 (3.03) | 108 (3.01) | -0.96 (-1.34 - -0.57) | 0.28 (-0.49 - 1.06) |
| Western Sub-Saharan Africa | Togo | 51 (4.22) | 89 (3.56) | 117 (3.30) | -0.92 (-1.17 - -0.68) | -0.79 (-1.43 - -0.15) |
| Abbreviations: CI, confidence interval; APC, Annual Percent Change | | | | | | |

| S Table 8. Among All ages (Children and Adults), NAFLD-related Liver Mortality, Crude Mortality Rate, and Annual Percent Change in Crude Rate During 1990-2019 and 2010-2019, By 204 Countries and Territories | | | | | | |
| --- | --- | --- | --- | --- | --- | --- |
|  |  | Prevalent cases (Prevalence %) | | | Average APC (95% CI) | |
| Region | Country | 1990 | 2010 | 2019 | 1990-2019 | 2010-2019 |
| Australasia | Australia | 221 (1.31) | 420 (1.95) | 551 (2.24) | 1.89 (1.66 - 2.12) | 1.54 (1.16 - 1.92) |
| Australasia | New Zealand | 39 (1.13) | 66 (1.54) | 86 (1.92) | 1.87 (1.66 - 2.08) | 2.43 (2.00 - 2.87) |
| High-income North America | Canada | 314 (1.15) | 599 (1.78) | 791 (2.17) | 2.19 (2.09 - 2.28) | 2.10 (1.88 - 2.32) |
| High-income North America | United States | 4,173 (1.65) | 6,513 (2.11) | 9,111 (2.78) | 1.82 (1.57 - 2.07) | 2.89 (2.33 - 3.45) |
| High-income North America | Greenland | 1 (1.10) | 1 (1.66) | 1 (1.89) | 1.85 (1.63 - 2.07) | 1.40 (1.25 - 1.55) |
| High-income Asia Pacific | Brunei | 1 (0.41) | 2 (0.58) | 3 (0.74) | 2.02 (1.79 - 2.25) | 2.86 (2.64 - 3.07) |
| High-income Asia Pacific | Japan | 1,844 (1.46) | 2,546 (1.96) | 2,752 (2.15) | 1.33 (1.16 - 1.49) | 0.93 (0.45 - 1.41) |
| High-income Asia Pacific | South Korea | 451 (1.02) | 929 (1.88) | 1,184 (2.22) | 2.73 (2.45 - 3.01) | 1.71 (1.24 - 2.18) |
| High-income Asia Pacific | Singapore | 15 (0.48) | 31 (0.61) | 46 (0.80) | 1.79 (1.45 - 2.14) | 3.02 (2.69 - 3.34) |
| Southern Latin America | Argentina | 534 (1.61) | 755 (1.84) | 958 (2.12) | 0.96 (0.66 - 1.25) | 1.71 (1.23 - 2.19) |
| Southern Latin America | Chile | 348 (2.62) | 460 (2.76) | 607 (3.34) | 1.01 (0.72 - 1.31) | 2.32 (2.01 - 2.64) |
| Southern Latin America | Uruguay | 54 (1.72) | 59 (1.76) | 65 (1.89) | 0.25 (-0.05 - 0.56) | 0.58 (0.24 - 0.93) |
| Western Europe | Andorra | 1 (1.61) | 2 (2.31) | 2 (2.77) | 1.96 (1.76 - 2.15) | 2.08 (1.83 - 2.34) |
| Western Europe | Austria | 236 (3.03) | 244 (2.92) | 245 (2.74) | -0.43 (-0.71 - -0.14) | -0.71 (-1.00 - -0.41) |
| Western Europe | Belgium | 202 (2.03) | 257 (2.36) | 281 (2.46) | 0.73 (0.53 - 0.92) | 0.45 (0.15 - 0.74) |
| Western Europe | Cyprus | 13 (1.67) | 18 (1.56) | 22 (1.65) | -0.12 (-0.26 - 0.01) | 0.51 (0.27 - 0.76) |
| Western Europe | Denmark | 81 (1.57) | 129 (2.33) | 124 (2.13) | 1.14 (0.82 - 1.46) | -0.90 (-1.43 - -0.37) |
| Western Europe | Finland | 72 (1.44) | 154 (2.87) | 150 (2.72) | 2.22 (1.87 - 2.56) | -0.54 (-0.78 - -0.30) |
| Western Europe | France | 1,574 (2.72) | 1,659 (2.60) | 1,735 (2.62) | -0.13 (-0.39 - 0.13) | 0.15 (-0.28 - 0.59) |
| Western Europe | Germany | 2,436 (3.05) | 2,811 (3.44) | 3,086 (3.63) | 0.61 (0.48 - 0.75) | 0.67 (0.53 - 0.81) |
| Western Europe | Greece | 221 (2.13) | 211 (1.91) | 240 (2.32) | 0.25 (-0.02 - 0.52) | 2.00 (1.82 - 2.18) |
| Western Europe | Iceland | 2 (0.79) | 3 (0.93) | 3 (1.01) | 0.87 (0.62 - 1.13) | 0.89 (0.26 - 1.53) |
| Western Europe | Ireland | 25 (0.71) | 55 (1.19) | 64 (1.31) | 2.13 (1.80 - 2.46) | 0.66 (-0.13 - 1.46) |
| Western Europe | Israel | 60 (1.21) | 114 (1.45) | 139 (1.49) | 0.67 (0.48 - 0.87) | 0.11 (-0.33 - 0.56) |
| Western Europe | Italy | 3,047 (5.36) | 2,515 (4.17) | 2,552 (4.23) | -0.82 (-1.09 - -0.55) | 0.09 (-0.51 - 0.70) |
| Western Europe | Luxembourg | 11 (2.84) | 12 (2.38) | 13 (2.06) | -1.10 (-1.29 - -0.90) | -1.56 (-2.01 - -1.11) |
| Western Europe | Malta | 4 (1.19) | 6 (1.42) | 6 (1.47) | 0.74 (0.65 - 0.84) | 0.36 (0.24 - 0.49) |
| Western Europe | Netherlands | 193 (1.29) | 262 (1.58) | 311 (1.81) | 1.19 (0.97 - 1.41) | 1.55 (1.24 - 1.86) |
| Western Europe | Norway | 52 (1.22) | 58 (1.20) | 65 (1.22) | 0.05 (-0.18 - 0.29) | 0.04 (-0.22 - 0.30) |
| Western Europe | Portugal | 379 (3.74) | 310 (2.87) | 317 (2.98) | -0.80 (-0.99 - -0.61) | 0.51 (0.20 - 0.82) |
| Western Europe | Spain | 1,122 (2.89) | 1,123 (2.39) | 1,167 (2.54) | -0.42 (-0.65 - -0.18) | 0.69 (0.07 - 1.31) |
| Western Europe | Sweden | 101 (1.18) | 136 (1.44) | 155 (1.52) | 0.94 (0.67 - 1.21) | 0.73 (0.43 - 1.03) |
| Western Europe | Switzerland | 102 (1.48) | 152 (1.91) | 166 (1.89) | 0.86 (0.53 - 1.20) | -0.04 (-0.14 - 0.07) |
| Western Europe | United Kingdom | 664 (1.16) | 1,282 (2.02) | 1,502 (2.23) | 2.33 (2.11 - 2.54) | 1.08 (0.79 - 1.37) |
| Western Europe | Monaco | 1 (2.94) | 2 (4.42) | 2 (4.28) | 1.31 (1.23 - 1.40) | -0.37 (-0.47 - -0.28) |
| Western Europe | San Marino | 1 (2.60) | 1 (3.49) | 1 (3.78) | 1.33 (1.25 - 1.40) | 0.98 (0.88 - 1.09) |
| Central Europe | Albania | 35 (1.05) | 35 (1.22) | 49 (1.79) | 1.88 (1.52 - 2.24) | 4.49 (4.13 - 4.85) |
| Central Europe | Bosnia and Herzegovina | 54 (1.18) | 80 (2.13) | 87 (2.62) | 2.76 (2.48 - 3.04) | 2.31 (1.79 - 2.83) |
| Central Europe | Bulgaria | 158 (1.82) | 205 (2.75) | 204 (2.94) | 1.69 (1.46 - 1.93) | 0.78 (0.46 - 1.10) |
| Central Europe | Croatia | 107 (2.18) | 116 (2.64) | 103 (2.43) | 0.35 (-0.04 - 0.74) | -0.91 (-1.65 - -0.16) |
| Central Europe | Czech Republic | 148 (1.44) | 157 (1.50) | 189 (1.78) | 0.75 (0.35 - 1.15) | 1.85 (0.82 - 2.89) |
| Central Europe | Hungary | 339 (3.26) | 263 (2.63) | 205 (2.12) | -1.47 (-1.96 - -0.97) | -2.38 (-3.21 - -1.54) |
| Central Europe | Macedonia | 21 (1.05) | 36 (1.70) | 44 (2.06) | 2.38 (2.10 - 2.66) | 2.16 (2.03 - 2.29) |
| Central Europe | Montenegro | 4 (0.69) | 7 (1.12) | 8 (1.29) | 2.31 (2.07 - 2.55) | 1.85 (1.42 - 2.27) |
| Central Europe | Poland | 635 (1.66) | 672 (1.75) | 683 (1.78) | 0.12 (-0.34 - 0.58) | 0.07 (-0.26 - 0.40) |
| Central Europe | Romania | 459 (1.96) | 794 (3.84) | 755 (3.93) | 2.50 (1.79 - 3.21) | 0.81 (0.53 - 1.09) |
| Central Europe | Serbia | 110 (1.17) | 138 (1.53) | 151 (1.73) | 1.46 (1.13 - 1.79) | 1.24 (0.88 - 1.60) |
| Central Europe | Slovakia | 92 (1.74) | 125 (2.31) | 123 (2.27) | 0.88 (0.54 - 1.22) | -0.36 (-0.97 - 0.25) |
| Central Europe | Slovenia | 44 (2.21) | 57 (2.80) | 57 (2.76) | 0.66 (-0.09 - 1.41) | -0.40 (-0.92 - 0.12) |
| Eastern Europe | Belarus | 81 (0.78) | 312 (3.22) | 239 (2.52) | 4.16 (3.42 - 4.90) | -2.97 (-4.42 - -1.50) |
| Eastern Europe | Estonia | 15 (0.95) | 30 (2.24) | 34 (2.57) | 3.41 (2.92 - 3.90) | 0.41 (-0.12 - 0.93) |
| Eastern Europe | Latvia | 26 (0.98) | 55 (2.60) | 47 (2.44) | 3.35 (2.52 - 4.18) | -0.64 (-1.29 - 0.03) |
| Eastern Europe | Lithuania | 34 (0.94) | 114 (3.69) | 90 (3.21) | 4.40 (3.59 - 5.22) | -1.65 (-2.07 - -1.22) |
| Eastern Europe | Moldova | 256 (5.76) | 362 (9.35) | 265 (7.18) | 0.87 (-0.15 - 1.90) | -2.05 (-2.65 - -1.45) |
| Eastern Europe | Russian Federation | 1,777 (1.18) | 5,413 (3.72) | 5,459 (3.72) | 4.24 (3.18 - 5.32) | 0.02 (-1.36 - 1.43) |
| Eastern Europe | Ukraine | 788 (1.50) | 1,783 (3.85) | 2,041 (4.63) | 4.28 (2.56 - 6.03) | 2.58 (0.94 - 4.25) |
| Central Asia | Armenia | 27 (0.80) | 119 (3.82) | 125 (4.14) | 5.86 (5.26 - 6.45) | 0.93 (-0.05 - 1.92) |
| Central Asia | Azerbaijan | 114 (1.55) | 235 (2.54) | 285 (2.77) | 2.06 (1.87 - 2.24) | 0.97 (0.76 - 1.18) |
| Central Asia | Georgia | 113 (2.06) | 116 (2.92) | 124 (3.39) | 1.74 (1.11 - 2.38) | 2.08 (1.46 - 2.71) |
| Central Asia | Kazakhstan | 174 (1.06) | 590 (3.64) | 622 (3.38) | 3.99 (3.78 - 4.20) | -0.83 (-1.09 - -0.57) |
| Central Asia | Kyrgyzstan | 69 (1.54) | 148 (2.63) | 160 (2.45) | 1.66 (1.16 - 2.16) | -0.99 (-1.34 - -0.65) |
| Central Asia | Mongolia | 88 (4.06) | 217 (7.65) | 285 (8.41) | 2.58 (2.35 - 2.80) | 1.07 (0.56 - 1.58) |
| Central Asia | Tajikistan | 57 (1.06) | 112 (1.45) | 172 (1.81) | 1.87 (1.57 - 2.18) | 2.63 (1.91 - 3.34) |
| Central Asia | Turkmenistan | 55 (1.49) | 117 (2.54) | 189 (3.72) | 3.01 (1.66 - 4.38) | 4.20 (1.55 - 6.93) |
| Central Asia | Uzbekistan | 271 (1.30) | 787 (2.70) | 1,106 (3.28) | 3.38 (2.96 - 3.79) | 2.23 (1.93 - 2.54) |
| Southeast Asia | Cambodia | 359 (3.46) | 580 (3.99) | 890 (5.36) | 1.54 (1.45 - 1.64) | 3.45 (3.24 - 3.67) |
| Southeast Asia | Indonesia | 5,145 (2.78) | 9,174 (3.81) | 10,416 (4.01) | 1.29 (1.18 - 1.40) | 0.50 (0.25 - 0.75) |
| Southeast Asia | Laos | 74 (1.79) | 93 (1.46) | 130 (1.81) | 0.06 (-0.07 - 0.19) | 2.54 (2.41 - 2.67) |
| Southeast Asia | Malaysia | 149 (0.84) | 418 (1.49) | 610 (1.95) | 2.93 (2.70 - 3.16) | 3.05 (2.80 - 3.29) |
| Southeast Asia | Maldives | 2 (0.80) | 3 (0.89) | 5 (1.01) | 0.73 (0.60 - 0.87) | 1.37 (1.19 - 1.56) |
| Southeast Asia | Myanmar | 949 (2.31) | 1,350 (2.66) | 1,903 (3.48) | 1.48 (1.31 - 1.65) | 3.11 (2.73 - 3.50) |
| Southeast Asia | Philippines | 774 (1.22) | 1,263 (1.31) | 1,794 (1.60) | 0.95 (0.77 - 1.12) | 2.34 (1.99 - 2.70) |
| Southeast Asia | Sri Lanka | 284 (1.65) | 359 (1.75) | 517 (2.37) | 1.30 (0.77 - 1.82) | 3.36 (2.14 - 4.60) |
| Southeast Asia | Thailand | 1,526 (2.68) | 3,565 (5.27) | 4,919 (7.02) | 3.31 (2.98 - 3.65) | 3.36 (2.90 - 3.83) |
| Southeast Asia | Timor-Leste | 8 (1.08) | 15 (1.34) | 24 (1.76) | 1.78 (1.64 - 1.92) | 3.13 (2.83 - 3.43) |
| Southeast Asia | Vietnam | 1,427 (2.10) | 1,632 (1.82) | 2,432 (2.52) | 0.57 (0.33 - 0.81) | 3.53 (2.98 - 4.08) |
| Southeast Asia | Mauritius | 19 (1.73) | 29 (2.27) | 30 (2.36) | 1.09 (0.75 - 1.43) | 0.65 (0.07 - 1.23) |
| Southeast Asia | Seychelles | 2 (2.48) | 3 (3.48) | 4 (3.93) | 1.68 (1.49 - 1.88) | 1.39 (1.22 - 1.56) |
| East Asia | China | 20,652 (1.74) | 17,654 (1.29) | 23,789 (1.67) | -0.14 (-0.40 - 0.12) | 3.17 (2.91 - 3.44) |
| East Asia | North Korea | 358 (1.70) | 469 (1.85) | 615 (2.35) | 1.13 (1.05 - 1.21) | 2.72 (2.52 - 2.92) |
| East Asia | Taiwan | 500 (2.45) | 882 (3.81) | 1,008 (4.27) | 1.92 (1.52 - 2.32) | 0.97 (0.39 - 1.55) |
| Oceania | Fiji | 6 (0.82) | 10 (1.19) | 13 (1.43) | 1.94 (1.70 - 2.18) | 2.05 (1.89 - 2.21) |
| Oceania | Kiribati | 2 (2.79) | 3 (2.59) | 3 (2.59) | -0.28 (-0.44 - -0.12) | 0.01 (-0.39 - 0.42) |
| Oceania | Marshall Islands | 1 (1.42) | 1 (1.66) | 1 (2.04) | 1.24 (1.16 - 1.32) | 2.31 (2.17 - 2.45) |
| Oceania | Federated States of Micronesia | 2 (1.79) | 2 (2.00) | 2 (2.38) | 1.00 (0.89 - 1.12) | 1.99 (1.89 - 2.09) |
| Oceania | Papua New Guinea | 18 (0.43) | 36 (0.47) | 50 (0.51) | 0.56 (0.42 - 0.70) | 0.93 (0.73 - 1.14) |
| Oceania | Samoa | 2 (1.40) | 3 (1.52) | 3 (1.51) | 0.25 (0.16 - 0.33) | -0.11 (-0.30 - 0.08) |
| Oceania | Solomon Islands | 5 (1.57) | 10 (1.78) | 12 (1.86) | 0.58 (0.27 - 0.89) | 0.33 (-0.41 - 1.07) |
| Oceania | Tonga | 2 (2.52) | 4 (3.48) | 4 (3.89) | 1.49 (1.28 - 1.70) | 1.13 (0.94 - 1.33) |
| Oceania | Vanuatu | 2 (1.43) | 4 (1.69) | 5 (1.81) | 0.75 (0.49 - 1.01) | 0.71 (0.53 - 0.88) |
| Oceania | American Samoa | 0 (1.01) | 1 (1.44) | 1 (1.75) | 1.96 (1.80 - 2.12) | 2.16 (2.09 - 2.24) |
| Oceania | Cook Islands | 0 (1.54) | 0 (2.28) | 1 (2.96) | 2.31 (2.11 - 2.52) | 2.97 (2.68 - 3.26) |
| Oceania | Guam | 2 (1.29) | 3 (1.76) | 4 (2.25) | 1.98 (1.58 - 2.39) | 2.97 (2.81 - 3.12) |
| Oceania | Nauru | 0 (1.51) | 0 (1.51) | 0 (1.56) | 0.13 (0.01 - 0.26) | 0.44 (0.24 - 0.65) |
| Oceania | Niue | 0 (2.67) | 0 (3.58) | 0 (3.49) | 0.90 (0.82 - 0.98) | -0.35 (-0.40 - -0.29) |
| Oceania | Northern Mariana Islands | 1 (1.82) | 1 (2.21) | 1 (3.25) | 2.03 (1.79 - 2.28) | 4.36 (4.26 - 4.46) |
| Oceania | Palau | 0 (1.59) | 0 (2.40) | 1 (2.91) | 2.11 (2.03 - 2.20) | 2.15 (2.00 - 2.30) |
| Oceania | Tokelau | 0 (2.11) | 0 (2.07) | 0 (2.03) | -0.16 (-0.24 - -0.08) | -0.34 (-0.44 - -0.25) |
| Oceania | Tuvalu | 0 (2.33) | 0 (2.23) | 0 (2.36) | 0.04 (-0.01 - 0.10) | 0.62 (0.54 - 0.70) |
| South Asia | Bangladesh | 1,017 (0.93) | 1,142 (0.79) | 1,667 (1.05) | 0.55 (0.20 - 0.90) | 3.52 (3.08 - 3.96) |
| South Asia | Bhutan | 4 (0.71) | 9 (1.28) | 11 (1.51) | 2.59 (2.43 - 2.75) | 1.74 (1.56 - 1.91) |
| South Asia | India | 9,201 (1.08) | 12,476 (1.01) | 17,772 (1.28) | 0.66 (0.38 - 0.94) | 2.63 (2.17 - 3.09) |
| South Asia | Nepal | 210 (1.08) | 335 (1.21) | 499 (1.64) | 1.54 (1.38 - 1.69) | 3.83 (3.51 - 4.16) |
| South Asia | Pakistan | 1,139 (1.01) | 1,648 (0.90) | 1,947 (0.87) | -0.51 (-0.59 - -0.44) | -0.39 (-0.44 - -0.34) |
| Andean Latin America | Bolivia | 262 (4.08) | 549 (5.40) | 726 (6.04) | 1.38 (1.28 - 1.48) | 1.26 (1.01 - 1.51) |
| Andean Latin America | Ecuador | 280 (2.79) | 726 (4.86) | 952 (5.41) | 2.21 (1.87 - 2.55) | 1.26 (0.77 - 1.75) |
| Andean Latin America | Peru | 709 (3.26) | 1,194 (4.13) | 1,455 (4.28) | 1.01 (0.41 - 1.62) | 0.62 (-0.83 - 2.08) |
| Caribbean | Antigua and Barbuda | 2 (3.02) | 2 (2.31) | 3 (2.94) | -0.08 (-0.73 - 0.57) | 2.65 (2.35 - 2.95) |
| Caribbean | The Bahamas | 7 (2.66) | 8 (2.32) | 12 (3.06) | 0.51 (0.15 - 0.86) | 3.13 (2.18 - 4.08) |
| Caribbean | Barbados | 7 (2.62) | 7 (2.59) | 10 (3.26) | 0.72 (0.41 - 1.04) | 2.33 (1.91 - 2.75) |
| Caribbean | Belize | 4 (1.95) | 7 (2.28) | 12 (2.83) | 1.37 (1.06 - 1.69) | 2.67 (2.36 - 2.99) |
| Caribbean | Cuba | 252 (2.32) | 304 (2.65) | 405 (3.56) | 1.70 (1.14 - 2.26) | 4.26 (3.67 - 4.87) |
| Caribbean | Dominica | 3 (3.54) | 2 (2.82) | 2 (3.30) | -0.23 (-0.45 - -0.01) | 1.96 (1.43 - 2.48) |
| Caribbean | Dominican Republic | 231 (3.21) | 330 (3.36) | 548 (5.04) | 1.63 (1.28 - 1.97) | 4.91 (4.04 - 5.78) |
| Caribbean | Grenada | 3 (3.47) | 3 (2.50) | 3 (3.13) | -0.29 (-0.51 - -0.07) | 2.74 (2.57 - 2.91) |
| Caribbean | Guyana | 31 (4.02) | 32 (4.31) | 40 (5.22) | 1.13 (0.86 - 1.40) | 2.41 (2.14 - 2.69) |
| Caribbean | Haiti | 211 (3.31) | 265 (2.57) | 328 (2.64) | -0.76 (-0.89 - -0.64) | 0.27 (0.14 - 0.41) |
| Caribbean | Jamaica | 29 (1.24) | 32 (1.15) | 42 (1.50) | 0.75 (-0.18 - 1.67) | 2.54 (1.88 - 3.21) |
| Caribbean | Saint Lucia | 4 (2.96) | 4 (2.24) | 6 (3.53) | 0.61 (0.37 - 0.85) | 5.14 (4.54 - 5.75) |
| Caribbean | Saint Vincent and the Grenadines | 2 (2.04) | 2 (2.01) | 3 (3.06) | 1.34 (0.90 - 1.79) | 4.80 (3.76 - 5.84) |
| Caribbean | Suriname | 12 (3.20) | 17 (3.07) | 23 (4.08) | 0.91 (0.26 - 1.56) | 3.79 (3.23 - 4.35) |
| Caribbean | Trinidad and Tobago | 27 (2.26) | 28 (2.08) | 37 (2.70) | 0.75 (0.20 - 1.30) | 3.09 (2.18 - 4.01) |
| Caribbean | Bermuda | 2 (3.37) | 1 (2.02) | 2 (2.56) | -0.91 (-1.18 - -0.64) | 2.88 (2.58 - 3.19) |
| Caribbean | Puerto Rico | 189 (5.24) | 187 (4.92) | 228 (6.48) | 0.66 (0.17 - 1.15) | 2.52 (1.81 - 3.23) |
| Caribbean | Saint Kitts and Nevis | 2 (5.64) | 1 (2.71) | 2 (3.85) | -1.24 (-1.81 - -0.67) | 3.52 (2.61 - 4.44) |
| Caribbean | Virgin Islands, U.S. | 4 (3.31) | 6 (5.37) | 7 (6.36) | 2.34 (1.96 - 2.72) | 2.02 (1.72 - 2.33) |
| Central Latin America | Colombia | 397 (1.22) | 775 (1.73) | 963 (2.02) | 1.71 (1.14 - 2.28) | 2.02 (1.34 - 2.71) |
| Central Latin America | Costa Rica | 64 (2.11) | 159 (3.62) | 221 (4.68) | 2.89 (2.47 - 3.31) | 4.02 (3.40 - 4.64) |
| Central Latin America | El Salvador | 172 (3.26) | 249 (4.14) | 328 (5.24) | 1.70 (1.24 - 2.15) | 2.35 (2.11 - 2.59) |
| Central Latin America | Guatemala | 397 (4.98) | 766 (5.27) | 1,116 (6.28) | 0.84 (0.33 - 1.35) | 2.28 (1.97 - 2.59) |
| Central Latin America | Honduras | 197 (4.18) | 509 (6.37) | 716 (7.29) | 2.00 (1.76 - 2.24) | 1.45 (1.16 - 1.75) |
| Central Latin America | Mexico | 4,284 (5.01) | 7,718 (6.75) | 10,017 (8.02) | 1.67 (1.33 - 2.02) | 2.05 (1.42 - 2.69) |
| Central Latin America | Nicaragua | 84 (2.15) | 235 (4.09) | 333 (5.12) | 3.09 (2.49 - 3.71) | 2.54 (1.64 - 3.46) |
| Central Latin America | Panama | 42 (1.76) | 85 (2.44) | 115 (2.77) | 1.62 (1.33 - 1.92) | 1.47 (1.00 - 1.94) |
| Central Latin America | Venezuela | 402 (2.14) | 626 (2.25) | 948 (3.38) | 1.55 (0.78 - 2.32) | 3.93 (2.59 - 5.29) |
| Tropical Latin America | Brazil | 2,046 (1.37) | 3,570 (1.80) | 4,751 (2.19) | 1.66 (1.30 - 2.02) | 2.26 (1.49 - 3.04) |
| Tropical Latin America | Paraguay | 26 (0.65) | 51 (0.84) | 77 (1.11) | 1.94 (1.55 - 2.32) | 3.22 (2.46 - 3.99) |
| North Africa and Middle East | Algeria | 208 (0.82) | 346 (0.96) | 550 (1.32) | 1.63 (1.57 - 1.70) | 3.51 (3.37 - 3.66) |
| North Africa and Middle East | Bahrain | 5 (1.01) | 12 (0.99) | 21 (1.46) | 1.07 (0.63 - 1.50) | 4.00 (3.04 - 4.96) |
| North Africa and Middle East | Egypt | 3,175 (5.70) | 5,813 (6.94) | 7,572 (7.64) | 1.05 (0.85 - 1.26) | 1.06 (0.93 - 1.20) |
| North Africa and Middle East | Iran | 449 (0.77) | 879 (1.15) | 1,269 (1.51) | 2.34 (2.20 - 2.49) | 3.08 (2.80 - 3.35) |
| North Africa and Middle East | Iraq | 147 (0.84) | 290 (0.85) | 422 (1.00) | 0.58 (0.31 - 0.84) | 1.55 (1.23 - 1.87) |
| North Africa and Middle East | Jordan | 25 (0.67) | 51 (0.70) | 95 (0.81) | 0.69 (0.51 - 0.86) | 1.59 (1.34 - 1.85) |
| North Africa and Middle East | Kuwait | 7 (0.38) | 21 (0.70) | 32 (0.72) | 2.44 (1.58 - 3.31) | 0.13 (-1.26 - 1.54) |
| North Africa and Middle East | Lebanon | 37 (1.14) | 70 (1.68) | 88 (1.70) | 1.38 (1.20 - 1.56) | 0.03 (-0.39 - 0.45) |
| North Africa and Middle East | Libya | 46 (1.10) | 85 (1.39) | 122 (1.81) | 1.78 (1.51 - 2.04) | 2.94 (2.55 - 3.34) |
| North Africa and Middle East | Morocco | 252 (1.00) | 439 (1.31) | 620 (1.72) | 1.86 (1.57 - 2.15) | 3.15 (2.41 - 3.89) |
| North Africa and Middle East | Palestine | 24 (1.17) | 41 (0.99) | 54 (1.10) | -0.21 (-0.39 - -0.02) | 1.30 (1.15 - 1.45) |
| North Africa and Middle East | Oman | 15 (0.76) | 32 (1.13) | 40 (0.87) | 0.51 (0.05 - 0.97) | -2.68 (-3.42 - -1.93) |
| North Africa and Middle East | Qatar | 5 (1.02) | 17 (1.00) | 31 (1.10) | 0.14 (-0.37 - 0.65) | 0.57 (0.00 - 1.14) |
| North Africa and Middle East | Saudi Arabia | 252 (1.57) | 377 (1.35) | 511 (1.43) | -0.38 (-0.59 - -0.18) | 0.50 (0.24 - 0.77) |
| North Africa and Middle East | Syria | 134 (1.04) | 236 (1.13) | 292 (2.02) | 2.35 (2.08 - 2.63) | 6.98 (6.35 - 7.62) |
| North Africa and Middle East | Tunisia | 81 (0.96) | 156 (1.44) | 200 (1.73) | 2.04 (1.93 - 2.14) | 2.13 (2.07 - 2.19) |
| North Africa and Middle East | Turkey | 515 (0.86) | 875 (1.17) | 1,265 (1.56) | 2.09 (1.91 - 2.28) | 3.29 (3.17 - 3.42) |
| North Africa and Middle East | United Arab Emirates | 7 (0.38) | 30 (0.36) | 72 (0.78) | 2.53 (2.19 - 2.86) | 8.87 (8.18 - 9.56) |
| North Africa and Middle East | Yemen | 119 (0.87) | 213 (0.85) | 292 (0.93) | 0.25 (0.11 - 0.38) | 1.02 (0.86 - 1.17) |
| North Africa and Middle East | Afghanistan | 262 (2.29) | 329 (1.15) | 427 (1.12) | -2.43 (-2.78 - -2.09) | -0.25 (-0.65 - 0.15) |
| North Africa and Middle East | Sudan | 248 (1.23) | 367 (1.09) | 486 (1.19) | -0.08 (-0.16 - 0.01) | 1.13 (1.02 - 1.24) |
| Central Sub-Saharan Africa | Angola | 154 (1.49) | 264 (1.21) | 380 (1.26) | -0.59 (-1.01 - -0.16) | 0.66 (-0.38 - 1.71) |
| Central Sub-Saharan Africa | Central African Republic | 42 (1.53) | 59 (1.28) | 76 (1.44) | -0.28 (-0.46 - -0.11) | 1.01 (0.57 - 1.45) |
| Central Sub-Saharan Africa | Congo | 40 (1.64) | 63 (1.49) | 83 (1.59) | 0.04 (-0.33 - 0.41) | 0.86 (0.24 - 1.48) |
| Central Sub-Saharan Africa | Democratic Republic of the Congo | 480 (1.24) | 709 (1.04) | 972 (1.11) | -0.36 (-0.55 - -0.16) | 1.02 (0.61 - 1.44) |
| Central Sub-Saharan Africa | Equatorial Guinea | 5 (1.26) | 7 (0.68) | 10 (0.69) | -2.04 (-2.29 - -1.80) | 0.12 (-0.28 - 0.52) |
| Central Sub-Saharan Africa | Gabon | 18 (1.85) | 25 (1.67) | 32 (1.83) | 0.01 (-0.10 - 0.12) | 0.94 (0.70 - 1.18) |
| Eastern Sub-Saharan Africa | Burundi | 110 (1.97) | 118 (1.31) | 163 (1.36) | -1.34 (-1.61 - -1.07) | 0.46 (0.19 - 0.73) |
| Eastern Sub-Saharan Africa | Comoros | 8 (1.68) | 12 (1.91) | 17 (2.39) | 1.20 (0.65 - 1.76) | 2.38 (1.15 - 3.62) |
| Eastern Sub-Saharan Africa | Djibouti | 5 (1.01) | 12 (1.34) | 20 (1.66) | 1.76 (1.48 - 2.05) | 2.59 (2.00 - 3.18) |
| Eastern Sub-Saharan Africa | Eritrea | 44 (1.46) | 99 (1.75) | 139 (2.07) | 1.21 (0.98 - 1.45) | 1.68 (1.02 - 2.35) |
| Eastern Sub-Saharan Africa | Ethiopia | 1,087 (2.12) | 1,218 (1.42) | 1,589 (1.48) | -1.27 (-1.38 - -1.15) | 0.49 (0.20 - 0.78) |
| Eastern Sub-Saharan Africa | Kenya | 527 (2.27) | 1,087 (2.67) | 1,485 (2.96) | 0.89 (0.75 - 1.03) | 1.07 (0.77 - 1.38) |
| Eastern Sub-Saharan Africa | Madagascar | 204 (1.71) | 291 (1.38) | 395 (1.48) | -0.49 (-0.58 - -0.40) | 0.73 (0.49 - 0.97) |
| Eastern Sub-Saharan Africa | Malawi | 213 (2.23) | 260 (1.82) | 326 (1.77) | -0.71 (-0.94 - -0.48) | -0.24 (-0.52 - 0.04) |
| Eastern Sub-Saharan Africa | Mozambique | 130 (0.99) | 217 (0.94) | 246 (0.83) | -0.70 (-0.93 - -0.48) | -1.33 (-1.66 - -0.99) |
| Eastern Sub-Saharan Africa | Rwanda | 191 (2.66) | 163 (1.58) | 242 (1.91) | -1.12 (-1.33 - -0.90) | 2.03 (1.79 - 2.27) |
| Eastern Sub-Saharan Africa | Somalia | 121 (1.69) | 223 (1.51) | 290 (1.43) | -0.64 (-0.80 - -0.49) | -0.61 (-0.66 - -0.56) |
| Eastern Sub-Saharan Africa | Tanzania | 400 (1.54) | 644 (1.44) | 781 (1.38) | -0.46 (-0.64 - -0.28) | -0.69 (-0.90 - -0.47) |
| Eastern Sub-Saharan Africa | Uganda | 235 (1.36) | 354 (1.09) | 507 (1.23) | -0.35 (-0.47 - -0.23) | 1.56 (1.29 - 1.82) |
| Eastern Sub-Saharan Africa | Zambia | 171 (2.15) | 262 (1.92) | 386 (2.12) | -0.02 (-0.21 - 0.16) | 1.23 (0.88 - 1.58) |
| Eastern Sub-Saharan Africa | South Sudan | 93 (1.59) | 117 (1.25) | 135 (1.45) | -0.31 (-0.49 - -0.13) | 1.76 (1.23 - 2.29) |
| Southern Sub-Saharan Africa | Botswana | 17 (1.27) | 32 (1.60) | 39 (1.69) | 1.03 (0.91 - 1.15) | 0.59 (0.48 - 0.70) |
| Southern Sub-Saharan Africa | Lesotho | 26 (1.43) | 42 (2.14) | 52 (2.50) | 1.99 (1.78 - 2.21) | 1.84 (1.33 - 2.36) |
| Southern Sub-Saharan Africa | Namibia | 17 (1.23) | 25 (1.20) | 34 (1.43) | 0.49 (0.29 - 0.69) | 1.87 (1.47 - 2.27) |
| Southern Sub-Saharan Africa | South Africa | 492 (1.34) | 915 (1.80) | 899 (1.62) | 0.67 (0.28 - 1.07) | -0.99 (-1.33 - -0.64) |
| Southern Sub-Saharan Africa | Swaziland | 10 (1.21) | 27 (2.52) | 28 (2.44) | 2.39 (2.17 - 2.60) | -0.45 (-0.63 - -0.27) |
| Southern Sub-Saharan Africa | Zimbabwe | 138 (1.33) | 257 (1.97) | 268 (1.79) | 1.06 (0.76 - 1.37) | -1.10 (-1.80 - -0.39) |
| Western Sub-Saharan Africa | Benin | 85 (1.76) | 135 (1.44) | 182 (1.44) | -0.66 (-0.73 - -0.59) | 0.05 (-0.06 - 0.15) |
| Western Sub-Saharan Africa | Burkina Faso | 158 (1.65) | 171 (1.01) | 257 (1.13) | -1.09 (-1.74 - -0.44) | 1.95 (0.67 - 3.24) |
| Western Sub-Saharan Africa | Cameroon | 157 (1.51) | 254 (1.15) | 331 (1.14) | -0.97 (-1.09 - -0.86) | -0.14 (-0.44 - 0.16) |
| Western Sub-Saharan Africa | Cape Verde | 5 (1.29) | 10 (1.99) | 16 (2.90) | 2.88 (2.38 - 3.38) | 4.58 (4.20 - 4.96) |
| Western Sub-Saharan Africa | Chad | 110 (1.82) | 168 (1.43) | 215 (1.31) | -1.13 (-1.27 - -0.99) | -1.03 (-1.39 - -0.67) |
| Western Sub-Saharan Africa | Cote dIvoire | 157 (1.29) | 292 (1.35) | 332 (1.27) | -0.05 (-0.24 - 0.14) | -0.65 (-0.89 - -0.41) |
| Western Sub-Saharan Africa | The Gambia | 19 (1.91) | 47 (2.64) | 63 (2.80) | 1.40 (0.72 - 2.08) | 1.18 (0.46 - 1.91) |
| Western Sub-Saharan Africa | Ghana | 296 (1.97) | 434 (1.72) | 593 (1.88) | -0.18 (-0.33 - -0.03) | 1.15 (0.82 - 1.48) |
| Western Sub-Saharan Africa | Guinea | 184 (2.98) | 290 (2.90) | 321 (2.54) | -0.46 (-0.60 - -0.32) | -1.20 (-1.49 - -0.91) |
| Western Sub-Saharan Africa | Guinea-Bissau | 21 (2.11) | 29 (1.88) | 39 (2.05) | -0.22 (-0.39 - -0.05) | 0.61 (0.18 - 1.04) |
| Western Sub-Saharan Africa | Liberia | 51 (2.62) | 69 (1.70) | 83 (1.74) | -1.46 (-1.76 - -1.15) | -0.15 (-0.79 - 0.50) |
| Western Sub-Saharan Africa | Mali | 209 (2.41) | 260 (1.63) | 338 (1.54) | -1.50 (-1.59 - -1.41) | -0.57 (-0.79 - -0.36) |
| Western Sub-Saharan Africa | Mauritania | 51 (2.45) | 52 (1.57) | 63 (1.57) | -1.50 (-1.66 - -1.35) | -0.03 (-0.32 - 0.27) |
| Western Sub-Saharan Africa | Niger | 100 (1.25) | 164 (0.99) | 242 (1.04) | -0.70 (-0.86 - -0.54) | 0.40 (-0.04 - 0.83) |
| Western Sub-Saharan Africa | Nigeria | 1,889 (2.10) | 2,857 (1.71) | 3,341 (1.56) | -1.06 (-1.28 - -0.83) | -1.20 (-1.44 - -0.96) |
| Western Sub-Saharan Africa | Sao Tome and Principe | 3 (2.37) | 4 (2.04) | 4 (2.12) | -0.29 (-0.47 - -0.11) | 0.62 (0.24 - 1.01) |
| Western Sub-Saharan Africa | Senegal | 104 (1.37) | 152 (1.21) | 218 (1.44) | 0.07 (-0.69 - 0.84) | 1.65 (-0.07 - 3.39) |
| Western Sub-Saharan Africa | Sierra Leone | 75 (2.06) | 80 (1.26) | 108 (1.31) | -1.49 (-1.82 - -1.16) | 0.55 (-0.24 - 1.34) |
| Western Sub-Saharan Africa | Togo | 51 (1.40) | 89 (1.39) | 117 (1.48) | 0.08 (-0.14 - 0.31) | 0.56 (-0.08 - 1.20) |
| Abbreviations: CI, confidence interval; APC, Annual Percent Change | | | | | | |

S Table 9. GBD Country with Data Quality Rating for Causes of Death Data Star-rating system based on the proportion of death registered to a well-defined cause (percent well-certified): 5 stars: 85%–100% well-certified;4 stars: 65%–84%, 3 stars: 35%–64%, 2 stars: 10%–34%, 1 star: >0%–9%, 0 stars: No VR or VA data available from 2010–2019

| **Country** | **Percent well-certified (2010-2019)** | **Data Quality Rating** |
| --- | --- | --- |
| Afghanistan | 0.0 | 1 |
| Albania | 54.6 | 3 |
| Algeria | 0.0 | 1 |
| American Samoa | 66.2 | 3 |
| Andorra | 65.3 | 1 |
| Angola | 2.8 | 1 |
| Antigua and Barbuda | 78.6 | 4 |
| Argentina | 73.7 | 4 |
| Armenia | 89.6 | 5 |
| Australia | 91.1 | 5 |
| Austria | 91.1 | 5 |
| Azerbaijan | 0.0 | 3 |
| Bahrain | 54.9 | 3 |
| Bangladesh | 53.3 | 2 |
| Barbados | 76.8 | 4 |
| Belarus | 83.7 | 4 |
| Belgium | 82.9 | 4 |
| Belize | 87.6 | 4 |
| Benin | 0.0 | 1 |
| Bermuda | 90.4 | 5 |
| Bhutan | 0.0 | 0 |
| Venezuela | 91.3 | 5 |
| Bosnia and Herzegovina | 75.4 | 2 |
| Botswana | 0.0 | 0 |
| Brazil | 82.3 | 4 |
| Brunei | 73.8 | 3 |
| Bulgaria | 68.1 | 4 |
| Burkina Faso | 0.3 | 1 |
| Burundi | 0.0 | 1 |
| Cambodia | 0.0 | 1 |
| Cameroon | 0.0 | 0 |
| Canada | 90.2 | 5 |
| Central African Republic | 0.0 | 0 |
| Chad | 0.0 | 0 |
| Chile | 90.4 | 4 |
| China | 71.7 | 3 |
| Colombia | 90.8 | 4 |
| The Bahamas | 82.5 | 4 |
| Comoros | 0.0 | 0 |
| Congo (Brazzaville) | 0.0 | 0 |
| Cook Islands | 68.5 | 2 |
| Costa Rica | 91.6 | 5 |
| Croatia | 93.7 | 4 |
| Cuba | 92.3 | 5 |
| Cyprus | 78.4 | 2 |
| Czech Republic | 87.9 | 4 |
| North Korea | 0.0 | 0 |
| DR Congo | 0.0 | 1 |
| Denmark | 85.1 | 5 |
| Djibouti | 0.0 | 0 |
| Dominica | 74.2 | 3 |
| Dominican Republic | 45.6 | 3 |
| Ecuador | 63.4 | 3 |
| Egypt | 46.6 | 2 |
| El Salvador | 65.4 | 3 |
| Equatorial Guinea | 0.0 | 0 |
| Eritrea | 0.0 | 0 |
| Estonia | 91.9 | 5 |
| Ethiopia | 4.0 | 1 |
| Federated States of Micronesia | 0.0 | 0 |
| Fiji | 66.0 | 2 |
| Finland | 94.4 | 5 |
| France | 80.7 | 4 |
| Gabon | 0.0 | 0 |
| Georgia | 58.9 | 4 |
| Germany | 85.6 | 4 |
| Ghana | 0.4 | 1 |
| Greece | 82.7 | 4 |
| Greenland | 83.6 | 3 |
| Grenada | 87.5 | 4 |
| Guam | 63.3 | 3 |
| Guatemala | 77.7 | 4 |
| Guinea | 0.0 | 1 |
| Guinea-Bissau | 0.0 | 1 |
| Guyana | 74.2 | 4 |
| Haiti | 0.0 | 1 |
| Honduras | 13.0 | 2 |
| Hungary | 94.2 | 5 |
| Iceland | 91.3 | 5 |
| India | 46.0 | 2 |
| Indonesia | 63.4 | 2 |
| Iraq | 46.2 | 2 |
| Ireland | 92.9 | 5 |
| Iran | 80.0 | 2 |
| Israel | 82.3 | 4 |
| Italy | 89.8 | 5 |
| Jamaica | 89.4 | 4 |
| Japan | 85.5 | 5 |
| Jordan | 65.2 | 2 |
| Kazakhstan | 73.6 | 4 |
| Kenya | 23.4 | 1 |
| eSwatini | 0.0 | 1 |
| Kiribati | 0.0 | 2 |
| Kuwait | 68.5 | 4 |
| Kyrgyzstan | 94.1 | 4 |
| Laos | 0.0 | 1 |
| Latvia | 93.8 | 5 |
| Lebanon | 0.0 | 1 |
| Lesotho | 0.0 | 0 |
| Liberia | 0.0 | 1 |
| Libya | 0.0 | 1 |
| Lithuania | 93.5 | 5 |
| Luxembourg | 83.1 | 4 |
| Madagascar | 0.0 | 1 |
| Malawi | 0.6 | 1 |
| Malaysia | 40.4 | 2 |
| Maldives | 61.9 | 2 |
| Mali | 5.6 | 1 |
| Malta | 93.5 | 5 |
| Marshall Islands | 0.0 | 0 |
| Mauritania | 0.0 | 0 |
| Mauritius | 87.0 | 4 |
| Mexico | 89.4 | 4 |
| Mongolia | 79.4 | 2 |
| Montenegro | 0.0 | 2 |
| Morocco | 12.1 | 2 |
| Mozambique | 3.5 | 2 |
| Myanmar | 48.9 | 1 |
| Namibia | 0.0 | 0 |
| Nepal | 0.0 | 1 |
| Netherlands | 84.2 | 5 |
| New Zealand | 96.0 | 5 |
| Nicaragua | 79.5 | 3 |
| Niger | 0.0 | 1 |
| Nigeria | 40.4 | 1 |
| Northern Mariana Islands | 64.9 | 2 |
| Norway | 85.7 | 5 |
| Oman | 32.4 | 2 |
| Pakistan | 21.6 | 2 |
| Palestine | 72.8 | 2 |
| Panama | 84.6 | 4 |
| Papua New Guinea | 29.5 | 1 |
| Paraguay | 82.2 | 3 |
| Peru | 52.1 | 3 |
| Philippines | 75.4 | 3 |
| Bolivia | 0.0 | 1 |
| Poland | 75.9 | 4 |
| Portugal | 86.0 | 4 |
| Monaco | 80.3 | 2 |
| Puerto Rico | 84.5 | 4 |
| Qatar | 38.9 | 2 |
| Cape Verde | 70.2 | 2 |
| Côte d'Ivoire | 0.1 | 1 |
| South Korea | 84.0 | 3 |
| Moldova | 90.1 | 5 |
| Nauru | 0.0 | 0 |
| Niue | 0.0 | 0 |
| Palau | 51.0 | 1 |
| San Marino | 65.8 | 3 |
| The Gambia | 0.0 | 1 |
| Romania | 86.8 | 4 |
| Russia | 88.5 | 5 |
| Rwanda | 0.0 | 1 |
| Saint Kitts and Nevis | 84.8 | 4 |
| Saint Lucia | 86.5 | 4 |
| Saint Vincent and the Grenadines | 87.2 | 4 |
| Samoa | 0.0 | 0 |
| São Tomé and PrÍncipe | 0.0 | 1 |
| Saudi Arabia | 20.6 | 2 |
| Senegal | 0.0 | 1 |
| Serbia | 81.4 | 3 |
| Seychelles | 79.4 | 3 |
| Sierra Leone | 0.0 | 1 |
| Singapore | 93.8 | 5 |
| Slovakia | 92.0 | 3 |
| Slovenia | 87.0 | 4 |
| Vietnam | 4.8 | 2 |
| Solomon Islands | 38.0 | 1 |
| Somalia | 0.0 | 0 |
| South Africa | 69.1 | 3 |
| South Sudan | 0.0 | 0 |
| Spain | 88.0 | 4 |
| Sri Lanka | 65.3 | 3 |
| Sudan | 0.0 | 0 |
| Suriname | 67.1 | 3 |
| Sweden | 86.0 | 5 |
| Switzerland | 87.5 | 4 |
| Syria | 52.5 | 3 |
| Taiwan (province of China) | 85.6 | 4 |
| Tajikistan | 50.5 | 3 |
| Thailand | 64.6 | 3 |
| North Macedonia | 68.8 | 3 |
| Timor-Leste | 0.0 | 0 |
| Togo | 0.0 | 0 |
| Tokelau | 0.0 | 0 |
| Tonga | 0.0 | 1 |
| Trinidad and Tobago | 89.8 | 5 |
| Tunisia | 24.0 | 1 |
| Turkey | 79.5 | 3 |
| Turkmenistan | 82.0 | 4 |
| Tuvalu | 0.0 | 0 |
| Uganda | 0.0 | 1 |
| Ukraine | 91.2 | 5 |
| United Arab Emirates | 0.0 | 1 |
| UK | 91.6 | 5 |
| Northern Ireland | 92.8 | 5 |
| Scotland | 91.4 | 5 |
| Wales | 91.7 | 5 |
| England | 91.6 | 5 |
| Tanzania | 0.0 | 1 |
| Virgin Islands | 61.2 | 3 |
| USA | 87.2 | 5 |
| Uruguay | 80.4 | 4 |
| Uzbekistan | 73.0 | 4 |
| Vanuatu | 0.0 | 0 |
| Yemen | 0.0 | 0 |
| Zambia | 15.7 | 1 |
| Zimbabwe | 0.0 | 2 |

VR: vital registration systems; Verbal autopsy (VA), a method in which a standardized interview collects information from an individual familiar with the deceased to determine individuals’ causes of death and cause-specific mortality fractions in populations without adequate vital registration systems, was collected by using the SmartVA-Analyze Application of implementing the Tariff 2.0 Method, which validated with the Population Health Metrics Research Consortium (PHMRC).
