# Supplementary figures and images for: The burden of nonalcoholic fatty liver disease (NAFLD) is rapidly growing in every region of the world from 1990 to 2019
Source: Hepatol Commun. 2023 Oct 2;7(10):e0251. doi: 10.1097/HC9.0000000000000251 (PMC10545420; doi:10.1097/HC9.0000000000000251)

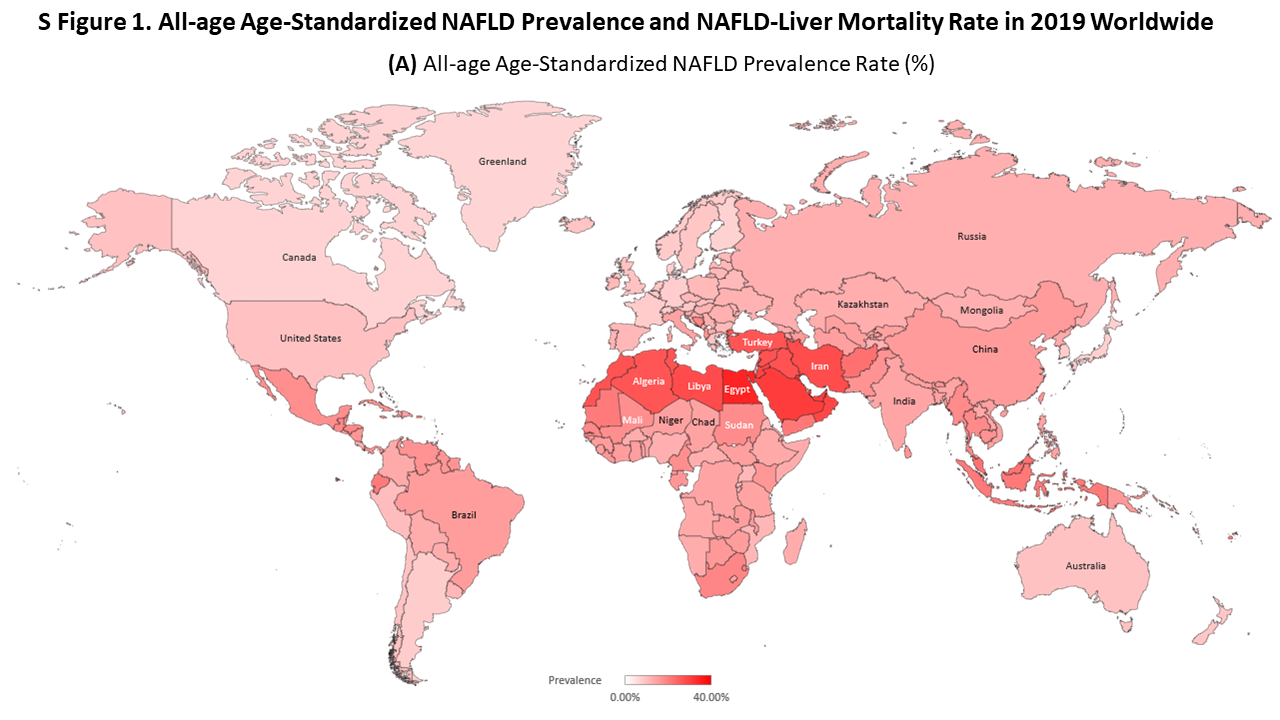

Supplement: SUPPLEMENTARY MATERIAL [file hc9-7-e0251-s002.tif]

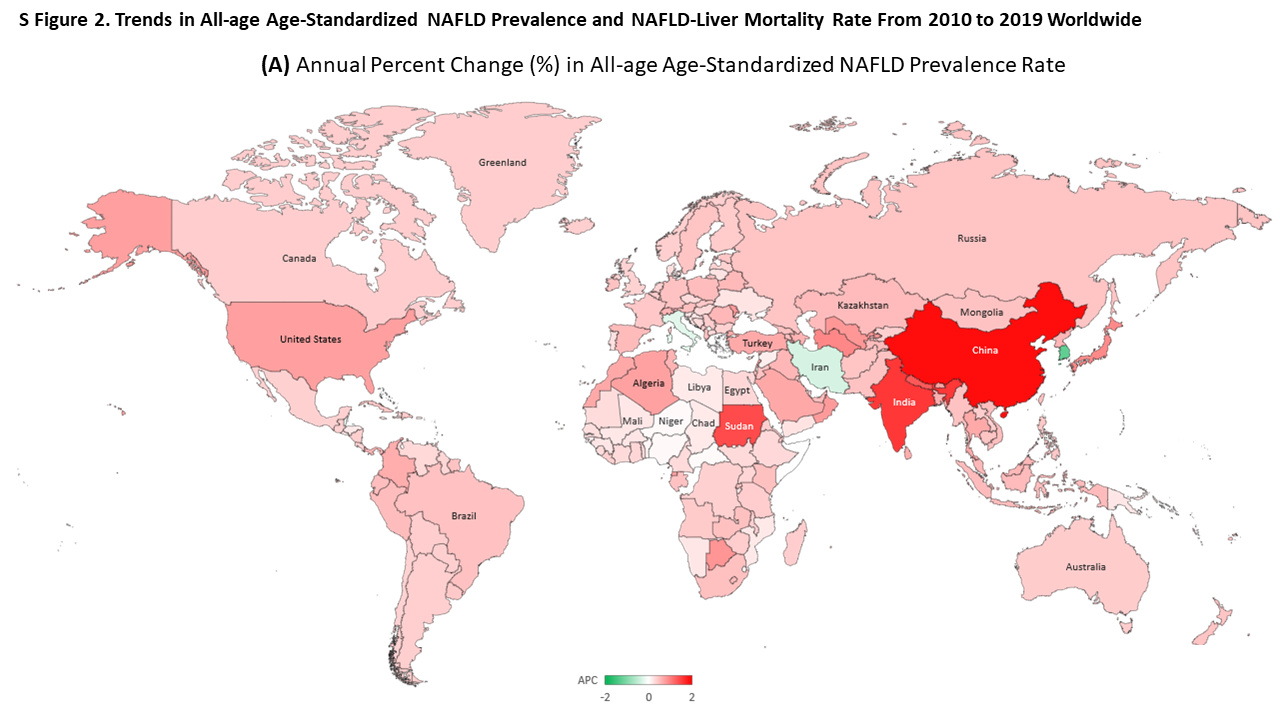

Supplement: SUPPLEMENTARY MATERIAL [file hc9-7-e0251-s003.tif]

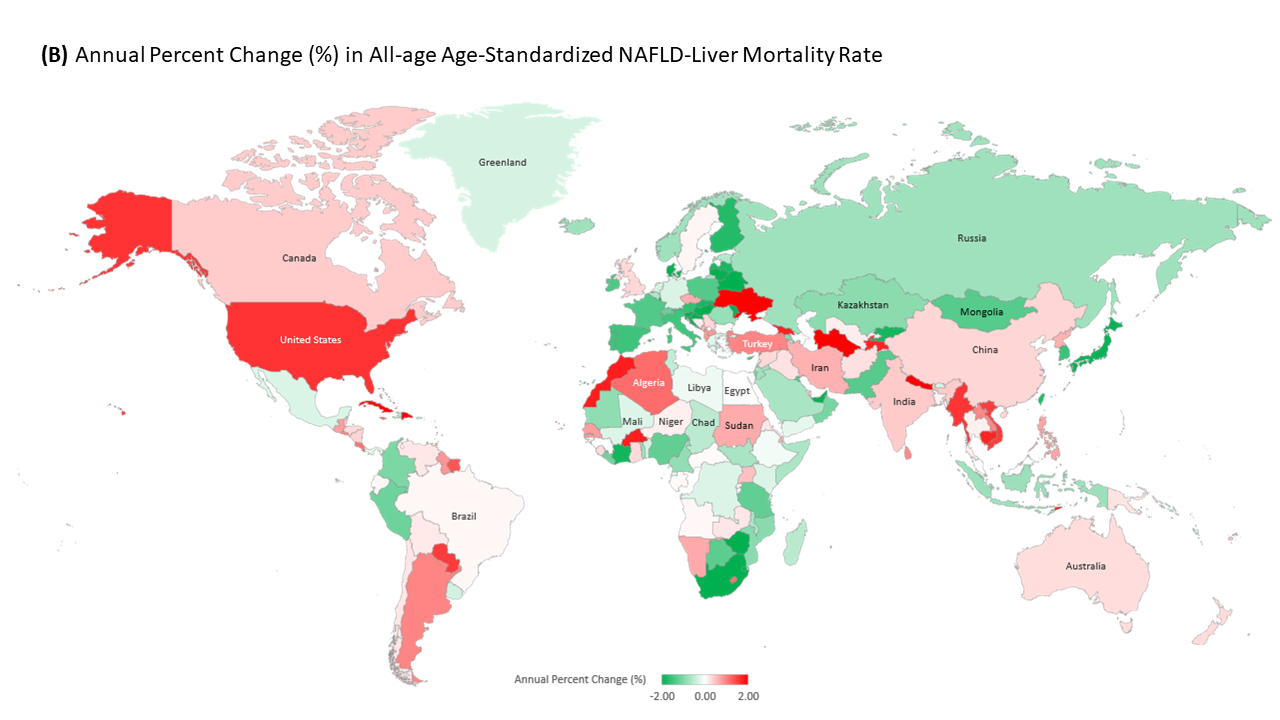

Supplement: SUPPLEMENTARY MATERIAL [file hc9-7-e0251-s004.tif]

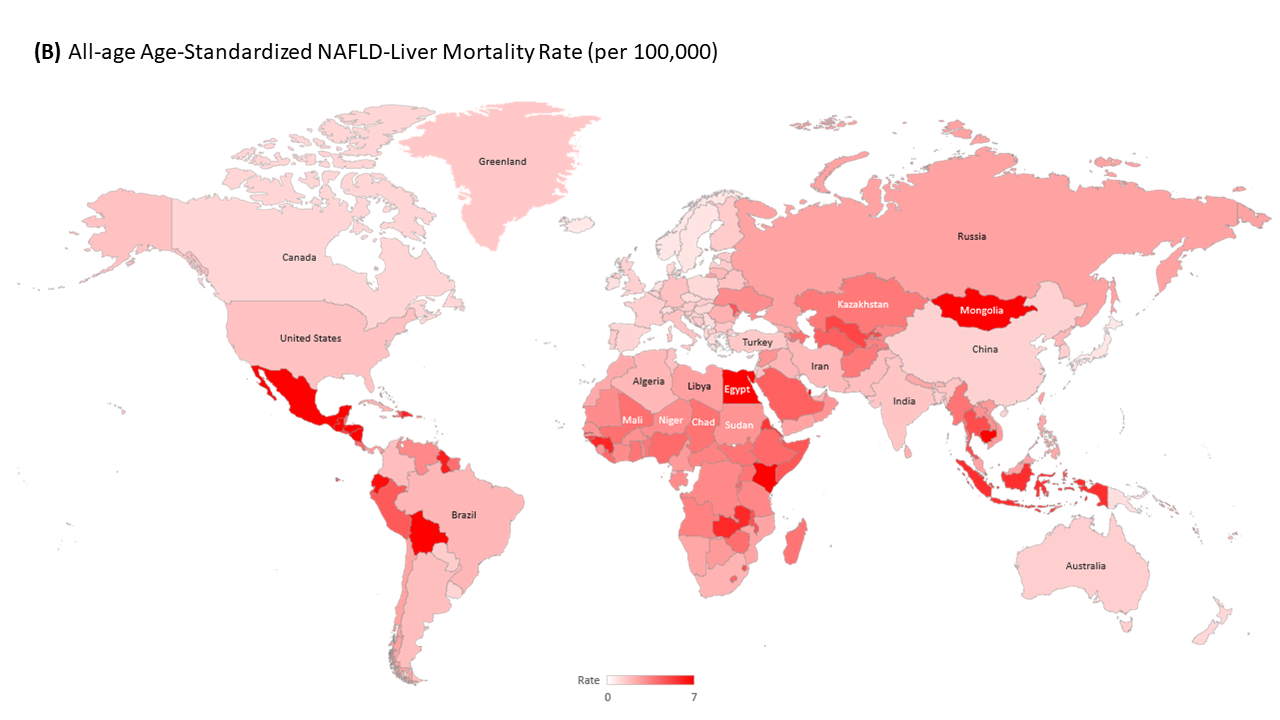

Supplement: SUPPLEMENTARY MATERIAL [file hc9-7-e0251-s005.tif]
